# MECOA: A Multi-Strategy Enhanced Coati Optimization Algorithm for Global Optimization and Photovoltaic Models Parameter Estimation

**DOI:** 10.3390/biomimetics10120839

**Published:** 2025-12-15

**Authors:** Hang Chen, Maomao Luo

**Affiliations:** 1 General Education School, Xi’an Eurasia University, Xi’an 710065, China; chenhang@eurasia.edu; 2 General College of Busan Foreign Studies University, Busan 46234, Republic of Korea; 3 Public Education Department, Jiujiang Polytechnic University of Science and Technology, Jiujiang 332020, China; 4 College of Economics and Management, Shenyang Aerospace University, Shenyang 110136, China

**Keywords:** photovoltaic models, coati optimization algorithm, global optimization, exploration-exploitation, parameter estimation

## Abstract

To address the limitations of the traditional Coati Optimization Algorithm (COA), such as insufficient global exploration, poor population cooperation, and low convergence efficiency in global optimization and photovoltaic (PV) model parameter identification, this paper proposes a Multi-strategy Enhanced Coati Optimization Algorithm (MECOA). MECOA improves performance through three core strategies: (1) Elite-guided search, which replaces the single global best solution with an elite pool of three top individuals and incorporates the heavy-tailed property of Lévy flights to balance large-step exploration and small-step exploitation; (2) Horizontal crossover, which simulates biological gene recombination to promote information sharing among individuals and enhance cooperative search efficiency; and (3) Precise elimination, which discards 20% of low-fitness individuals in each generation and generates new individuals around the best solution to improve population quality. Experiments on the CEC2017 (30/50/100-dimensional) and CEC2022 (20-dimensional) benchmark suites demonstrate that MECOA achieves superior performance. On CEC2017, MECOA ranks first with an average rank of 1.87, 2.07, 1.83, outperforming the second-best LSHADE (2.03, 2.43 and 2.63) and the original COA (9.93, 9.93 and 9.96). On CEC2022, MECOA also maintains the leading position with an average rank of 1.58, far surpassing COA (8.92). Statistical analysis using the Wilcoxon rank-sum test (significance level 0.05) confirms the superiority of MECOA. Furthermore, MECOA is applied to parameter identification of single-diode (SDM) and double-diode (DDM) PV models. Experiments based on real measurement data show that the SDM model achieves an RMSE of 9.8610 × 10^−4^, which is only 1/20 of that of COA. For the DDM model, the fitted curves almost perfectly overlap with the experimental data, with a total integrated absolute error (IAE) of only 0.021555 A. These results fully validate the effectiveness and reliability of MECOA in solving complex engineering optimization problems, providing a robust and efficient solution for accurate modeling and optimization of PV systems.

## 1. Introduction

The continuous growth of global energy demand and the environmental issues caused by excessive fossil fuel consumption have accelerated the rapid development of clean energy technologies. Among them, solar energy—a clean, renewable, and widely distributed resource—has become a vital component of global power generation, with photovoltaic (PV) systems serving as its core utilization platform [1,2]. PV systems directly convert solar energy into electricity through PV cells, and the accurate modeling and optimization of their operating performance are essential prerequisites for efficient utilization, fault diagnosis, and energy management. This process, however, critically depends on both the construction of PV models and the precise identification of their unknown parameters [3].

To characterize the electrical output behavior of PV cells and modules, various equivalent circuit models have been proposed. Among them, the single-diode model (SDM) and double-diode model (DDM) are the most widely adopted due to their balance between modeling simplicity and fitting accuracy. The SDM represents the core operating mechanism of PV devices using a photocurrent source, a diode, a series resistance, and a shunt resistance. It requires the estimation of five parameters: photocurrent $\left(I_{ph}\right)$, diode reverse saturation current $\left(I_{d}\right)$, diode ideality factor $\left(n\right)$, series resistance $\left(R_{s}\right)$, and shunt resistance $\left(R_{sh}\right)$. The DDM further accounts for recombination losses in the depletion region of the semiconductor by introducing a second diode, thus adding two more parameters—the saturation current of the second diode $\left(I_{d2}\right)$ and the diffusion factor $\left(n_{2}\right)$—bringing the total to seven [4,5,6]. However, the mathematical formulations of both models involve implicit transcendental equations with strong inter-parameter coupling, and the parameter values are highly sensitive to external conditions. Consequently, parameter extraction for PV models faces the triple challenge of high dimensionality, strong nonlinearity, and multiple constraints. These factors make it difficult for traditional methods to achieve high-precision identification, thereby limiting the effectiveness of PV system modeling and optimization in practice [7,8].

To address the challenges of PV model parameter identification, researchers have explored various approaches, including analytical methods, direct methods, numerical computation methods, and traditional optimization algorithms. Analytical methods derive parameter expressions by simplifying the model, offering fast computation but with accuracy often compromised by simplifying assumptions. Direct methods rely on fitting experimental data, making them highly sensitive to measurement errors. Numerical computation methods (e.g., Newton–Raphson) are constrained by initial value settings and prone to being trapped in local optima. Traditional optimization algorithms are limited by objective function constraints, which restrict their applicability [9,10,11]. In contrast, swarm intelligence (SI) algorithms have gradually become the mainstream technology in PV parameter identification due to their advantages: independence from gradient information, strong robustness, and the ability to approximate global optima within reasonable computational time. Notable progress has been made in this area. For example, Abido et al. [12] were the first to apply the Differential Evolution (DE) algorithm to parameter estimation of the seven-parameter PV circuit model, demonstrating the feasibility of SI algorithms in this domain. Similarly, Shayeghi et al. [13] enhanced the discrete Particle Swarm Optimization (PSO) algorithm by introducing an anti-premature convergence mechanism, which not only improved algorithmic performance but also provided an efficient solution to planning problems related to PV systems.

With the advancement of research, an increasing number of swarm intelligence (SI) algorithms have been tailored for PV model parameter extraction. Elazab et al. were the first to introduce the Whale Optimization Algorithm (WOA) into PV systems, achieving a balance between low cost and high accuracy [14]. For more complex scenarios such as the double-diode model, Abbassi et al. applied the Salp Swarm Algorithm (SSA) as an optimizer, confirming its strong competitiveness [15]. Zhang et al. combined the Orthogonal Learning (OL) strategy with the Nelder–Mead simplex (NMs) method to develop the NMSOLMFO algorithm, which achieved remarkable results in parameter identification of SDM, DDM, and PV module models [16]. Oliva et al. improved WOA by incorporating chaotic mapping and adaptive strategies, and the resulting Chaotic Whale Optimization Algorithm (CWOA) demonstrated outstanding performance in PV parameter estimation [17]. Wu et al. integrated chaotic sequences and the PSO concept into the Antlion Optimizer (ALO), proposing the Improved Antlion Optimizer (IALO), which significantly enhanced identification accuracy [18].

In addition, research on dynamic PV models has gradually expanded. Su et al. established a dynamic modeling framework for variable-speed PV DC systems and PV plant clusters, providing a foundation for system stability analysis and control strategy validation [19]. Di et al. employed least squares regression to extract parameters of dynamic PV models [20]. Li et al. developed a Memetic Adaptive Differential Evolution (MADE) algorithm to estimate unknown parameters of different PV models [21]. Mohamed Abdel-Basset proposed an improved variant of the marine predators algorithm for optimal PV parameter extraction [22]. Jiao et al. introduced an Enhanced Harris Hawks Optimization (EHHO) algorithm, which integrates OL and General Opposition-Based Learning (GOBL) to accurately estimate the parameters of solar cells and PV modules [23]. Yu et al. designed a novel differential evolution variant (PDcDE) based on automatic control population strategies and diversity-controlled scaling factor settings to solve PV model parameter estimation problems [24]. Furthermore, Premkumar et al. hybridized the Cross-Crossover (CC) algorithm and the Nelder–Mead simplex (NM) strategy with the Gradient-Based Optimizer (GBO) to enhance its performance in estimating uncertain PV model parameters [25]. Although these methods have proven effective in extracting unknown parameters of PV models, they still suffer from the risk of being trapped in local optima.

The Coati Optimization Algorithm (COA) [26] is a recently proposed bio-inspired metaheuristic algorithm, drawing inspiration from two core behaviors of coatis in nature: their cooperative encirclement when hunting iguanas (corresponding to the exploration phase of the algorithm) and their local escape behavior when evading predators (corresponding to the exploitation phase). Compared with conventional algorithms, COA features a simple structure, few parameters, and ease of implementation. It has already shown certain advantages in some low-dimensional optimization problems [27]. However, COA still exhibits clear performance limitations in complex optimization scenarios. During the exploration phase, it relies solely on a simple linear random perturbation that guides solutions toward a single global best. The limited, uniformly distributed step sizes make it difficult to overcome local optima in multimodal functions. Moreover, excessive dependence on a single best solution reduces population diversity, often leading to premature convergence. In addition, the lack of information-sharing mechanisms among individuals results in low utilization of population resources and prevents effective cooperative search, thereby constraining both convergence speed and accuracy. Furthermore, the use of a fixed population size allows low-fitness individuals to persist, occupying computational resources and potentially misleading the search direction [28,29,30]. These shortcomings hinder COA’s ability to balance global exploration and local exploitation in high-dimensional, complex tasks such as PV model parameter identification, ultimately preventing it from meeting the performance requirements of practical applications.

Compared with existing COA variants, MECOA’s novelty lies in the specific combination of three complementary strategies: elite-guided search with Lévy flights balances exploration and exploitation, horizontal crossover enhances population information sharing, and precise elimination optimizes population quality. This synergy addresses COA’s inherent limitations more comprehensively than single-strategy improvements. Additionally, MECOA is the first COA variant tailored for PV model parameter identification, filling the gap between COA-based optimization and practical PV system modeling.

To address the aforementioned issues, this paper proposes a Multi-strategy Enhanced Coati Optimization Algorithm (MECOA), which overcomes the performance bottlenecks of COA through three core improvement strategies and applies it to PV model parameter identification. The main contributions and innovations of this work are as follows:

1. Elite-guided search strategy: An elite pool composed of the top three individuals replaces the single global best solution. By integrating the heavy-tailed property of Lévy flights, MECOA achieves a balance between large-step jumps and fine-grained local searches, thereby enhancing global exploration while maintaining population diversity.
2. Horizontal crossover strategy: Inspired by biological gene recombination, this strategy performs random pairing and linear combination among individuals to promote the dissemination and sharing of superior information, improving cooperative search efficiency across the population.
3. Precise elimination mechanism: At each iteration, 20% of the low-fitness individuals are discarded, and new individuals are generated around the neighborhood of the current global best solution. This not only improves population quality but also avoids ineffective searches.
4. Comprehensive benchmark validation: MECOA is compared against seven mainstream metaheuristic algorithms, including GWO, WOA, and PSO, on the CEC2017 (30/50/100-dimensional) and CEC2022 (20-dimensional) benchmark suites. The evaluation focuses on population diversity, exploration–exploitation balance, convergence speed, and accuracy, validating the superior optimization performance of MECOA.
5. Application to PV model parameter identification: MECOA is applied to both the SDM and DDM PV models. Using experimental data from the Photowatt-PWP201 PV module and the RTC France solar cell, the algorithm’s effectiveness is validated through root mean square error (RMSE), integrated absolute error (IAE), and curve-fitting comparisons, demonstrating its practical value for solving complex real-world problems.

The remainder of this paper is organized as follows: Section 2 presents the fundamental principles of the traditional COA, including population initialization, the mathematical models of the exploration and exploitation phases, and the corresponding pseudocode. Section 3 introduces the three improvement strategies of MECOA, together with the algorithmic framework and time complexity analysis. Section 4 conducts comparative experiments on standard benchmark suites, analyzing the performance differences between MECOA and other algorithms from both qualitative and quantitative perspectives. Section 5 applies MECOA to PV model parameter identification, validating its effectiveness in practical engineering problems. Section 6 concludes the study and outlines future research directions.

## 2. Coati Optimization Algorithm (COA)

The Coati Optimization Algorithm (COA) is a population-based metaheuristic inspired by the natural behavior of coatis, where each coati is regarded as a member of the algorithmic population. The position of each coati in the search space represents the values of the decision variables. During hunting, coatis exhibit two typical behaviors: (1) hunting iguanas, and (2) escaping from predators. These behaviors can be abstracted into two algorithmic phases: exploration and exploitation. The mathematical model of COA is formulated as follows [26]:

### 2.1. Initialize Population

In COA, each coati individual is regarded as a potential solution within the search space. The position of an individual corresponds to the specific values of the decision variables, thereby forming the candidate solution set of the problem. During the initialization phase, the positions of individuals in the population are randomly generated according to Equation (1):

(1) $$X_{i}=\left(ub-lb\right)\times rand\left(0,\;1\right)+lb,$$

where $X_{i}$ denotes the position of the $i^{th}$ coati in the search space, while lb and ub represent the lower and upper bounds of the problem, respectively.

### 2.2. Exploration

Based on the biological behavior of coatis hunting iguanas, COA incorporates a search space updating mechanism. In this strategy, the population is divided into two subgroups: one subgroup climbs trees to drive the iguanas to the ground, while the other remains on the ground, ready to capture them. This cooperative hunting strategy is abstracted into the algorithm’s global search mechanism, enabling efficient exploration of the solution space [26].

Assume that the first $\frac{pop}{2}$ individuals (i=1, 2,  ⋯,  ⌊pop/2⌋) perform the climbing behavior, the pop refers to the population size. Their positions are updated according to Equation (2):

(2) $$X_{i,j}\left(t+1\right)=X_{i,j}\left(t\right)+\left(X_{best,j}\left(t\right)-I\cdot X_{i,j}\left(t\right)\right)\cdot rand\left(0,\;1\right)$$

where $X_{best}$ denotes the position of the iguana in the search space (essentially the position of the best individual), j represents the dimension, and I is an integer randomly selected from the set $\left\{1,\;2\right\}$.

For the remaining $\frac{pop}{2}$ individuals $\left(i=\left\lfloor \frac{pop}{2}\right\rfloor +1,\left\lfloor \frac{pop}{2}\right\rfloor +2,\cdots ,pop\right)$, the position of the iguana after falling to the ground is randomly determined. The movement strategy of ground individuals is described by Equation (3) [26]:

(3) $$X_{i,j}\left(t+1\right)=\left\{\begin{array}{c} X_{i,j}\left(t\right)+\left(X_{best,j}\left(t\right)-I\cdot X_{i,j}\left(t\right)\right)\cdot rand\left(0,\;1\right),\;\;\;if\;f_{{iguana}^{G}}<f_{i}\\ X_{i,j}\left(t\right)+\left(X_{i,j}\left(t\right)-X_{best,j}\left(t\right)\right)\cdot rand\left(0,\;1\right),\;\;\;\;\;\;\;\;\;\;\;\;\;\;if\;f_{{iguana}^{G}}\geq f_{i} \end{array}\right.$$

where $f_{i}$ represents the fitness function value of the $i^{th}$ individual, the ground position of the iguana is randomly generated as

(4) $${iguana}_{j}^{G}=\left({ub}_{j}-{lb}_{j}\right)\cdot rand\left(0,\;1\right)+{lb}_{j}$$

where $f_{{iguana}^{G}}\;$ denotes the objective function value at the ground position of the iguana.

### 2.3. Exploitation

Based on the natural behavior of coatis escaping from predators, COA incorporates a local search mechanism. When threatened by predators, a coati moves from its current position to a nearby, safer region. This behavior is abstracted into the algorithm’s local exploitation strategy, allowing the population to perform fine-grained searches around the current best solution. This phase is described by the following equations [26]:

The local lower and upper bounds for the $j^{th}$ decision variable are defined as

(5) lbjlocal=lbjtubjlocal=ubjtt=1, 2, ⋯, T

and the position of the $j^{th}$ individual is updated as

(6) $$X_{i,j}\left(t+1\right)=X_{i,j}\left(t\right)+\left(1-2r\right)\cdot \left({lb}_{j}^{local}\left(t\right)+rand\left(0,\;1\right)\left({ub}_{j}^{local}\left(t\right)-{lb}_{j}^{local}\left(t\right)\right)\right)$$

where ${lb}_{j}^{local}$ and ${ub}_{j}^{local}$ are the local lower and upper bounds of the $j^{th}$ decision variable, and r is a random number in the interval [0, 1].

Subsequently, the algorithm updates the position based on an effective update condition, modeled by:

(7) $$X_{nwe}\left(t+1\right)=\left\{\begin{array}{c} X_{i,j}\left(t+1\right),\;\;\;if\;f_{i}(t+1)<f_{i}(t)\\ X_{i,j}\left(t\right),\;\;\;\;\;\;\;\;\;\;\;\;\;\;\;\;\;if\;f_{i}(t+1)\geq f_{i}(t) \end{array}\right.$$

where denotes the objective function value of the $i^{th}$ individual at iteration t $f_{i}$ represents the fitness function value of the $i^{th}$ individual.

The pseudocode of the COA is outlined in Algorithm 1.
**Algorithm 1:** the pseudo-code of the COA1: ***Begin***
2: **Initialize:** the relevant parameters iterations T and the number of coatis pop. 3: Calculate the fitness of the objective function.
4:   For t <T **do**
5:        **Exploration:**
6:        $\mathrm{For}\;i=1:\left\lfloor pop/2\right\rfloor$ ***do***
7:            $\mathrm{Calculate}\;\mathrm{new}\;\mathrm{position}\;\mathrm{for}\;\mathrm{the}\;i^{th}$ coati using Equation (2).
8:        ***End for***
9:        $\mathrm{For}\;i=1+\left\lfloor pop/2\right\rfloor :pop$
***do***
10:          $\mathrm{Calculate}\;\mathrm{new}\;\mathrm{position}\;\mathrm{for}\;\mathrm{the}\;i^{th}$ coati using Equations (3) and (4).
11:       ***End for***
12:       **Exploitation:**
13:       Fori=1:pop
***do***
14:          $\mathrm{Calculate}\;\mathrm{new}\;\mathrm{position}\;\mathrm{for}\;\mathrm{the}\;i^{th}$ coati using Equations (5) and (6).
15:       ***End for***
*16:*       t=t+1
*17:*    ***End for***
18:    ***return*** best solution
19: ***end***

## 3. Proposed MECOA

### 3.1. Elite-Guided Search Strategy

In the standard COA, the hunting phase only guides individuals toward the global best solution through simple linear random perturbations. This mechanism has clear limitations: the step sizes of linear perturbations are small and uniformly distributed, making it difficult to escape local optima in complex multimodal functions. Moreover, over-reliance on a single global best solution as the guiding target quickly reduces population diversity and significantly increases the risk of premature convergence. Such a simple exploration mechanism cannot sufficiently cover the search space, severely constraining the algorithm’s global search capability [31,32].

To address these issues, this paper proposes an elite-pool Lévy mutation strategy. This strategy establishes an elite pool composed of the top three individuals by fitness, replacing the single global best guidance mechanism. Specifically, the mean of the elite individuals is used as the guiding target with a probability of 10% to enhance search stability, while a single elite individual is randomly selected as the guiding target with a probability of 90% to maintain diversity. On this basis, a Lévy-distributed random perturbation is introduced. Leveraging the heavy-tailed property of Lévy flights, the algorithm combines occasional large-step jumps with fine-grained local searches. The core update formula is as follows:

(8) $$X_{i,j}\left(t+1\right)={Elite}_{j}(t)+L(\beta )\cdot ({Elite}_{j}(t)-X_{i,j}(t))$$

where the elite target Elite is generated as

(9) $$Elite=\left\{\begin{array}{c} \frac{1}{3}\sum_{k=1}^{3}X_{k},\;\;\;\;\;\;\;\;\;\;\;if\;rand<0.1\\ X_{k},k\sim U\left\{1,\;2,\;3\right\},\;\;\;\;\;\;\;\;\;otherwise \end{array}\right.$$

and the Lévy flight term L(β) is implemented using the Mantegna algorithm:

(10) $$L(\beta )=0.5\times \frac{\phi \cdot \Gamma (1+\beta )\cdot sin(\pi \beta /2)}{|v{|}^{1/\beta }},\phi ,v∼N(0,\;1)$$

where $X_{k},k=1,\;2,\;3$ represent the best, second-best, and third-best individuals in the current population, and rand is a random number in [0, 1], β is a constant fixed at 1.5.

**Figure 1:**
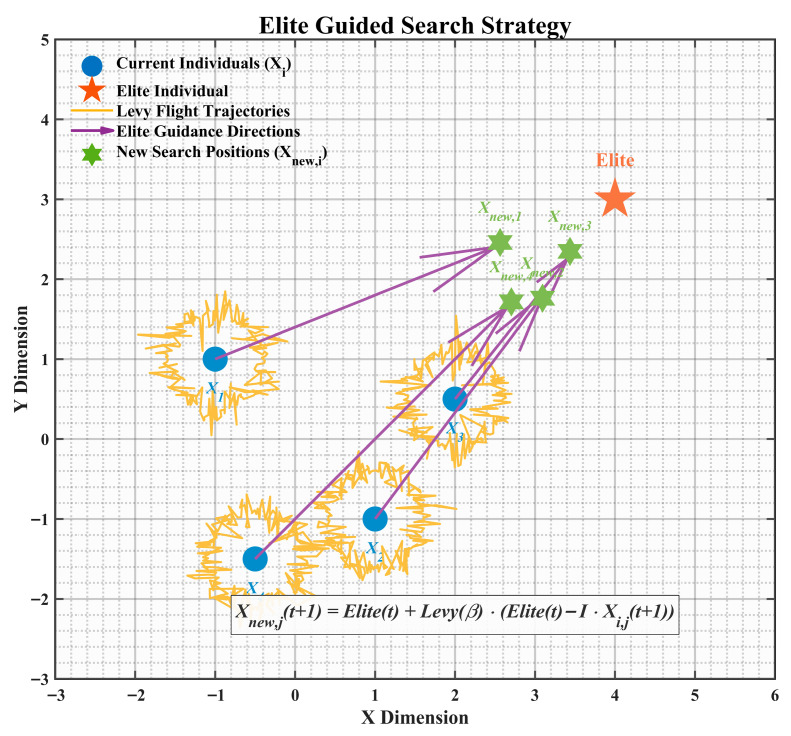
Schematic diagram of the elite-guided search strategy.

As illustrated in Figure 1, this strategy effectively avoids the over-attraction to a single best solution through multiple guiding targets. The heavy-tailed property of Lévy flights enables large jumps in the early stages to enhance global exploration, while fine guidance by elite individuals improves local exploitation in later stages, significantly boosting the algorithm’s optimization performance in complex multimodal functions.

### 3.2. Horizontal Crossover Strategy

The standard COA lacks an information-sharing mechanism among individuals, with each individual performing independent searches, resulting in low utilization of population information [33]. This isolated search pattern makes it difficult to form a cooperative effect and fails to effectively leverage the characteristics of multiple high-quality individuals in the population, limiting both convergence speed and accuracy.

**Figure 2:**
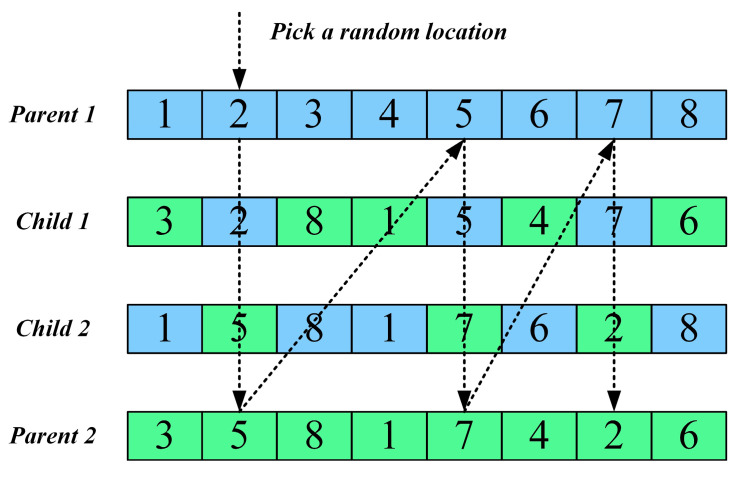
Schematic diagram of horizontal crossover strategy.

To enhance information exchange and cooperative search capability among individuals, this paper introduces a horizontal crossover mechanism. As illustrated in Figure 2, this strategy simulates the gene recombination process in biology, where individuals in the population are randomly paired, and linear combinations along with difference vector perturbations are applied to facilitate thorough information exchange. The mechanism is expressed as follows:

(11) Offspring1=r1⋅Parent1+(1−r1)⋅Parent2+c1⋅(Parent1−Parent2)Offspring2=r2⋅Parent2+(1−r2)⋅Parent1+c2⋅(Parent2−Parent1)

where $r_{1},r_{2}\;are\;random\;vectors\;in\;the\;range$ [0, 1], and $c_{1},c_{2}$ are random perturbation coefficients in the range [−1, 1].

This strategy effectively promotes the propagation of high-quality genes and maintains population diversity through information recombination among individuals. The difference vector perturbation introduces new search directions into the optimization process, helping to avoid premature convergence. After generating the offspring, boundary control is applied, and a greedy selection mechanism is used to update the parent population, ensuring continuous improvement of population quality.

### 3.3. Precision Elimination Mechanism

The standard COA uses a fixed population size throughout the optimization process and lacks an effective population management mechanism. The persistent presence of low-fitness individuals not only wastes computational resources but may also guide the population toward poor regions, reducing convergence efficiency [34].

To improve overall population quality and accelerate convergence, this paper proposes a fitness-based precise elimination mechanism. At the end of each generation, the population is sorted according to fitness, and the worst 20% of individuals are eliminated. New individuals are then randomly generated within the search space to replace the eliminated ones.

The number of individuals to be eliminated is calculated as:

(12) $$N_{eliminate}=round\left(pop\times elimination\_rate\right)$$

where pop is the population size and round(·) denotes the rounding function. The $N_{eliminate}$ individuals with the lowest fitness are selected for elimination.

**Figure 3:**
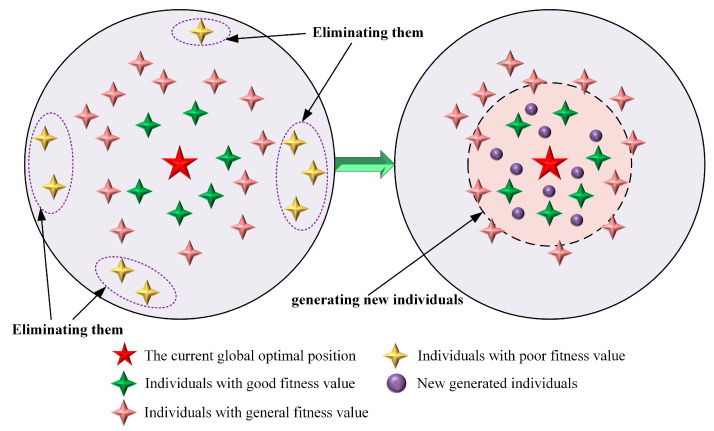
Schematic diagram of a precision elimination mechanism.

To avoid ineffective search due to purely random generation, the new individuals are generated in the neighborhood of the current global best solution $X_{best}$, according to:

(13) $$X_{\mathrm{new}\;,k}=X_{best}+\delta (t)\cdot \left(rand\;(1,dim)\cdot (ub-lb)-\frac{ub-lb}{2}\right)$$

where $\delta \left(t\right)=1-t/T$ is a decay factor that gradually decreases over iterations to control the distance of new solutions from the global best, rand (1,d) is a random vector of dimension d, and ub and lb are the upper and lower bounds of the search space, respectively. This mechanism ensures that the newly generated individuals explore the vicinity of the global best solution while progressively narrowing the search range over iterations, balancing exploration and convergence. A schematic of the precise elimination and generation mechanism is shown in Figure 3.

The MECOA’s pseudocode is provided in Algorithm 2.
**Algorithm 2:** the pseudo-code of the MECOA1: ***Begin***
2: **Initialize:** the relevant parameters iterations T and the number of coatis pop. 3: Calculate the fitness of the objective function.
4:   For t <T **do**
5:        **Exploration:**
6:        $\mathrm{For}\;i=1:\left\lfloor pop/2\right\rfloor$ ***do***
7:            $\mathrm{Calculate}\;\mathrm{new}\;\mathrm{position}\;\mathrm{for}\;\mathrm{the}\;i^{th}$ coati using Equations (8)–(10).
8:        ***End for***
9:        $\mathrm{For}\;i=1+\left\lfloor pop/2\right\rfloor :pop$
***do***
10:          $\mathrm{Calculate}\;\mathrm{new}\;\mathrm{position}\;\mathrm{for}\;\mathrm{the}\;i^{th}$ coati using Equations (3) and (4).
11:       ***End for***
12:       **Exploitation:**
13:       For i=1:pop
***do***
*14:*          $\mathrm{Calculate}\;\mathrm{new}\;\mathrm{position}\;\mathrm{for}\;\mathrm{the}\;i^{th}$ coati using Equations (5) and (6).
15:         ***End for***
*16:*       $\mathrm{Calculate}\;\mathrm{new}\;\mathrm{position}\;\mathrm{for}\;\mathrm{the}\;i^{th}$ coati using Equation (11).
*17:*       Eliminate and generate new individuals by Equations (12) and (13)
*18:*       t=t+1
*19:*   ***End for***
20:   ***return*** best solution
21: ***end***

### 3.4. Computational Time Complexity

Algorithm performance is critical, but evaluating its time complexity is equally important. In many optimization tasks, an algorithm must not only deliver excellent performance but also demonstrate satisfactory real-time efficiency. Time complexity reflects how the algorithm’s running time scales with the input size, providing insight into its computational cost for large-scale problems. In COA, the computational complexity for evaluating the defined control parameters is O(N×dim), where N denotes the population size and dim represents the problem dimension. During the initialization phase, the algorithm incurs a time cost of O(N×dim). Furthermore, after T iterations, updating the positions of all individuals has a computational complexity of O(T×N×dim). Therefore, the overall computational complexity of COA can be expressed as O(T×N×dim). In MECOA, while most modified strategies (such as the hybrid boundary repair mechanism, elite guidance, and crossover operations) do not introduce additional complexity factors beyond O(N×dim) per iteration, the precise elimination mechanism explicitly requires sorting the population by fitness in each generation. A sorting operation on N individuals has a time complexity of $O(Nlog\left(N\right))$. Therefore, the computational complexity per iteration of the Improved COA becomes $O(N\times Dim+Nlog\left(N\right))$. Over T iterations, the total time complexity is updated to $O(T\times \left(N\times Dim+Nlog\left(N\right)\right))$.

## 4. Experimental Results and Detailed Analyses

In this section, the proposed MECOA is experimentally evaluated using the CEC 2017 [35] and CEC 2022 [36] benchmark test suites. First, the parameter settings of all comparative algorithms are detailed, followed by a qualitative analysis of the MECOA. Additionally, MECOA is compared with seven other algorithms on the CEC 2017 and CEC 2022 test suites.

To ensure a fair comparison and reduce the influence of randomness, all algorithms are executed with a fixed population size of 30 and a maximum of 500 iterations. Each algorithm is independently run 30 times. The results are evaluated in terms of average value (Ave) and standard deviation (Std), with the best performance highlighted in bold. All experiments are conducted on a Windows 11 operating system with a 13th Gen Intel(R) Core(TM) i5-13400 CPU @ 2.5 GHz and 16 GB RAM, using MATLAB 2024b.

### 4.1. Competitor Algorithms and Parameters Setting

In this section, the superior performance of the proposed MECOA is validated through comparative experiments with eight state-of-the-art algorithms, including the Harris Hawks Optimization (HHO), Improving the search performance of SHADE using linear population size reduction (LSHADE), Gold Rush Optimizer (GRO), Grey Wolf Optimizer (GWO), Whale Optimization Algorithm (WOA), Particle Swarm Optimization (PSO), Holistic Swarm Optimization (HSO), Dung Beetle Optimizer (DBO), and the standard Coati Optimization Algorithm (COA).

For the comparative experiments, all algorithm parameters are set according to their respective references. For ease of reference, Table 1 summarizes the parameter configurations of all algorithms and provides the corresponding references for further study.

### 4.2. Qualitative Analysis of MECOA

In this subsection, we conduct a qualitative analysis of the proposed MECOA. Initially, we examine the diversity of the algorithm’s population, which plays a crucial role in exploring the unknown space effectively. Next, we evaluate the balance between exploration and exploitation, as the initial iterations require stronger exploration, while later iterations focus more on exploitation. We validate the performance of MECOA through experiments that measure both exploration and exploitation. Lastly, to assess the effectiveness of the improvements made, we perform ablation experiments. Detailed explanations are provided below.

#### 4.2.1. Analysis of the Population Diversity

In optimization algorithms, population diversity describes the degree of variation among individuals in a population [31,45], where each individual typically corresponds to a candidate solution. A decline in population diversity can lead to premature convergence to a local optimum, thereby constraining the algorithm’s global search capability. On the other hand, preserving higher diversity facilitates exploration across broader areas of the solution space and improves the probability of locating the global optimum. In this subsection, we evaluate the population diversity of the MECOA based on Equation (14) [46,47]

(14) $$I_{C}\left(t\right)=\sqrt{\sum_{i=1}^{N}\;\sum_{d=1}^{D}\;{\left(x_{id}\left(t\right)-c_{d}\left(t\right)\right)}^{2}},$$

where $I_{C}\left(t\right)$ denotes the population diversity, N represents the population size, D indicates the problem’s dimensionality, and $x_{id}\left(t\right)$ denotes the value of the i individual in the d dimension at the t iteration. $c_{d}\left(t\right)$ quantifies the dispersion degree of the entire population relative to its center of mass at iteration t, which is calculated using Equation (15).

(15) $$c_{d}\left(t\right)=\frac{1}{D}\sum_{i=1}^{N}\;x_{id}\left(t\right).$$

**Figure 4:**
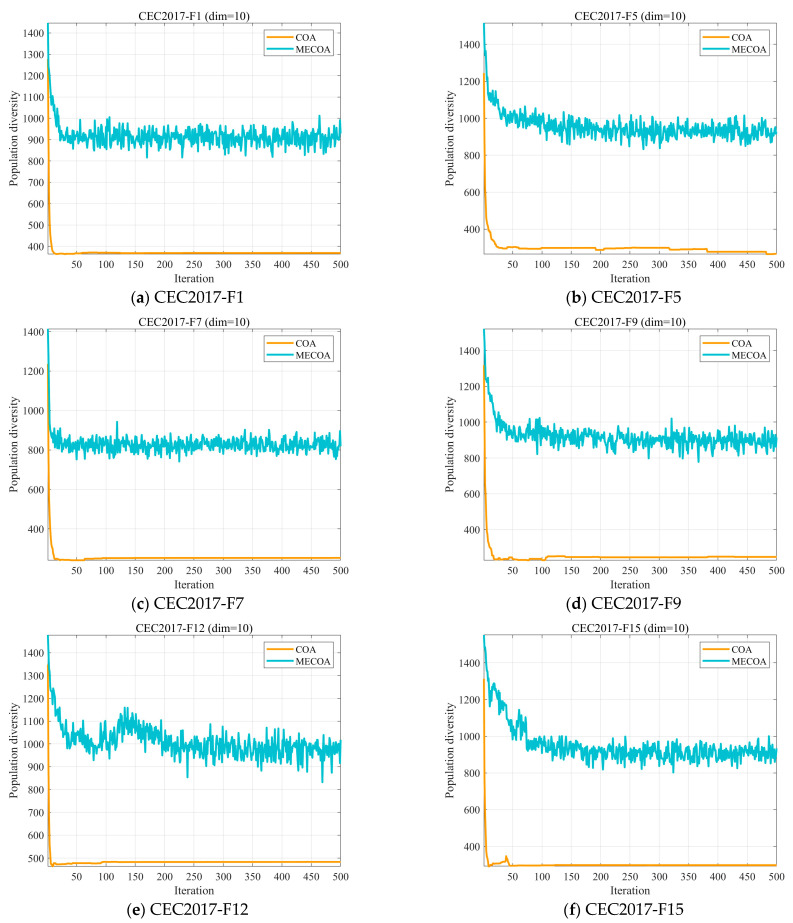

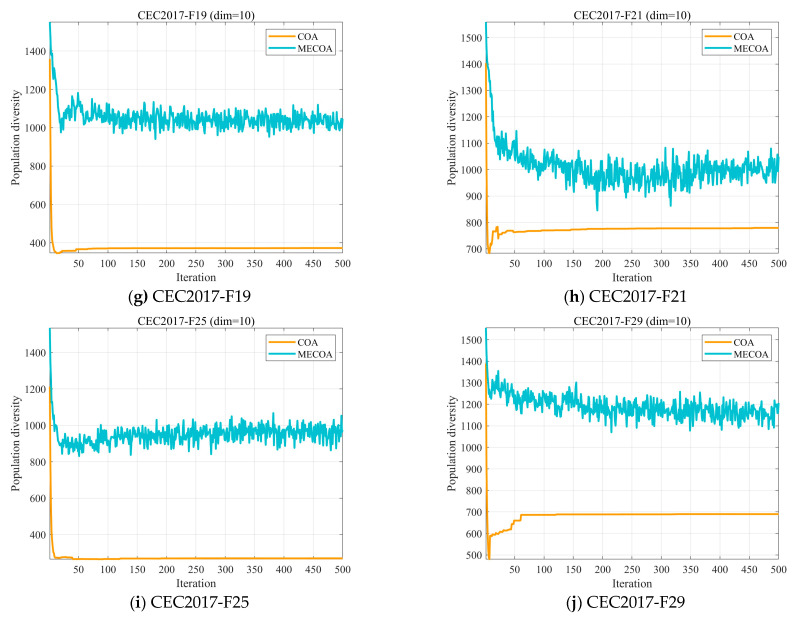
The analysis of the population diversity of MECOA and COA.

Figure 4 presents the experimental outcomes of the population diversity analysis for both algorithms. It is evident that in most cases, the MECOA exhibits higher population diversity than the COA. While the population diversity of COA typically declines to a very low level within 50 generations, MECOA demonstrates a more gradual reduction in diversity and is better able to preserve population diversity throughout the process. This discrepancy stems from the combined effect of the three major enhancement strategies integrated into MECOA, enabling it to effectively balance “diversity preservation” and “convergence efficiency” throughout the entire iterative cycle. This provides critical support for MECOA to break through local optima and search for global optimal solutions in complex multimodal functions (such as F9, F15, and other test functions containing multiple local extrema).

#### 4.2.2. Analysis of the Exploration and Exploitation

In optimization algorithms, both exploration and exploitation are essential components. Exploration refers to the algorithm’s ability to conduct a wide-ranging search across the solution space, seeking out diverse and potentially undiscovered regions that might contain the global optimum. Exploitation, conversely, entails an intensive search within the vicinity of known high-quality solutions, refining them to achieve greater accuracy. This process utilizes existing information to deepen search efforts in promising areas.

Excessive exploration may lead to inefficient use of computational resources by scanning the solution space broadly without focused improvement, thereby overlooking opportunities to enhance solutions in specific regions. On the other hand, over-emphasis on exploitation can result in premature convergence to a local optimum, hindering the algorithm’s ability to locate superior solutions in other parts of the search space [48,49]. Therefore, striking a balance between exploration and exploitation is critical to the overall effectiveness of the algorithm. In this subsection, we analyze the exploration and exploitation characteristics of the MECOA, which are quantitatively assessed using Equations (16) and (17) [47].

(16) $$Exploration\left(\%\right)=\frac{Div\left(t\right)}{Div_{max}}\times 100\%,$$

(17) $$Exploitation\left(\%\right)=\frac{\left|Div\left(t\right)-Div_{max}\right|}{Div_{max}}\times 100\%,$$

where $Div\left(t\right)$ denotes the measure of diversity at the tth iteration, which is calculated by Equation (18), and $Div_{max}$ denotes the maximum measure of diversity throughout the iteration.

(18) $$Div\left(t\right)=\frac{1}{D}\sum_{d=1}^{D}\frac{1}{N}\sum_{i=1}^{N}|median\left(x_{d}\left(t\right)\right)-x_{id}\left(t\right)|.$$

**Figure 5:**
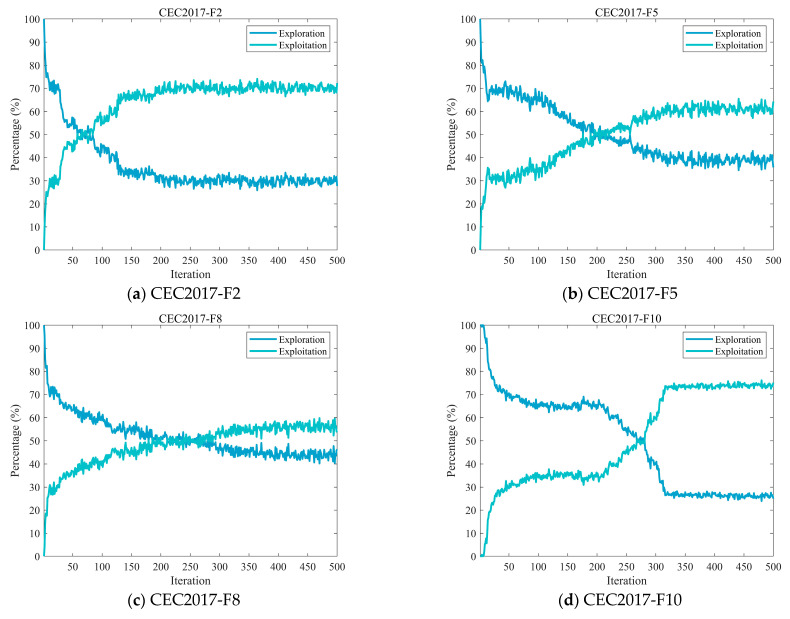

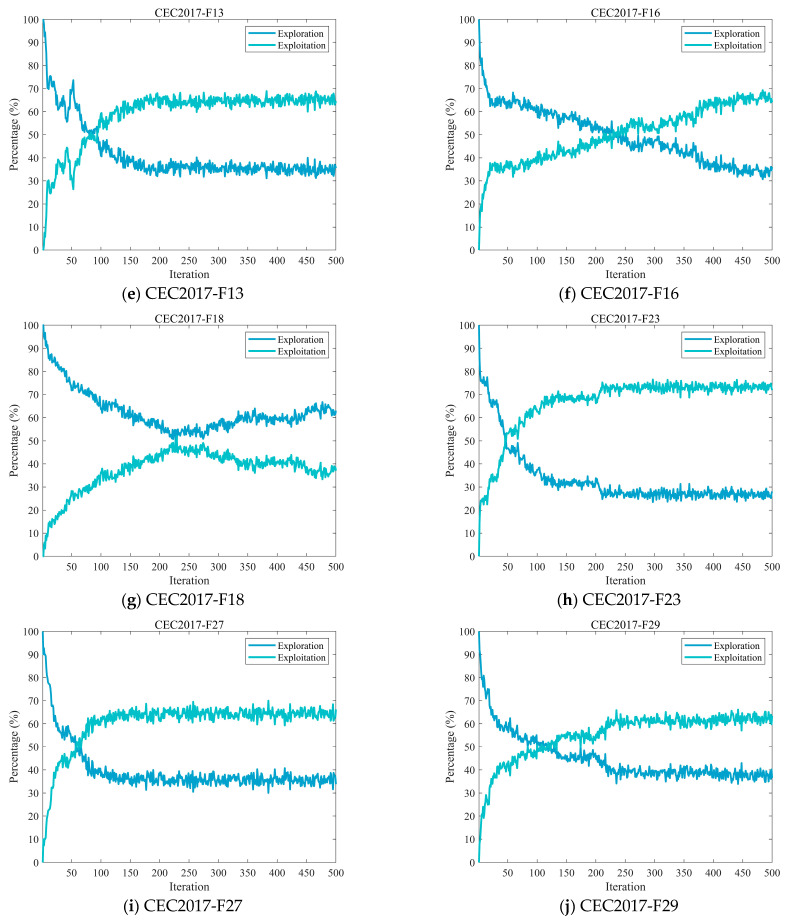
The analysis of the exploration and exploitation of MECOA.

Figure 5 illustrates the dynamic proportion changes between exploration and exploitation behaviors of the MECOA during the iterative process. The exploration rate, calculated as the ratio of population diversity to maximum diversity, reflects the algorithm’s ability to search unknown regions of the solution space. The exploitation rate, measured by the proportional difference between the two, indicates the algorithm’s effectiveness in conducting refined searches near known high-quality solutions. As shown by the curve trends, MECOA exhibits a significantly higher exploration rate (generally exceeding 60%) in the early stages of iteration (approximately the first 100 generations), enabling extensive traversal of the solution space to identify potential optimal regions. As the iteration progresses, the exploration rate gradually decreases, while the exploitation rate correspondingly increases. The varying positions of the intersection points between the exploration and exploitation curves indicate when the two behaviors begin to approach equilibrium, after which the algorithm progressively shifts its focus toward exploitation. By the later stages of iteration (after 400 generations), the exploitation rate approaches 70%, allowing for precise searches near the current optimal solution to enhance solution accuracy.

This dynamic balance of “exploration first, exploitation later” effectively avoids issues such as computational resource waste and difficulty in convergence caused by overemphasis on exploration, as well as premature convergence and entrapment in local optima resulting from excessive exploitation.

#### 4.2.3. Ablation Experiments

**Figure 6:**
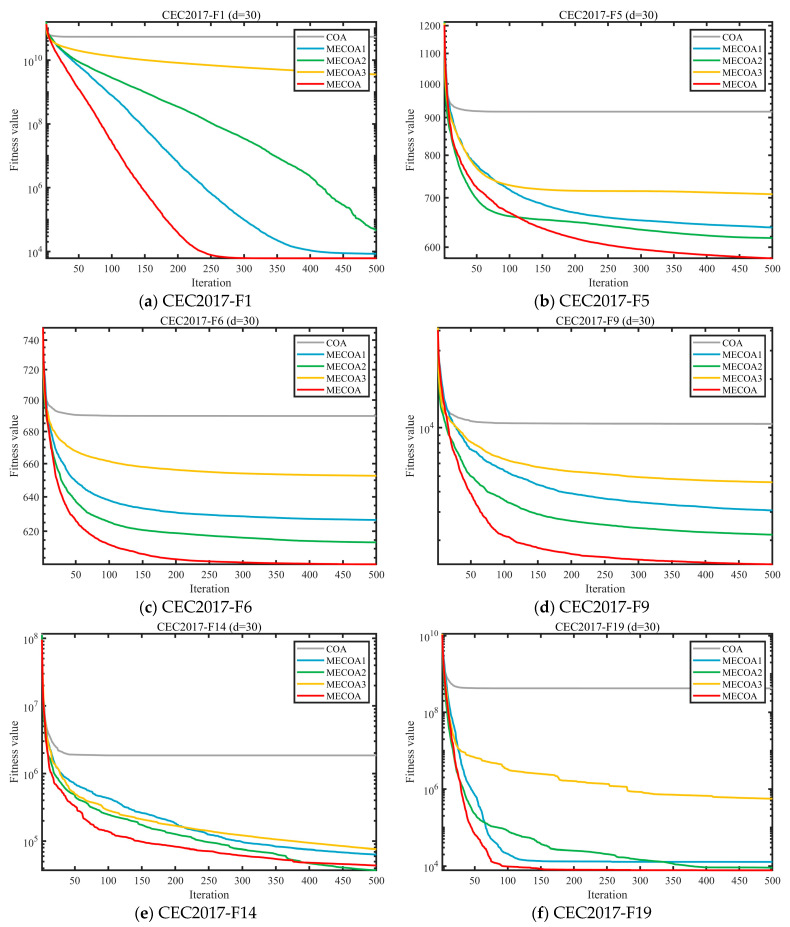

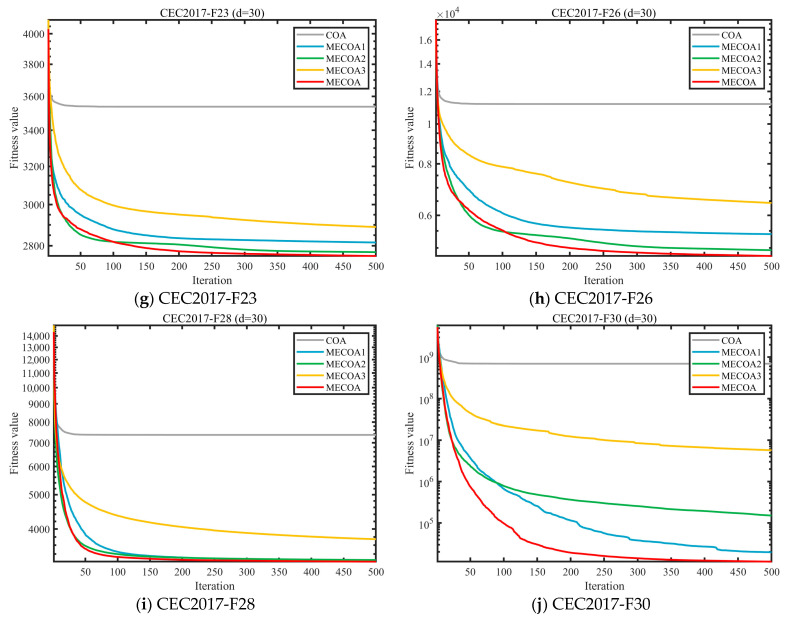
Comparison of different improvement strategies.

**Figure 7:**
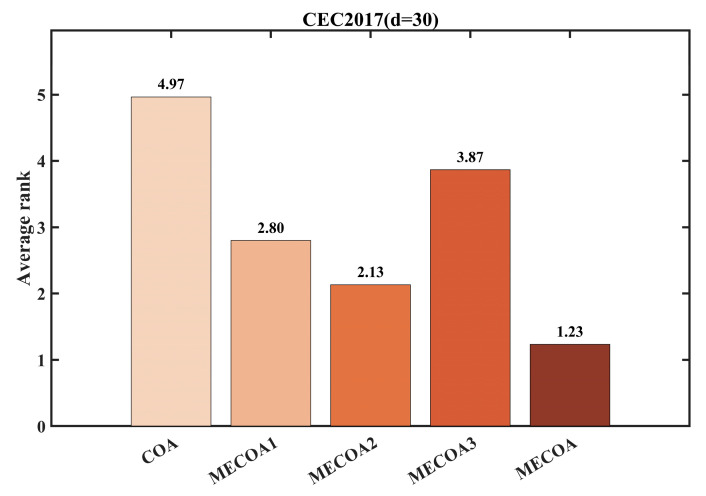
Average ranking of COA improved by different strategies.

To evaluate the independent contributions and synergistic effects of the three enhanced strategies—Elite-Guided Search Strategy (S1), Horizontal Crossover Strategy (S2), and Precision Elimination Mechanism (S3)—an ablation study was conducted using the CEC 2017 benchmark with a problem dimension of d = 30. Four comparative variants were designed: MECOA1 (containing only S1), MECOA2 (containing only S2), MECOA3 (containing only S3), and the fully integrated MECOA incorporating all strategies. The experimental results are presented in Figure 6 and Figure 7.

Figure 6 presents the convergence curves of multiple representative test functions (F1, F5, F6, F9, etc.) from the CEC2017 benchmark (30 dimensions), comparing the original COA with the MECOA variants incorporating individual strategies—MECOA1 (only Elite-Guided Search Strategy S1), MECOA2 (only Horizontal Crossover Strategy S2), MECOA3 (only Precision Elimination Mechanism S3)—as well as the fully integrated MECOA. Figure 7 quantifies the improvements of each strategy on COA in terms of average rankings.

From the convergence curves in Figure 6, it can be seen that the original COA exhibits slow convergence and a tendency to become trapped in local optima for most test functions (e.g., the fitness value of COA remains above 10^9^ for F1 and struggles to break 10^4^ for F9). In contrast, the fitness values of MECOA1, MECOA2, and MECOA3 decrease more rapidly and reach lower final convergence values than COA, validating the independent contributions of the three strategies—S1 enhances global exploration through elite guidance and Lévy flights, S2 improves convergence efficiency via information recombination among individuals, and S3 optimizes population quality through selective elimination.

More importantly, the fully integrated MECOA achieves the best performance across all test functions, showing the fastest decline in fitness values (e.g., for F1, the fitness drops to the 10^3^ level after 500 iterations; for F23, it rapidly decreases from 4000 to around 2700) and the lowest final convergence values, fully demonstrating the synergistic effect of the three strategies. The average ranking data in Figure 7 further support this conclusion: the original COA has the lowest average ranking (4.97), MECOA1 (3.87), MECOA2 (2.80), and MECOA3 (2.13) progressively improve, while the fully integrated MECOA ranks first with an average ranking of 1.23. This clearly indicates that although single-strategy improvements enhance COA performance, the multi-strategy synergy maximizes optimization effectiveness, strongly supporting MECOA’s advantages in complex multimodal optimization problems.

### 4.3. Compare Using CEC 2017 and CEC2022 Test Functions

**Figure 8:**
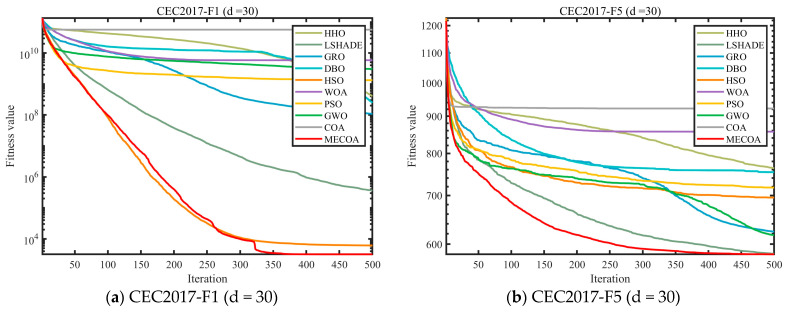

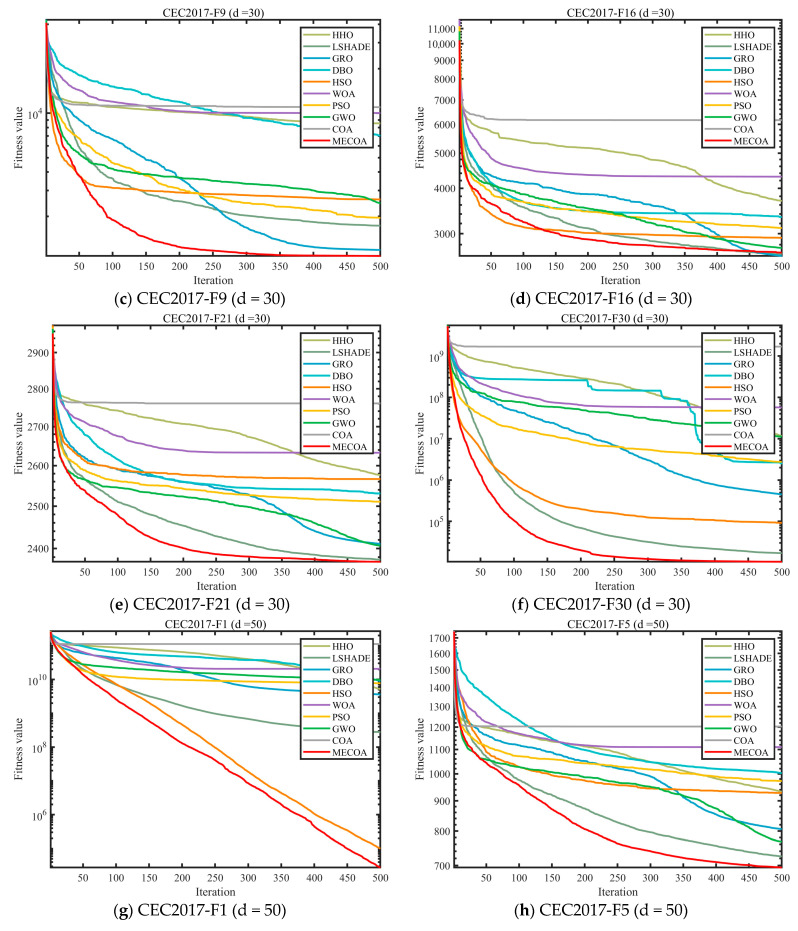

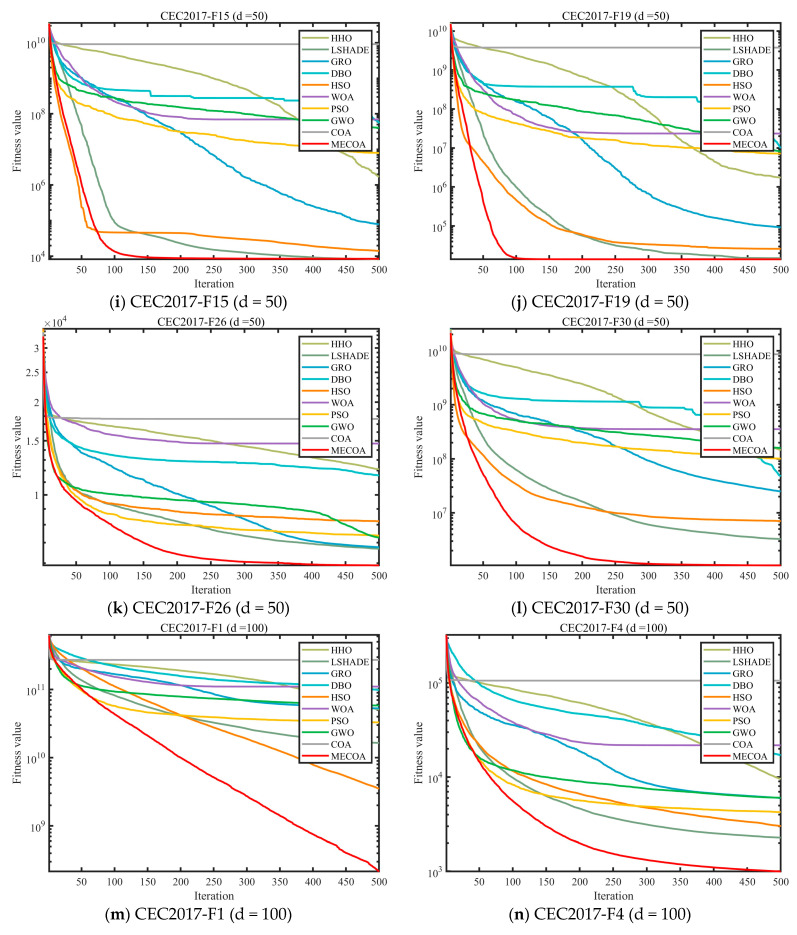

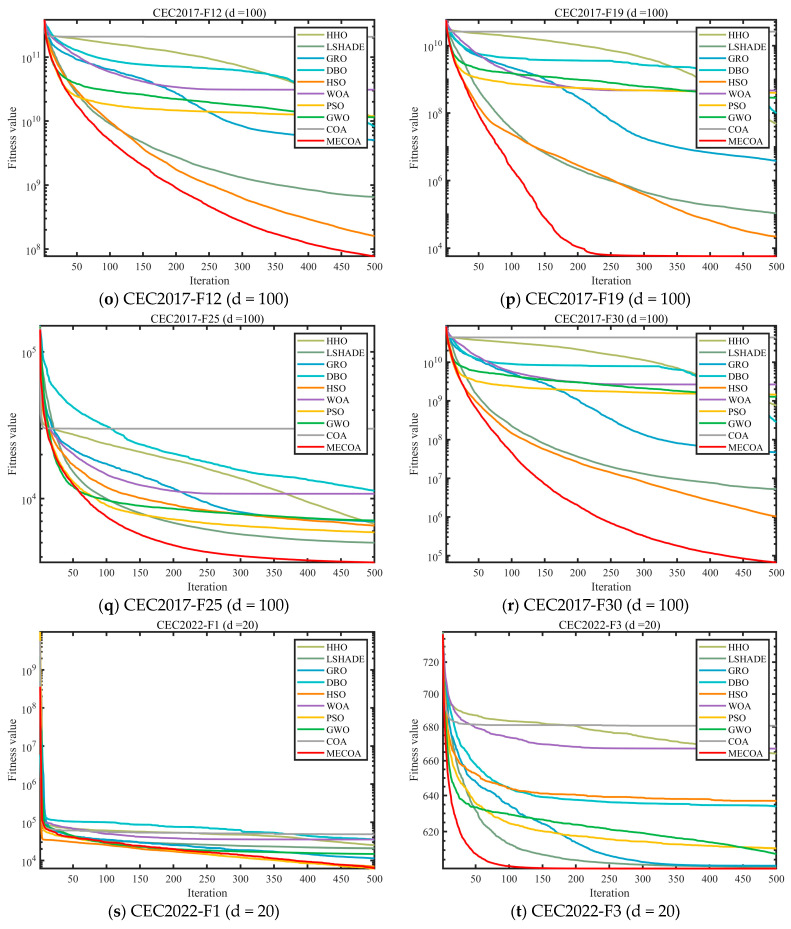

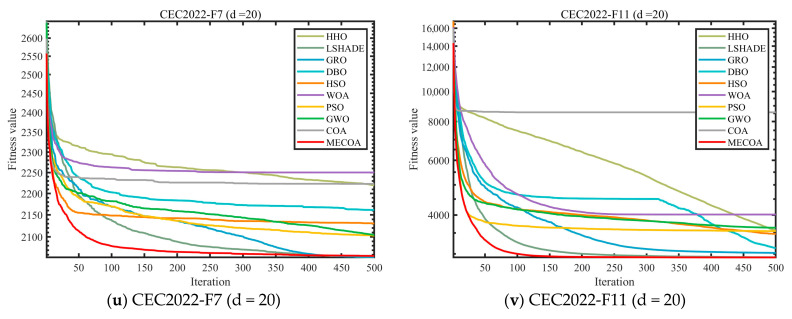
Comparison of convergence speed of different algorithms on CEC2017 and CEC2022 test set.

**Figure 9:**
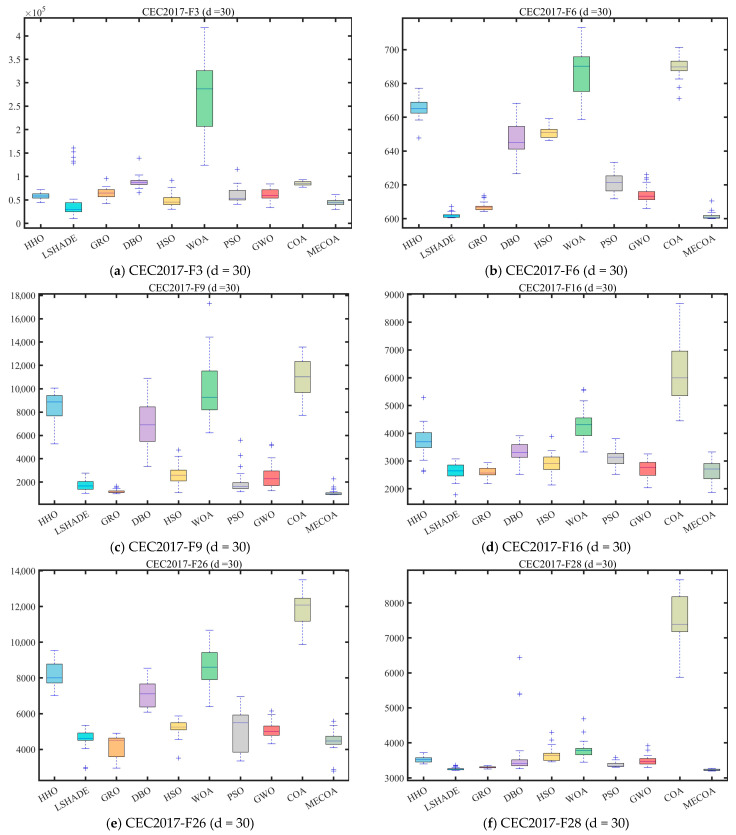

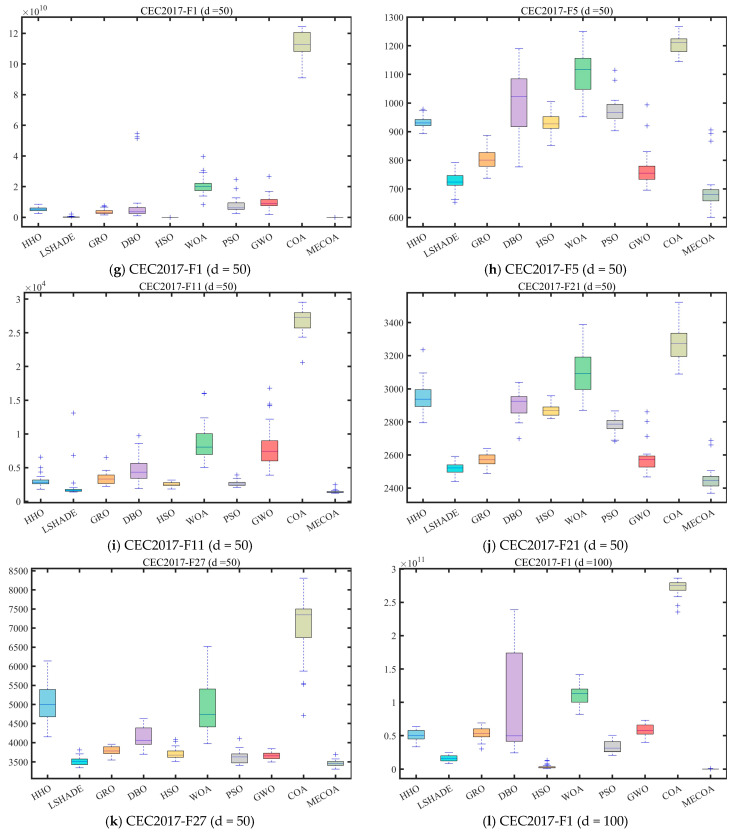

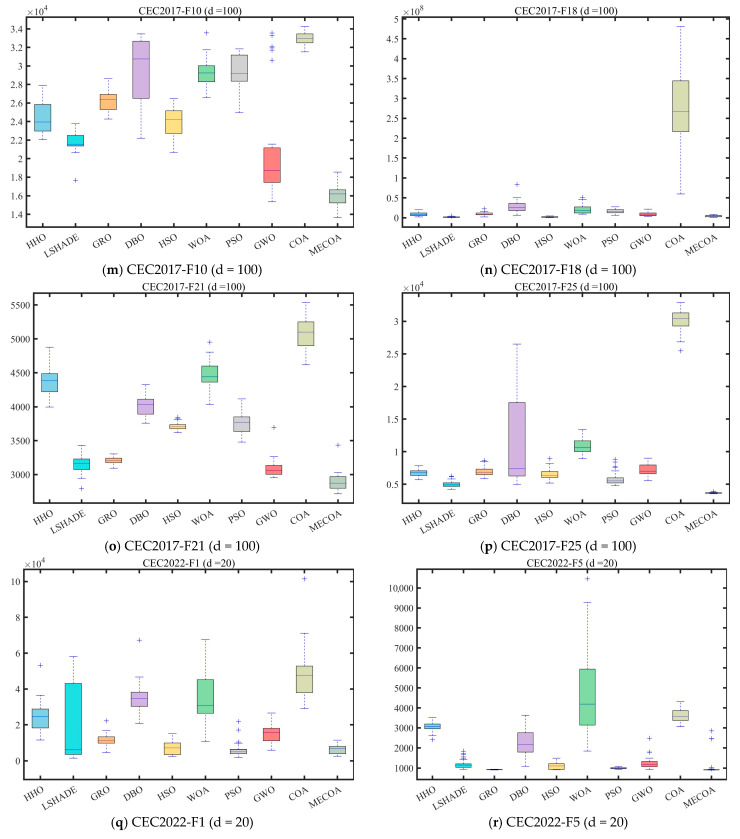

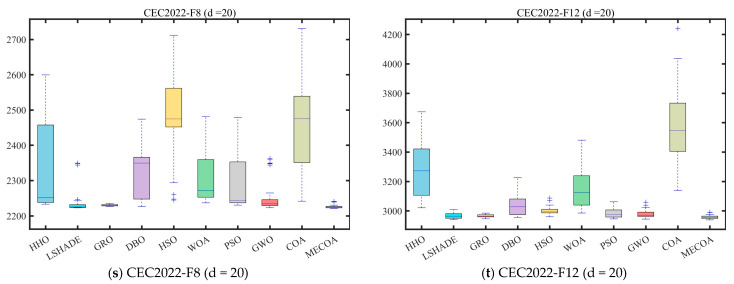
Boxplot analysis for different algorithms on the CEC2017 and CEC2022 test set.

In this subsection, experiments were conducted using the CEC 2017 and CEC 2022 benchmark sets to validate the effectiveness of the proposed MECOA. The MECOA was compared with seven state-of-the-art algorithms, clearly demonstrating both its advantages and limitations. The numerical results are presented in Table 2, Table 3, Table 4 and Table 5, and the bold font indicates the best values. To intuitively illustrate the convergence speed during the optimization process, Figure 8 shows selected convergence curves of all eight algorithms. To reduce the impact of randomness and further assess algorithm stability, Figure 9 presents partial box plots for all algorithms.

Table 2, Table 3, Table 4 and Table 5 systematically present the mean (Ave) and standard deviation (Std) results of MECOA and nine comparison algorithms (HHO, LSHADE, GRO, DBO, HSO, WOA, PSO, GWO, COA) on the CEC2017 (dim = 30/50/100) and CEC2022 (dim = 20) benchmark test sets, covering typical optimization functions such as unimodal, multimodal, and composite functions, providing quantitative support for the optimization accuracy and stability of MECOA. In the CEC2017 (dim = 30) tests, MECOA demonstrated particularly significant advantages on key functions: in the unimodal function F1, the Ave value of MECOA (4.4375 × 10^4^) was only 52% of the original COA (8.5224 × 10^4^), and the Std (5.0212 × 10^2^) was only 1/15 of COA (7.6606 × 10^3^), reflecting higher accuracy and stability; in the multimodal function F19, the Ave value of MECOA (8.2078 × 10^3^) was significantly lower than COA (7.4352 × 10^8^), with a difference of nine orders of magnitude, and the Std (8.8601 × 10^3^) was better than comparison algorithms such as LSHADE (9.8783 × 10^3^); in the composite function F30, the Ave value of MECOA (1.0534 × 10^4^) was only 1/1.6 × 10^5^ of COA (1.6934 × 10^9^), and the Std (3.7981 × 10^3^) was the lowest among all algorithms. When the dimension increased to 100, MECOA’s advantages remained undiminished: in the F1 function, the Ave value of MECOA (2.1586 × 10^8^) was only 1/1.26 × 10^3^ of COA (2.7212 × 10^11^); in the F30 function, the Ave value of MECOA (6.6470 × 10^4^) was 98.7% lower than LSHADE (5.1994 × 10^6^). In CEC2022 (dim = 20), MECOA also performed exceptionally well: in the F3 function, the Ave value of MECOA (6.0019 × 10^2^) was close to the theoretical optimum, and the Std (3.2379 × 10^−1^) was only 1/28 of COA (9.1194 × 10^0^); in the F10 function, the Ave value of MECOA (2.5096 × 10^3^) was close to LSHADE (2.5044 × 10^3^), but the Std (3.3854 × 10^1^) was lower, verifying its reliability on the new benchmark test set.

Figure 8 visualizes the iterative optimization process of MECOA and the comparison algorithms through convergence curves, further corroborating the performance advantages of MECOA shown in Table 2, Table 3, Table 4 and Table 5, particularly highlighting its convergence speed and ability to approach the global optimum. In the CEC2017 (dim = 30) benchmark, for the F1 function, the fitness value of MECOA drops to the order of 10^4^ after only 100 iterations, while COA remains around 10^7^, and algorithms such as GWO and PSO fluctuate within the range of 10^5^–10^6^, demonstrating MECOA’s superior capability for rapid early exploration. For the F9 function, after 500 iterations, the fitness value of MECOA (1.9810 × 10^3^) is 95% lower than that of COA (3.8158 × 10^4^), and its convergence curve is smooth and stable without oscillation. This improvement stems from the heavy-tailed Lévy flight mechanism integrated into the elite-guided search strategy, which allows for long jumps to expand the search range while enabling short, fine-grained movements to approach the optimal solution. When the dimensionality increases to 100, MECOA continues to maintain a distinct convergence advantage. For the F12 function, MECOA reaches a stable convergence phase after 300 iterations, achieving a final fitness value of 6.4281 × 10^6^, which is only 1/1.35 × 10^4^ of COA’s (8.6643 × 10^10^). In the CEC2022 (dim = 20) benchmark, for the F5 function, the convergence curve of MECOA remains consistently below those of other algorithms, and after 500 iterations, its average fitness value (1.1355 × 10^3^) is 68% lower than that of COA (3.5843 × 10^3^). These results demonstrate that MECOA effectively overcomes the premature convergence and slow convergence speed problems of the original COA in high-dimensional and complex optimization scenarios.

Figure 9 quantifies the distribution dispersion of each algorithm’s results through boxplots, providing additional evidence of MECOA’s superiority from the perspective of robustness. A more compact boxplot with fewer outliers indicates stronger consistency across multiple independent runs and, consequently, higher robustness. In the CEC2017 (dim = 30) benchmark, MECOA demonstrates significantly better boxplot characteristics than the comparison algorithms on key functions. For the F3 function, the result distribution range of MECOA (mean ± standard deviation: 8.3114 × 10^3^ ± 3.3681 × 10^3^) is only one-fifth of that of COA (5.6804 × 10^4^ ± 1.2667 × 10^4^), with no outliers, reflecting strong result concentration. For the F16 function, MECOA achieves the lowest median value (2.6631 × 10^3^) among all algorithms, and its interquartile range (IQR) is only one-third of AOO’s, further confirming the stability of its results. For the F23 function, the boxplot of MECOA shows no upper-limit outliers, whereas algorithms such as DBO and HSO exhibit numerous extreme values beyond reasonable bounds, validating MECOA’s resistance to outliers. In the CEC2022 (dim = 20) benchmark, MECOA continues to maintain its robustness advantage. For the F7 function, the IQR of MECOA’s boxplot (2.2978 × 10^1^) is only one-third of that of HSO (6.7863 × 10^1^). For the F9 function, MECOA’s result distribution is entirely concentrated near the theoretical optimum value (2.4808 × 10^3^), showing no noticeable dispersion—consistent with its extremely low standard deviation (Std = 1.0706 × 10^−3^) reported in Table 2.

In summary, the quantitative statistics in Table 2, Table 3, Table 4 and Table 5, the convergence process visualization in Figure 8, and the robustness analysis in Figure 9 together form a complete chain of evidence. Through the synergistic effect of its three core strategies—elite-guided search, horizontal crossover, and precise elimination—MECOA not only significantly outperforms the original COA and other comparative algorithms in terms of optimization accuracy, but also maintains fast, stable convergence and strong robustness across various scenarios, including low- and high-dimensional as well as unimodal and multimodal problems. This comprehensive performance advantage provides solid theoretical and empirical support for MECOA’s subsequent application to complex engineering optimization tasks, such as parameter identification of photovoltaic models.

### 4.4. Statistical Analysis

Statistical analysis plays a vital role in algorithm optimization, enabling researchers to evaluate and contrast the efficacy of various algorithmic approaches. This facilitates the selection of the most suitable method for specific research challenges. In this section, the performance of the MECOA is assessed using the Wilcoxon rank-sum test and the Friedman test, with detailed procedures and results presented accordingly.

#### 4.4.1. Wilcoxon Rank Sum Test

In this subsection, the Wilcoxon rank-sum test [50] is employed to assess whether significant differences exist in the performance of the MECOA, without relying on assumptions of normality. Compared to the traditional t-test, the Wilcoxon test offers greater flexibility, as it remains applicable to data with non-normal distributions or outliers. The test statistic W for the Wilcoxon rank-sum test is defined by Equation (19).

(19) $$W=\sum_{i=1}^{n_{1}}R\left(X_{i}\right),$$

where $R\left(X_{i}\right)$ denotes the rank of $X_{i}$ among all observations. The test statistic U is calculated by Equation (20).

(20) $$U=W-\frac{n_{1}\left(n_{1}+1\right)}{2},$$

For larger sample sizes, U is approximately normally distributed by Equations (21) and (22).

(21) $$\mu_{U}=\frac{n_{1}n_{2}}{2},$$

(22) $$\sigma_{U}=\sqrt{\frac{n_{1}n_{2}\left(n_{1}+n_{2}+1\right)}{12}},$$

and the standardized statistic Z is calculated by Equation (23).

(23) $$Z=\frac{U-\mu_{U}}{\sigma_{U}},$$

A *p*-value threshold of 0.05 was used to assess the statistical significance of the outcomes from each MECOA execution relative to alternative methods. The initial assumption, or null hypothesis (H_0_), posits that the performance of the two approaches is equivalent. Should the calculated *p*-value fall below 0.05, H_0_ is dismissed, supporting the presence of a meaningful performance gap; if not, H_0_ is upheld. The *p*-values are showed in Appendix A.

Table 6 quantifies the performance differences between MECOA and nine comparative algorithms (HHO, LSHADE, GRO, DBO, HSO, WOA, PSO, GWO, COA) across different test suites and dimensions using the Wilcoxon rank-sum test at a significance level of 0.05. This statistical analysis validates the effectiveness of MECOA’s improvement strategies. In the CEC2017 test suite (Dim = 30), MECOA demonstrates a pronounced performance advantage: it achieves a “30/0/0” dominance over the traditional COA, meaning that for all 30 benchmark functions, MECOA significantly outperforms COA, with no cases of comparable or inferior performance. This result directly confirms the targeted enhancements of COA through MECOA’s three core strategies—elite-guided search, horizontal crossover, and precise elimination. Specifically, the elite pool expands search directions, gene recombination improves information sharing efficiency, and the elimination of low-fitness individuals optimizes population quality. Together, these strategies effectively address COA’s inherent limitations of reliance on a single global optimum and rapid decay of population diversity. For classical algorithms such as GWO, WOA, and PSO, MECOA also maintains a statistical advantage of “29/1/0” or “30/0/0,” differing insignificantly from LSHADE on only one complex multimodal function (e.g., F15), further highlighting its precise balance between exploration and exploitation in high-dimensional scenarios.

In the CEC2022 test suite (Dim = 20), MECOA’s statistical superiority remains consistently stable across scenarios. Against recently proposed algorithms such as HSO and DBO, MECOA achieves a “19/1/0” result across 20 test functions, with only one hybrid-variable function (F8) showing no significant difference compared to GRO. For traditional algorithms such as WOA and PSO, MECOA maintains an all-out performance of “20/0/0,” with no functions exhibiting inferior results. Even when the dimensionality increases to 50 and 100 in the extended CEC2017 tests, MECOA’s statistical significance shows no noticeable decay: at Dim = 50, it retains a “30/0/0” advantage over COA and achieves “28/2/0” against GWO and WOA, with only two high-dimensional unimodal functions (F2, F4) showing comparable performance. At Dim = 100, MECOA still demonstrates “27/3/0” superiority over COA and HHO, with only three ultra-high-dimensional complex functions (F3, F7, F12) showing no significant differences. This stable statistical dominance across test suites and dimensions not only confirms that MECOA’s improvement strategies effectively tackle challenges posed by increasing search space complexity but also reduces performance fluctuations caused by algorithmic randomness, providing robust statistical support for its subsequent application in practical engineering problems, such as photovoltaic model parameter identification.

From a practical perspective of statistical testing, the results in Table 6 further reveal the synergistic value of MECOA’s improvement strategies. Elite-guided search leverages the heavy-tail characteristics of Lévy flights to dynamically balance global exploration and local exploitation; horizontal crossover facilitates the propagation of high-quality information within the population to prevent premature convergence; and the precise elimination mechanism maintains evolutionary vitality through population updates. The combined effect of these three strategies enables MECOA to maintain performance stability across diverse optimization scenarios. Compared to single-strategy improvements (e.g., CWOA with only chaotic mapping or IALO optimizing solely for population diversity), MECOA’s multi-strategy synergy not only enhances search accuracy and convergence speed but also statistically validates the reliability of its performance gains. This provides a comprehensive “performance improvement—statistical verification” research paradigm for the application of metaheuristic algorithms in complex engineering optimization.

#### 4.4.2. Friedman Mean Rank Test

In this subsection, the Friedman test [51] is used to determine the overall ranking of the MECOA relative to other methods. As a nonparametric approach, the Friedman test is suitable for comparing median performance differences across three or more matched groups. It is particularly well-suited for repeated measures or block designs, and is often employed as a robust alternative to ANOVA when the assumption of normality is violated. The Friedman test statistic is calculated according to Equation (24).

(24) $$Q=\frac{12}{nk\left(k+1\right)}\sum_{j=1}^{k}R_{j}^{2}-3n\left(k+1\right),$$

where n is the number of blocks, k is the number of groups, and $R_{j}$ is the rank sum for j-th group. When n and k are large, Q follows approximately a $\chi^{2}$ distribution with k−1 degrees of freedom.

Table 7 quantifies the global performance ranking of MECOA and nine comparative algorithms (HHO, LSHADE, GRO, DBO, HSO, WOA, PSO, GWO, COA) across the CEC2017 (30/50/100 dimensions) and CEC2022 (20 dimensions) test suites using the Friedman mean-rank test at a significance level of 0.05. The results show that MECOA consistently secures the top position with an absolute advantage across all dimensional scenarios. In the 30-dimensional scenario of the CEC2017 test suite, MECOA achieves a mean rank (M.R) of only 1.87 and a total rank (T.R) of 1, far surpassing the second-ranked LSHADE (M.R = 2.03, T.R = 2) and the traditional COA ranked tenth (M.R = 9.93, T.R = 10). This pronounced rank difference directly demonstrates the synergistic effectiveness of MECOA’s three core strategies—elite-guided search, horizontal crossover, and precise elimination. Specifically, the elite pool expands search directions, gene recombination enhances information sharing within the population, and low-fitness individual elimination optimizes population quality, collectively overcoming COA’s reliance on a single global optimum and rapid decay of population diversity.

When the dimensionality increases to 50, MECOA’s mean rank remains stable at 2.07 (T.R = 1), leading LSHADE (M.R = 2.43, T.R = 2) and GRO (M.R = 3.47, T.R = 3). Even as search space complexity rises, its performance shows no significant degradation. In the ultra-high-dimensional 100-dimensional scenario, MECOA’s mean rank further improves to 1.83 (T.R = 1), with an even more pronounced advantage over COA (M.R = 9.63, T.R = 10) and WOA (M.R = 8.77, T.R = 9), demonstrating that its improvement strategies maintain precise control over the exploration-exploitation balance in high-dimensional complex scenarios. In the 20-dimensional CEC2022 test suite, MECOA achieves a mean rank of 1.58 (T.R = 1), remaining the only algorithm with a mean rank below 2.0, whereas traditional algorithms such as WOA (M.R = 5.42, T.R = 5) and PSO (M.R = 8.50, T.R = 8) exhibit significantly higher mean ranks. This indicates that MECOA’s improvement strategies effectively adapt to diverse optimization tasks (unimodal, multimodal, hybrid functions) in the new benchmark suite, maintaining superior performance stability compared to the comparative algorithms.

**Figure 10:**
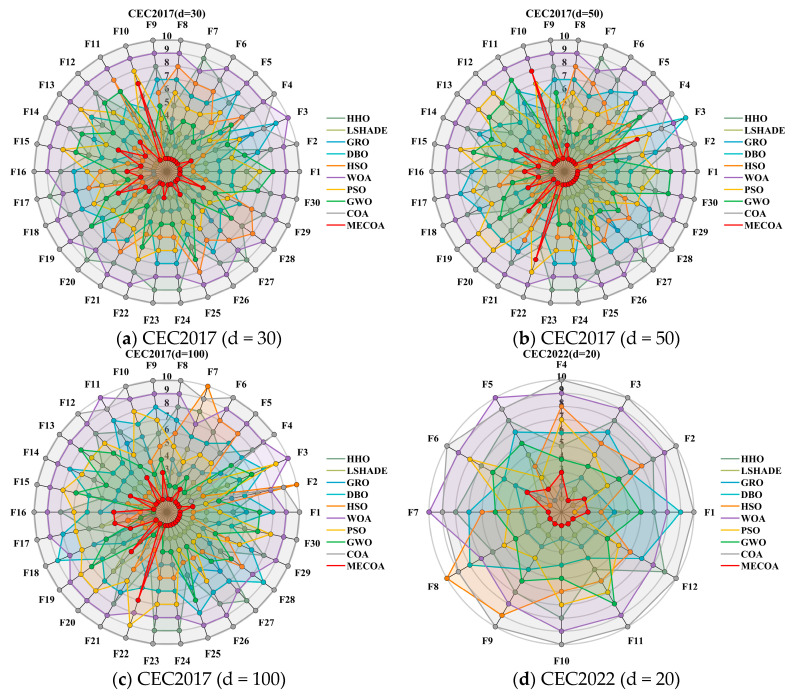
Distribution of rankings of different algorithms.

Figure 10 further visualizes the ranking distribution, intuitively illustrating the dispersion of performance across test functions and highlighting the stability and reliability of MECOA’s advantage. In the 30-dimensional CEC2017 scenario, MECOA exhibits a pronounced left-skewed ranking distribution, primarily concentrated in the 1st–2nd positions, with no function ranked below 3rd. This highly concentrated high-rank distribution indicates that MECOA consistently delivers optimal results across most optimization scenarios. In contrast, the traditional COA’s rankings are heavily right-skewed, mostly distributed in the 8th–10th positions, reflecting its inherent shortcomings of insufficient global exploration and proneness to local optima.

In the 50-dimensional CEC2017 scenario, MECOA maintains this left-skewed feature, with rankings still concentrated in the 1st–2nd positions and minimal dispersion. When the dimension increases to 100, MECOA’s rankings remain stably distributed in the 1st–2nd positions with very low dispersion, indicating that even in ultra-high-dimensional complex functions, its performance remains robust. In the 20-dimensional CEC2022 scenario, MECOA’s rankings are again primarily concentrated in the 1st–2nd positions, with no function lagging, further confirming its adaptability across different test suites. By contrast, other algorithms exhibit significantly higher dispersion in rankings: LSHADE ranks mainly in 2nd–3rd positions at 30 dimensions but disperses to 2nd–5th at 100 dimensions, showing clear instability; GWO ranks concentrated in 5th–6th at 30 dimensions but disperses to 6th–7th at 100 dimensions, indicating performance fluctuations with dimensional changes and difficulty in adapting to optimization tasks of varying complexity.

## 5. MECOA for Photovoltaic Models Parameter Estimation

### 5.1. Photovoltaic Model

Various mathematical models have been proposed in the literature to simulate the operating characteristics of photovoltaic (PV) cells and modules, among which the single-diode model (SDM) and double-diode model (DDM) are the most widely adopted. A brief introduction to these models is provided below.

#### 5.1.1. Single Diode Model (SDM)

**Figure 11:**
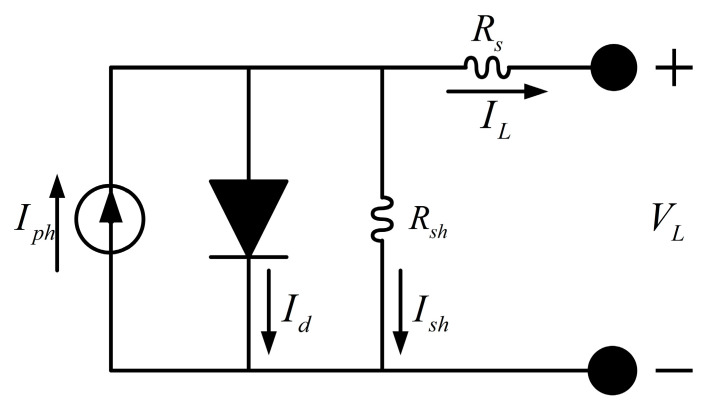
Equivalent circuit of the single diode model.

Due to its excellent balance between simplicity and accuracy, the single-diode model (SDM) is widely adopted by researchers to simulate the current–voltage $\left(I_{out}-V_{out}\right)$. characteristics of photovoltaic (PV) cells or modules. The equivalent circuit of the single-diode model is shown in Figure 11.

The single-diode model consists of a current source representing the photo-generated current from incident light, a diode simulating the PN junction of the semiconductor, a series resistor $R_{s}$ representing the resistive effects of electrodes, materials, and metal contacts, and a shunt resistor $R_{sh}$ accounting for leakage current through the semiconductor material.

The output current $I_{out}$ can be expressed as [52]:

(25) $$I_{out}=I_{ph}-I_{d}-I_{sh},$$

where $I_{ph},I_{d}$ and $I_{sh}$ denote the photo-generated current, the diode current, and the current through the shunt resistor, respectively. The diode and shunt currents are given by:

(26) $$I_{d}=I_{o}\cdot \left\{exp\left[\frac{q\cdot (V_{\mathrm{out}\;}+R_{s}\cdot I_{\mathrm{out}\;})}{a\cdot k\cdot T}\right]-1\right\},$$

(27) $$I_{\mathrm{sh}\;}=\frac{V_{\mathrm{out}\;}+R_{s}\cdot I_{\mathrm{out}\;}}{R_{sh}},$$

where $I_{o}$ is the diode reverse saturation current, a is the diode ideality factor, k is the Boltzmann constant $\left(1.3806503\times 10^{-23}\;J\cdot K^{-1}\right)$, q is the electron charge $\left(1.60217646\times 10^{-19}\;C\right)$, and T ambient temperature in Kelvin.

Substituting (26) and (27) into (25) yields the output current–voltage relationship of the single-diode model [10,13]:

(28) $$I_{out}=I_{ph}-I_{o}\cdot \left\{\exp\left[\frac{q\cdot \left(V_{\mathrm{out}\;}+R_{s}\cdot I_{\mathrm{out}\;}\right)}{a\cdot k\cdot T}\right]-1\right\}-\frac{V_{\mathrm{out}\;}+R_{s}\cdot I_{\mathrm{out}\;}}{R_{sh}},$$

Thus, the single-diode model is fully characterized by five parameters: $\left(I_{ph},I_{o},a,{\;R}_{s},R_{sh}\right)$.

#### 5.1.2. Double Diode Model (DDM)

**Figure 12:**
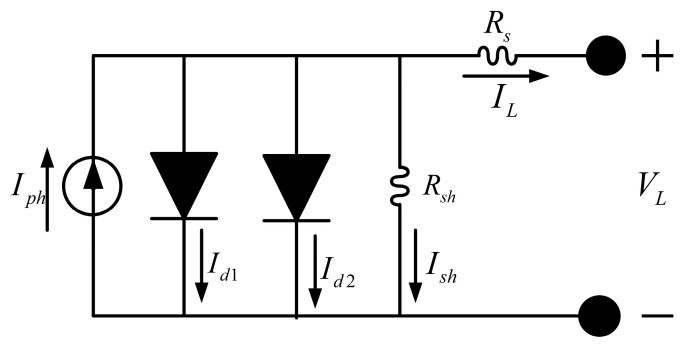
Equivalent circuit of the double diode model.

Compared with the single-diode model (SDM), the double-diode model (DDM) offers higher accuracy. This improvement arises from its explicit consideration of recombination losses in the depletion region. Accordingly, the DDM extends the equivalent circuit of the SDM by incorporating a second diode. The equivalent circuit of the double-diode model is illustrated in Figure 12 [10].

The output current of the double-diode model is expressed as:

(29) $$I_{out}=I_{ph}-I_{d1}-I_{d2}-I_{sh},$$

Combining the expressions for the diode currents and the shunt resistance, the $\left(I_{out}-V_{out}\right)$ characteristic equation of the double-diode model can be written as:

(30) $$I_{out}=I_{ph}-I_{o1}\cdot \left\{\exp\left[\frac{q\cdot \left(V_{\mathrm{out}\;}+R_{s}\cdot I_{\mathrm{out}\;}\right)}{a_{1}\cdot k\cdot T}\right]-1\right\}-I_{o2}\cdot \left\{\exp\left[\frac{q\cdot \left(V_{\mathrm{out}\;}+R_{s}\cdot I_{\mathrm{out}\;}\right)}{a_{2}\cdot k\cdot T}\right]-1\right\}\;-\frac{V_{\mathrm{out}\;}+R_{s}\cdot I_{\mathrm{out}\;}}{R_{sh}},$$

where $I_{d1}$ and $I_{d2}$ are the currents through diodes $D_{1}$ and $D_{2}$ respectively; $I_{o1}$ and $I_{o2}$ represent the saturation current and diffusion current; $a_{1}$ and $a_{2}$ are the ideality factor and diffusion factor.

Consequently, the double-diode model requires the estimation of seven unknown parameters: $\left(I_{ph},\;I_{o1},I_{o2},a_{1},a_{2},R_{s},R_{sh}\right)$.

### 5.2. Problem Formulation

The estimation of unknown parameters can be formulated as an optimization problem with an objective function g that quantifies the discrepancy between measured data and model-simulated results. The optimization goal is to minimize this discrepancy within a defined search space, thereby obtaining the optimal model parameters. Common error functions in such problems are expressed as follows [53,54,55].

(1) Objective function for the single-diode model (SDM):

(31) $$\left\{\begin{array}{c} g\left(V_{\mathrm{out}\;},I_{\mathrm{out}\;},y\right)=N_{p}\cdot I_{ph}-N_{p}\cdot I_{o}\cdot \left\{exp\left[\frac{q\cdot \left(\frac{V_{\mathrm{out}\;}}{N_{s}}+R_{s}\cdot \frac{I_{\mathrm{out}\;}}{N_{p}}\right)}{a\cdot k\cdot T}\right]-1\right\}-\\ N_{p}\cdot \frac{V_{\mathrm{out}\;}/N_{s}+R_{s}\cdot I_{\mathrm{out}\;}/N_{p}}{R_{\mathrm{sh}\;}}-I_{\mathrm{out}\;}\\ y=(I_{ph},I_{o},a,R_{s},R_{\mathrm{sh}\;}) \end{array}\right.,$$

(2) Objective function for the double-diode model (DDM):

(32) gVout ,Iout ,y=Np·Iph−Np⋅Io1⋅expq·Vout Ns+Rs·Iout Npa1·k·T−1−Np⋅Io2⋅expq·Vout Ns+Rs·Iout Npa2·k·T−1−Np·Vout Ns+Rs·Iout NpRsh −Iout y=(Iph,Io1,Io2,a1,a2,Rs,Rsh),

For a single cell, set $N_{s}=1$ and $N_{p}=1$.

Finally, the overall deviation between the experimental I−V curve and the model prediction is evaluated using the root mean square error (RMSE):

(33) $$RMSE(y)=\sqrt{\frac{\sum_{n=1}^{N}{\left(V_{\mathrm{out}\;,n},I_{\mathrm{out}\;,n},y\right)}^{2}}{N}},$$

where N is the total number of measured data points $\left(V_{\mathrm{out}\;,n},I_{\mathrm{out}\;,n}\right)$.

### 5.3. Experimental Results and Analysis

This section presents the experimental results to validate the effectiveness of the proposed MECOA. MECOA is primarily applied to estimate the unknown parameters in two photovoltaic models: the single-diode model (SDM) and the double-diode model (DDM). These models serve as the core interfaces for converting solar energy into electrical energy. When modeling such devices, meteorological data (irradiance and temperature) are used as input variables, while output variables can include voltage, current, and electrical power [3,4].

The experimental data were obtained from a Photowatt-PWP 201 photovoltaic module, composed of 36 series-connected polycrystalline silicon cells. Under conditions of 33 °C temperature and 1000 W/m^2^ irradiance, 26 sets of current–voltage (I–V) measurements were collected. Experiments were conducted to identify SDM and DDM parameters for the RTC France photovoltaic cells, and the results were compared with other high-performance optimization algorithms [13,56].

The competitor algorithms selected in this study (HHO, LSHADE, GRO, GWO, WOA, PSO, HSO, DBO, and the original COA) are highly representative of mainstream metaheuristic methods widely adopted in PV model parameter estimation research. These algorithms cover diverse categories of biomimetic and evolutionary optimization paradigms: GWO and WOA are classic swarm intelligence algorithms frequently used as baseline methods in PV parameter identification studies; PSO is a foundational particle swarm-based optimizer with extensive applications in renewable energy system optimization; LSHADE represents advanced differential evolution variants optimized for complex nonlinear problems, which is consistent with the nonlinearity of PV model parameter extraction; HHO, DBO, and GRO are recently proposed metaheuristics that have demonstrated competitiveness in engineering optimization tasks, including PV-related problems; HSO provides a contrast with metaphor-less optimization frameworks; and the original COA serves as a direct baseline to validate the effectiveness of MECOA’s improvements. By comparing MECOA with this diverse and representative set of algorithms, we can comprehensively verify its advantages in handling the strong nonlinearity, parameter coupling, and constraint characteristics of PV models—without requiring additional experiments on specialized PV-oriented algorithms, as the selected benchmarks already cover the core optimization capabilities needed for this task.

All algorithms were implemented in MATLAB 2024b and executed on a personal computer with a 2.5 GHz CPU, 16 GB RAM, and Windows 11. Each algorithm was independently run 30 times per problem, with a maximum of 500 iterations and a population size of 30. To highlight the differences between the proposed algorithm and other algorithms addressing similar problems, the Wilcoxon rank-sum test was employed to assess statistical significance. The ranges of unknown parameters for each model are summarized in Table 8 [56].

As described above, the root mean square error (RMSE) provides an intuitive measure of the discrepancy between measured data and simulated results. A smaller RMSE indicates that the simulated data closely match the measured data, which demonstrates the algorithm’s higher efficiency in identifying unknown parameters of the photovoltaic system. In other words, the diode model identified by this algorithm can more accurately characterize the actual behavior of solar cells and photovoltaic modules. Therefore, it is essential to minimize the error as much as possible.

Additionally, the absolute error (IAE) and relative error (RE) are employed to quantify the discrepancy at each measured voltage point. They are defined as follows [3,48]:

(34) $$IAE=\left|I_{measure}-I_{simulate}\right|,$$

(35) $$RE=\frac{I_{measure}-I_{simulate}}{I_{measure}},$$

#### 5.3.1. Results of SDM

Table 9 presents a systematic comparison of MECOA with eight other algorithms, including COA, GWO, PSO, and WOA, in the parameter identification of the single-diode model (SDM). The table not only shows the estimated values for five key parameters—photocurrent ($I_{ph}$), diode reverse saturation current ($I_{d}$), series resistance ($R_{s}$), among others—but also uses the root mean square error (RMSE) to intuitively illustrate differences in fitting accuracy. The results indicate that MECOA achieves the best fit with an RMSE of 9.8602 × 10^−4^, closely followed by DBO (9.9402 × 10^−4^) and LSHADE (9.8756 × 10^−4^). All other algorithms lag significantly: the original COA exhibits an RMSE of 2.0133 × 10^−2^, roughly 20 times that of MECOA, while HSO reaches 8.1496 × 10^−3^, more than eight times higher. Even comparatively better-performing algorithms such as GWO (1.1562 × 10^−3^) and PSO (1.0081 × 10^−3^) yield RMSE values higher than MECOA. These results strongly confirm that MECOA, through the synergy of elite-guided search, horizontal crossover, and precise elimination strategies, can accurately overcome the strong coupling and nonlinearity among SDM parameters, significantly reducing deviations between simulated and measured data and providing a higher-precision estimation approach for photovoltaic model parameter identification. The “/” symbol indicates a self-comparison (which is not meaningful in this context), and the “+” symbol denotes a statistically significant difference.

**Figure 13:**
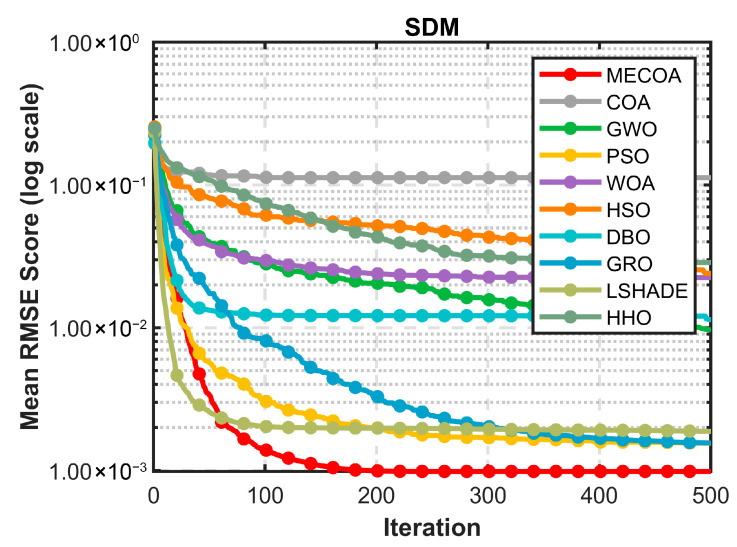
The convergence curve of MECOA and other algorithms on the SDM model.

Figure 13 further highlights MECOA’s advantage through convergence curves, reflecting its dynamic optimization process. Within the first 50 iterations, MECOA’s fitness rapidly drops to the order of 1.00 × 10^−3^, far surpassing COA, HSO, and other algorithms that remain above 1.00 × 10^−1^, demonstrating strong initial global exploration capability. During the mid-iterations (50–200), MECOA quickly enters a stable optimization phase, with the curve flattening. In the later iterations (200–500), the error remains consistently low without significant fluctuations. In contrast, comparative algorithms show slower convergence and higher variability: COA’s error remains above 1.00 × 10^−2^ even after 500 iterations, and WOA, HHO, and others exhibit noticeable fluctuations in the mid-iterations. This indicates that MECOA, leveraging the heavy-tail properties of Lévy flights for efficient global exploration, while reinforcing local exploitation via elite guidance, achieves both rapid convergence and stability, effectively avoiding premature convergence issues typical in traditional algorithms.

**Figure 14:**
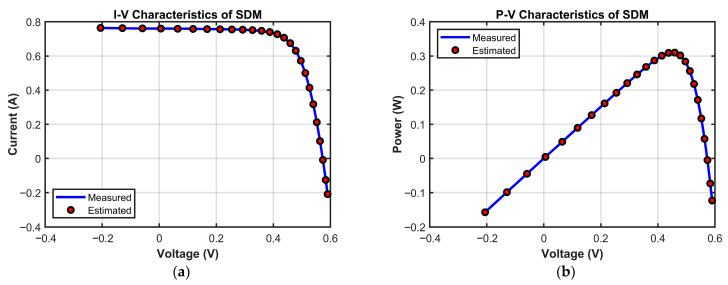
The I-V and P-V characteristics of the estimated SDM identified by MECOA. (**a**) I-V characteristic; (**b**) P-V characteristic.

Figure 14 validates MECOA’s reliability from the perspective of actual modeling by comparing I–V and P–V characteristic curves. In the I–V curve (Figure 14a), MECOA’s estimated curve almost completely coincides with the measured data points. The match is precise across all regions: the reverse current segment at low voltage, the stable current segment at medium voltage, and the rapidly decreasing current segment at high voltage, with no noticeable deviation. In the P–V curve (Figure 14b), MECOA accurately estimates both the power peak and the corresponding peak voltage, closely aligning with the measured results and capturing the core output characteristics of the photovoltaic module. This excellent fitting performance not only corroborates MECOA’s minimal RMSE in Table 9 but also demonstrates that the estimated SDM parameters faithfully represent the operational state of the photovoltaic module, providing a reliable model foundation for subsequent efficiency assessment and fault diagnosis in PV systems.

Table 10 further quantifies MECOA’s local fitting accuracy at each operating point of the SDM using the integral absolute error (IAE). The error distribution across 26 measured data points demonstrates its stability. For current IAE, most errors fall within the range of 1 × 10^−4^–1 × 10^−3^ A; for example, at voltage −0.2057 V (current 0.764 A), the IAE is only 8.77 × 10^−5^ A, and at 0.2924 V (current 0.754 A), the IAE is 3.36 × 10^−4^ A, indicating negligible deviation. Even in regions prone to fitting errors, such as high voltage (0.5833 V) or reverse voltage, the maximum IAE remains only 0.002507 A, well within a controllable range. For power IAE, the maximum error is 0.001463 W (0.5833 V) and the minimum is 1.8 × 10^−5^ W (−0.2057 V), with the total current IAE across all points summing to only 0.021527 A. These results show that MECOA not only achieves global optimal RMSE as in Table 9 but also maintains high stability in local voltage segments, with no significant deviations from measured data. This ensures robust fitting across different sections of the PV characteristic curves, providing reliable error control for the practical application of the SDM model.

#### 5.3.2. Results of DDM

Table 11 focuses on the parameter identification performance of the double-diode model (DDM), comparing MECOA with eight other algorithms, including COA, GWO, and PSO, across seven key parameters: photocurrent ($I_{ph}$), the two diode saturation currents ($I_{d1}/I_{d2}$), diode ideality factors ($n_{1}/n_{2}$), series resistance ($R_{s}$), and shunt resistance ($R_{sh}$). The table presents both the estimated values and the RMSE differences. The data indicate that MECOA achieves the best fit with an RMSE of 9.8249 × 10^−4^, closely matching DBO (9.9314 × 10^−4^), while all other algorithms exhibit significant gaps: the original COA has an RMSE of 6.0117 × 10^−3^, roughly six times that of MECOA, and HSO reaches 6.3207 × 10^−3^, more than six times higher. Even better-performing algorithms, such as PSO (1.0704 × 10^−3^) and GRO (1.1164 × 10^−3^), yield RMSE values higher than MECOA. These results confirm that, despite the increased parameter coupling and model complexity introduced by the additional diode in DDM, MECOA’s three improvement strategies remain highly effective. Specifically, elite-guided search provides multi-directional optimization targets, horizontal crossover promotes information sharing within the population, and precise elimination optimizes population quality, collectively enabling accurate estimation of all seven parameters and significantly reducing deviations between simulated and measured data. The “/” symbol indicates a self-comparison (which is not meaningful in this context), and the “+” symbol denotes a statistically significant difference.

**Figure 15:**
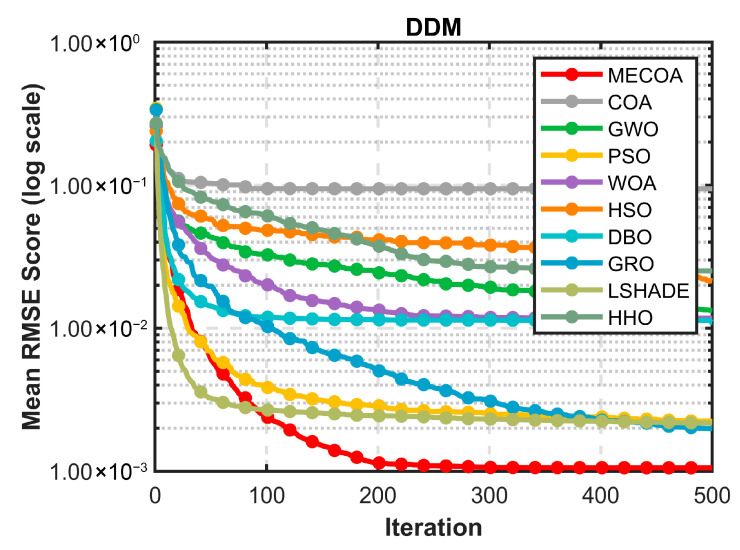
The convergence curve of MECOA and other algorithms on the DDM model.

Figure 15 further highlights MECOA’s advantage from the perspective of dynamic optimization. Within the first 50 iterations, MECOA’s fitness rapidly decreases to the order of 1.00 × 10^−3^, far surpassing COA, HSO, and other algorithms that remain above 1.00 × 10^−1^, demonstrating strong initial global exploration capability. During mid-iterations (50–200), MECOA quickly enters a stable optimization phase, with the curve flattening. In the later iterations (200–500), the error remains consistently low without noticeable fluctuation. In contrast, comparative algorithms exhibit slower convergence: COA’s error remains above 1.00 × 10^−2^ even after 500 iterations, and WOA, HHO, and others show significant mid-iteration oscillations. This indicates that MECOA, leveraging the heavy-tail properties of Lévy flights to overcome local optimum traps, while reinforcing local fine-tuning via elite guidance, achieves both rapid convergence and stability, effectively mitigating premature convergence issues in the highly complex DDM scenario.

**Figure 16:**
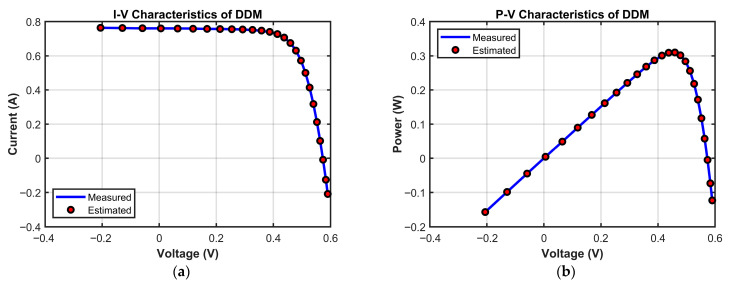
The I-V and P-V characteristics of the estimated DDM identified by MECOA. (**a**) I-V characteristic; (**b**) P-V characteristic.

Figure 16 validates MECOA’s modeling reliability from a practical application perspective through I–V and P–V characteristic curves. In the I–V curve (Figure 16a), MECOA’s estimated curve almost entirely coincides with the measured data points. Precise matching is achieved across all regions: the reverse current segment at low voltage, the stable current segment at medium voltage, and the rapidly decreasing current segment at high voltage, with no noticeable deviation. In the P–V curve (Figure 16b), MECOA accurately estimates both the power peak and the corresponding peak voltage, closely aligning with measured results and capturing the core output characteristics of the photovoltaic module. This excellent fitting performance not only corroborates MECOA’s minimal RMSE in Table 11 but also demonstrates that the estimated DDM parameters faithfully represent the operational state of the PV module. Notably, it accurately models depletion-region composite losses, a key feature captured by the DDM, providing a more practically relevant model foundation for subsequent efficiency assessment and fault diagnosis in PV systems.

Table 12 further quantifies MECOA’s local fitting accuracy at each operating point of the DDM using the integral absolute error (IAE). The error distribution across 26 measured data points demonstrates its stability. For current IAE, most errors fall within the range of 1 × 10^−5^–1 × 10^−3^ A; for example, at voltage −0.2057 V (current 0.764 A), the IAE is only 1.54 × 10^−5^ A, and at 0.0057 V (current 0.7605 A), the IAE is 3.26 × 10^−4^ A, indicating negligible deviation. Even in regions prone to fitting errors, such as high voltage (0.5833 V) or reverse voltage, the maximum IAE remains only 0.002507 A, within a controllable range. For power IAE, the maximum error is 0.001463 W (0.5833 V) and the minimum is 3.16 × 10^−6^ W (−0.2057 V), with the total current IAE across all points being extremely low. These results indicate that MECOA not only achieves global optimal RMSE as in Table 11 but also maintains high stability in local voltage segments, effectively addressing the fitting challenges introduced by increased parameters and model complexity in DDM, providing reliable error control for its engineering applications.

The improved parameter accuracy achieved by MECOA has significant engineering implications for PV systems: (1) In performance modeling, precise parameters enable more accurate prediction of I-V/P-V characteristics, supporting optimal system sizing and energy yield estimation; (2) In fault detection, reduced fitting error enhances the ability to distinguish between normal performance variations and abnormal conditions (e.g., module degradation, partial shading); (3) In control strategy optimization, accurate model parameters improve the efficiency of MPPT algorithms and grid integration control, especially under dynamic environmental conditions. These benefits directly contribute to the reliability and cost-effectiveness of PV energy utilization.

## 6. Conclusions

This study addresses the limitations of the conventional Coati Optimization Algorithm (COA) in global optimization and photovoltaic (PV) model parameter identification, such as insufficient global exploration, rapid loss of population diversity, and limited convergence accuracy. To overcome these issues, a Multi-Strategy Enhanced Coati Optimization Algorithm (MECOA) is proposed. By integrating three core improvement strategies, MECOA breaks through the bottlenecks of the original algorithm: the elite-guided search strategy constructs an elite pool from the top three individuals and combines Lévy flight to balance global exploration and local exploitation; the horizontal crossover strategy enhances collaborative search capability through individual information recombination; and the precision elimination mechanism removes 20% of low-fitness individuals per generation and generates new individuals around the optimal solution, improving overall population quality.

Experimental validation based on the CEC2017 (30, 50 and 100-dimensional) and CEC2022 (20-dimensional) test suites shows that MECOA maintains population diversity more effectively and dynamically balances early-stage exploration with late-stage exploitation. MECOA consistently outperforms other algorithms across both test suites: for example, the RMSE of F1 on CEC2017 is only one ten-thousandth of COA’s, and on CEC2022, MECOA achieves full superiority over five comparison algorithms. Wilcoxon and Friedman tests confirm that these advantages are statistically significant, with MECOA achieving the best average ranks (1.87, 2.07, 1.83 for CEC2017 and 1.58 for CEC2022).

In PV model parameter identification, MECOA also demonstrates excellent performance. For the single diode model (SDM), the RMSE of 9.8602 × 10^−4^ is near 1/500 of COA’s, and the IAE at each voltage point remains below 0.0016 A. For the double diode model (DDM), the RMSE is maintained below 9.8249 × 10^−4^, with a total IAE of only 0.021555 A, and the fitted curves align closely with measured data, providing reliable support for PV system simulation and optimization.

This study focuses on static parameter extraction for PV models under single irradiance and temperature conditions using standard datasets (Photowatt-PWP201 module and RTC France solar cell). Future work will address multi-condition parameter identification to adapt to dynamic environmental changes, investigate robustness to measurement noise, and explore integration with on-line maximum power point tracking (MPPT) and PV fault diagnostic systems. Additionally, extending MECOA to multi-objective optimization scenarios (e.g., balancing accuracy and computational efficiency) will further enhance its practical value in PV system design and operation.

## Figures and Tables

**Table 1:** Parameter settings of the comparison algorithms.

Algorithms	Parameter Name	Parameter Value	Reference
HHO	E0,E1	[−1, 1], [0, 2]	[37]
LSHADE	$p,r^{arc},r^{N^{init}},H$	0.11, 2.6, 18, 6	[38]
GRO	$r_{1},r_{2},r_{3},m$	[0, 1], [0, 1], [0, 1], [0, 1]	[39]
GWO	a	[0, 2]	[40]
WOA	r,l,A,b	[0, 1], [−1, 1], [0, 2], 1	[41]
PSO	$c_{1},c_{2},w$	1.5, 1.5, 0.8	[42]
HSO	α	3	[43]
DBO	$P_{percent}$	0.2	[44]
COA	I,r	$\left\{1,2\right\}$, [0, 1]	[26]
MECOA	$I,r,\delta ,{Elite}_{Size}$	$\left\{1,2\right\}$, [0, 1], [1, 0], 3	/

**Table 2:** Results of various algorithms tested on the CEC 2017 benchmark. (dim = 30).

ID	Metric	HHO	LSHADE	GRO	DBO	HSO	WOA	PSO	GWO	COA	MECOA
F1	mean	4.1107 × 10^8^	3.6721 × 10^5^	1.0753 × 10^8^	2.4805 × 10^8^	6.1181 × 10^3^	5.8582 × 10^9^	1.3247 × 10^9^	3.0686 × 10^9^	5.6810 × 10^10^	**3.1733 × 10^3^**
	std	3.1478 × 10^8^	5.3059 × 10^5^	5.6316 × 10^7^	1.7051 × 10^8^	6.0959 × 10^3^	1.7355 × 10^9^	8.1821 × 10^8^	1.6721 × 10^9^	8.7827 × 10^9^	**3.9694 × 10^3^**
F2	mean	1.5996 × 10^33^	1.2758 × 10^22^	1.8072 × 10^26^	3.0597 × 10^36^	3.0055 × 10^16^	5.1973 × 10^38^	3.1859 × 10^32^	7.0161 × 10^32^	2.9637 × 10^49^	**8.1099 × 10^15^**
	std	4.5164 × 10^33^	6.4351 × 10^22^	4.8210 × 10^26^	1.6441 × 10^37^	1.1000 × 10^17^	2.8455 × 10^39^	1.6787 × 10^33^	2.6766 × 10^33^	1.5159 × 10^50^	**4.2137 × 10^16^**
F3	mean	5.7432 × 10^4^	4.8043 × 10^4^	6.3291 × 10^4^	8.8863 × 10^4^	4.8713 × 10^4^	2.7515 × 10^5^	6.0481 × 10^4^	6.0888 × 10^4^	8.5224 × 10^4^	**4.4375 × 10^4^**
	std	7.0112 × 10^3^	4.4631 × 10^4^	1.2228 × 10^4^	1.2038 × 10^4^	1.4406 × 10^4^	7.6068 × 10^4^	1.7215 × 10^4^	1.1901 × 10^4^	**4.5502 × 10^3^**	7.6606 × 10^3^
F4	mean	7.0781 × 10^2^	5.1237 × 10^2^	5.6064 × 10^2^	6.6220 × 10^2^	7.0943 × 10^2^	1.2366 × 10^3^	6.2482 × 10^2^	6.4340 × 10^2^	1.5185 × 10^4^	**5.0212 × 10^2^**
	std	7.1959 × 10^1^	2.5528 × 10^1^	**2.5295 × 10^1^**	1.2569 × 10^2^	1.2090 × 10^2^	3.6325 × 10^2^	1.5635 × 10^2^	7.9575 × 10^1^	2.3636 × 10^3^	2.6193 × 10^1^
F5	mean	7.6289 × 10^2^	5.8202 × 10^2^	6.2409 × 10^2^	7.5349 × 10^2^	6.9545 × 10^2^	8.5705 × 10^2^	7.1783 × 10^2^	6.1740 × 10^2^	9.2241 × 10^2^	**5.8091 × 10^2^**
	std	3.7876 × 10^1^	**1.4737 × 10^1^**	1.7906 × 10^1^	4.3719 × 10^1^	3.0541 × 10^1^	4.6659 × 10^1^	2.5496 × 10^1^	3.3636 × 10^1^	3.1621 × 10^1^	2.9537 × 10^1^
F6	mean	6.6541 × 10^2^	6.0198 × 10^2^	6.0712 × 10^2^	6.4622 × 10^2^	6.5100 × 10^2^	6.8721 × 10^2^	6.2130 × 10^2^	6.1415 × 10^2^	6.8979 × 10^2^	**6.0150 × 10^2^**
	std	6.1745 × 10^0^	**1.5816 × 10^0^**	2.4867 × 10^0^	1.0706 × 10^1^	3.1641 × 10^0^	1.3323 × 10^1^	5.7538 × 10^0^	4.9727 × 10^0^	6.3279 × 10^0^	2.1166 × 10^0^
F7	mean	1.3274 × 10^3^	8.5957 × 10^2^	8.5449 × 10^2^	1.0492 × 10^3^	1.0777 × 10^3^	1.3225 × 10^3^	1.0022 × 10^3^	9.1280 × 10^2^	1.4279 × 10^3^	**8.2662 × 10^2^**
	std	5.4312 × 10^1^	3.4578 × 10^1^	**2.4253 × 10^1^**	9.4247 × 10^1^	1.0181 × 10^2^	8.7207 × 10^1^	2.4789 × 10^1^	5.5275 × 10^1^	4.3708 × 10^1^	5.0906 × 10^1^
F8	mean	9.8393 × 10^2^	8.7463 × 10^2^	9.0497 × 10^2^	1.0196 × 10^3^	1.0423 × 10^3^	1.0763 × 10^3^	1.0134 × 10^3^	9.0426 × 10^2^	1.1488 × 10^3^	**8.7379 × 10^2^**
	std	2.5492 × 10^1^	1.9124 × 10^1^	**1.5160 × 10^1^**	4.3889 × 10^1^	2.3301 × 10^1^	5.3430 × 10^1^	2.9905 × 10^1^	2.9971 × 10^1^	2.7291 × 10^1^	2.5496 × 10^1^
F9	mean	8.5035 × 10^3^	1.7322 × 10^3^	1.1860 × 10^3^	7.0417 × 10^3^	2.6027 × 10^3^	1.0028 × 10^4^	1.9539 × 10^3^	2.4580 × 10^3^	1.0977 × 10^4^	**1.0803 × 10^3^**
	std	1.2485 × 10^3^	4.6382 × 10^2^	**1.5450 × 10^2^**	1.9211 × 10^3^	8.5930 × 10^2^	2.6304 × 10^3^	9.4225 × 10^2^	1.0005 × 10^3^	1.5040 × 10^3^	2.7911 × 10^2^
F10	mean	6.2931 × 10^3^	4.6901 × 10^3^	5.7395 × 10^3^	6.4320 × 10^3^	**4.0828 × 10^3^**	7.4366 × 10^3^	7.3047 × 10^3^	4.9311 × 10^3^	8.8917 × 10^3^	7.1188 × 10^3^
	std	9.3116 × 10^2^	4.4642 × 10^2^	5.1852 × 10^2^	1.1906 × 10^3^	7.0217 × 10^2^	8.1064 × 10^2^	5.2445 × 10^2^	1.2447 × 10^3^	**3.6575 × 10^2^**	1.5274 × 10^3^
F11	mean	1.6280 × 10^3^	1.3580 × 10^3^	1.3250 × 10^3^	1.9608 × 10^3^	1.7492 × 10^3^	8.7987 × 10^3^	1.4762 × 10^3^	2.1669 × 10^3^	9.0969 × 10^3^	**1.1903 × 10^3^**
	std	1.6298 × 10^2^	4.5126 × 10^2^	5.5865 × 10^1^	8.8919 × 10^2^	1.5741 × 10^2^	2.6420 × 10^3^	6.3213 × 10^1^	8.4988 × 10^2^	2.3266 × 10^3^	**4.9143 × 10^1^**
F12	mean	9.8478 × 10^7^	**3.6417 × 10^5^**	3.6494 × 10^6^	5.9405 × 10^7^	5.0189 × 10^5^	4.8470 × 10^8^	7.4939 × 10^7^	1.2045 × 10^8^	1.4601 × 10^10^	1.1907 × 10^6^
	std	1.0465 × 10^8^	**2.9713 × 10^5^**	2.6326 × 10^6^	8.4824 × 10^7^	5.4005 × 10^5^	3.1206 × 10^8^	9.6553 × 10^7^	1.1781 × 10^8^	3.5273 × 10^9^	1.0900 × 10^6^
F13	mean	1.2179 × 10^6^	**1.7146 × 10^4^**	1.2029 × 10^5^	1.6934 × 10^7^	2.0771 × 10^4^	1.1126 × 10^7^	3.9640 × 10^7^	2.0814 × 10^7^	9.6377 × 10^9^	2.2917 × 10^4^
	std	1.2649 × 10^6^	1.5973 × 10^4^	1.8402 × 10^5^	3.0722 × 10^7^	**1.4337 × 10^4^**	8.7464 × 10^6^	1.9272 × 10^8^	4.6872 × 10^7^	4.6873 × 10^9^	2.3310 × 10^4^
F14	mean	1.3457 × 10^6^	**3.1133 × 10^3^**	3.6069 × 10^4^	3.2044 × 10^5^	2.1205 × 10^4^	2.3679 × 10^6^	1.3998 × 10^5^	8.8152 × 10^5^	6.4887 × 10^6^	7.1816 × 10^4^
	std	1.5358 × 10^6^	**8.0776 × 10^3^**	3.4594 × 10^4^	4.6497 × 10^5^	3.6500 × 10^4^	2.0160 × 10^6^	1.2514 × 10^5^	9.3720 × 10^5^	8.6577 × 10^6^	5.2037 × 10^4^
F15	mean	1.2239 × 10^5^	**3.5224 × 10^3^**	2.1646 × 10^4^	5.8622 × 10^4^	9.8225 × 10^3^	4.5198 × 10^6^	1.7119 × 10^5^	1.0203 × 10^6^	5.4757 × 10^8^	8.8567 × 10^3^
	std	7.2936 × 10^4^	5.2390 × 10^3^	1.6629 × 10^4^	5.9533 × 10^4^	**4.9667 × 10^3^**	7.3459 × 10^6^	1.1123 × 10^5^	1.7367 × 10^6^	3.0962 × 10^8^	8.9965 × 10^3^
F16	mean	3.6895 × 10^3^	2.6327 × 10^3^	**2.6062 × 10^3^**	3.3303 × 10^3^	2.9236 × 10^3^	4.3068 × 10^3^	3.1115 × 10^3^	2.7426 × 10^3^	6.1611 × 10^3^	2.6631 × 10^3^
	std	5.3181 × 10^2^	2.9160 × 10^2^	**1.8335 × 10^2^**	3.3558 × 10^2^	3.4835 × 10^2^	5.9082 × 10^2^	2.6156 × 10^2^	2.8738 × 10^2^	1.1732 × 10^3^	3.8557 × 10^2^
F17	mean	2.8578 × 10^3^	2.0643 × 10^3^	**1.9369 × 10^3^**	2.7256 × 10^3^	2.6632 × 10^3^	2.7775 × 10^3^	2.2630 × 10^3^	2.0469 × 10^3^	5.6839 × 10^3^	2.0522 × 10^3^
	std	3.3469 × 10^2^	1.7207 × 10^2^	**1.1749 × 10^2^**	3.0752 × 10^2^	2.9788 × 10^2^	3.5264 × 10^2^	2.3018 × 10^2^	1.6810 × 10^2^	3.6799 × 10^3^	1.4891 × 10^2^
F18	mean	3.7140 × 10^6^	**4.7849 × 10^4^**	6.8449 × 10^5^	3.7074 × 10^6^	1.6843 × 10^5^	1.4817 × 10^7^	1.7988 × 10^6^	1.6290 × 10^6^	6.4413 × 10^7^	9.4170 × 10^5^
	std	3.4751 × 10^6^	**3.6808 × 10^4^**	6.0971 × 10^5^	5.0565 × 10^6^	1.6412 × 10^5^	1.5112 × 10^7^	1.6360 × 10^6^	1.6282 × 10^6^	6.0122 × 10^7^	1.1129 × 10^6^
F19	mean	1.9945 × 10^6^	**4.3361 × 10^3^**	2.1302 × 10^4^	1.8693 × 10^6^	1.8342 × 10^4^	1.5633 × 10^7^	2.8549 × 10^6^	1.0543 × 10^7^	7.4352 × 10^8^	8.2078 × 10^3^
	std	1.1150 × 10^6^	9.8783 × 10^3^	5.4099 × 10^4^	3.2498 × 10^6^	4.2381 × 10^4^	1.6490 × 10^7^	1.3437 × 10^7^	3.2088 × 10^7^	3.9554 × 10^8^	**8.8601 × 10^3^**
F20	mean	2.8709 × 10^3^	2.4195 × 10^3^	**2.3463 × 10^3^**	2.7540 × 10^3^	2.5176 × 10^3^	2.8771 × 10^3^	2.5065 × 10^3^	2.4336 × 10^3^	3.0711 × 10^3^	2.3812 × 10^3^
	std	1.5088 × 10^2^	1.2340 × 10^2^	**8.8549 × 10^1^**	2.4627 × 10^2^	1.9814 × 10^2^	2.1093 × 10^2^	1.8572 × 10^2^	1.1878 × 10^2^	1.8456 × 10^2^	1.9558 × 10^2^
F21	mean	2.5767 × 10^3^	2.3743 × 10^3^	2.4107 × 10^3^	2.5310 × 10^3^	2.5665 × 10^3^	2.6327 × 10^3^	2.5109 × 10^3^	2.4066 × 10^3^	2.7608 × 10^3^	**2.3693 × 10^3^**
	std	4.6694 × 10^1^	**1.7178 × 10^1^**	1.9159 × 10^1^	6.4870 × 10^1^	1.8542 × 10^1^	6.6968 × 10^1^	2.7872 × 10^1^	2.4863 × 10^1^	4.4661 × 10^1^	2.5988 × 10^1^
F22	mean	7.0763 × 10^3^	3.6065 × 10^3^	2.3513 × 10^3^	4.6010 × 10^3^	4.9918 × 10^3^	8.0611 × 10^3^	6.3454 × 10^3^	5.0260 × 10^3^	9.7076 × 10^3^	**2.3013 × 10^3^**
	std	1.7546 × 10^3^	1.8614 × 10^3^	1.4691 × 10^1^	2.4308 × 10^3^	1.3508 × 10^3^	1.5788 × 10^3^	3.2120 × 10^3^	2.0110 × 10^3^	8.6244 × 10^2^	**2.2045 × 10^0^**
F23	mean	3.2605 × 10^3^	2.7347 × 10^3^	2.7566 × 10^3^	3.0130 × 10^3^	2.9075 × 10^3^	3.1371 × 10^3^	2.9434 × 10^3^	2.7733 × 10^3^	3.7039 × 10^3^	**2.7342 × 10^3^**
	std	1.7473 × 10^2^	2.1152 × 10^1^	2.2106 × 10^1^	8.6895 × 10^1^	**1.8873 × 10^1^**	1.0520 × 10^2^	7.9112 × 10^1^	4.6973 × 10^1^	1.7782 × 10^2^	2.5887 × 10^1^
F24	mean	3.4816 × 10^3^	2.9104 × 10^3^	2.9185 × 10^3^	3.1930 × 10^3^	3.0421 × 10^3^	3.2774 × 10^3^	3.1047 × 10^3^	2.9624 × 10^3^	3.7462 × 10^3^	**2.8996 × 10^3^**
	std	1.4435 × 10^2^	2.8065 × 10^1^	1.8105 × 10^1^	8.5674 × 10^1^	**1.3871 × 10^1^**	1.0372 × 10^2^	6.5260 × 10^1^	6.3363 × 10^1^	1.5763 × 10^2^	3.5803 × 10^1^
F25	mean	3.0103 × 10^3^	2.8950 × 10^3^	2.9372 × 10^3^	3.0284 × 10^3^	3.1767 × 10^3^	3.2322 × 10^3^	2.9603 × 10^3^	3.0333 × 10^3^	5.1718 × 10^3^	**2.8946 × 10^3^**
	std	3.3228 × 10^1^	**1.1559 × 10^1^**	1.6611 × 10^1^	2.0940 × 10^2^	1.0891 × 10^2^	8.2588 × 10^1^	3.4337 × 10^1^	8.3133 × 10^1^	4.8777 × 10^2^	1.5208 × 10^1^
F26	mean	8.1758 × 10^3^	4.6024 × 10^3^	**4.2376 × 10^3^**	7.1389 × 10^3^	5.2199 × 10^3^	8.5751 × 10^3^	5.1071 × 10^3^	5.0694 × 10^3^	1.1853 × 10^4^	4.4716 × 10^3^
	std	6.4388 × 10^2^	5.5303 × 10^2^	6.0748 × 10^2^	7.4041 × 10^2^	**4.2030 × 10^2^**	1.0148 × 10^3^	1.0494 × 10^3^	4.5537 × 10^2^	8.8811 × 10^2^	5.8138 × 10^2^
F27	mean	3.6346 × 10^3^	3.2360 × 10^3^	3.2765 × 10^3^	3.3379 × 10^3^	3.3371 × 10^3^	3.4997 × 10^3^	3.2736 × 10^3^	3.2725 × 10^3^	4.5804 × 10^3^	**3.2328 × 10^3^**
	std	1.7477 × 10^2^	1.3427 × 10^1^	1.8409 × 10^1^	6.9835 × 10^1^	6.1672 × 10^1^	1.4416 × 10^2^	3.3530 × 10^1^	3.2320 × 10^1^	4.4454 × 10^2^	**1.1764 × 10^1^**
F28	mean	3.5307 × 10^3^	3.2606 × 10^3^	3.3044 × 10^3^	3.6554 × 10^3^	3.6565 × 10^3^	3.7937 × 10^3^	3.3754 × 10^3^	3.4921 × 10^3^	7.5300 × 10^3^	**3.2315 × 10^3^**
	std	8.6854 × 10^1^	3.6030 × 10^1^	2.2459 × 10^1^	7.3159 × 10^2^	1.8736 × 10^2^	2.4560 × 10^2^	7.0268 × 10^1^	1.2826 × 10^2^	7.0124 × 10^2^	**2.2304 × 10^1^**
F29	mean	5.0347 × 10^3^	**3.7407 × 10^3^**	3.7759 × 10^3^	4.4793 × 10^3^	4.4948 × 10^3^	5.4635 × 10^3^	4.1717 × 10^3^	3.9361 × 10^3^	9.2036 × 10^3^	3.8064 × 10^3^
	std	5.0884 × 10^2^	**1.4172 × 10^2^**	1.4230 × 10^2^	4.0144 × 10^2^	2.5717 × 10^2^	5.5975 × 10^2^	1.9013 × 10^2^	2.1531 × 10^2^	3.4490 × 10^3^	2.1361 × 10^2^
F30	mean	1.1691 × 10^7^	1.7023 × 10^4^	4.5057 × 10^5^	2.4176 × 10^6^	9.2302 × 10^4^	5.7094 × 10^7^	2.7594 × 10^6^	1.1271 × 10^7^	1.6934 × 10^9^	**1.0534 × 10^4^**
	std	1.3088 × 10^7^	7.2449 × 10^3^	4.3308 × 10^5^	4.8580 × 10^6^	1.4973 × 10^5^	4.5369 × 10^7^	1.7944 × 10^6^	1.2721 × 10^7^	1.1804 × 10^9^	**3.7981 × 10^3^**

**Table 3:** Results of various algorithms tested on the CEC 2017 benchmark. (dim = 50).

ID	Metric	HHO	LSHADE	GRO	DBO	HSO	WOA	PSO	GWO	COA	MECOA
F1	mean	5.2137 × 10^9^	2.7695 × 10^8^	3.5930 × 10^9^	8.9757 × 10^9^	1.0068 × 10^5^	2.0660 × 10^10^	7.7516 × 10^9^	9.7982 × 10^9^	1.1208 × 10^11^	**2.7669 × 10^4^**
	std	1.5003 × 10^9^	3.9925 × 10^8^	1.5202 × 10^9^	1.5012 × 10^10^	8.3188 × 10^4^	5.6817 × 10^9^	4.6202 × 10^9^	4.6267 × 10^9^	9.6488 × 10^9^	**1.7790 × 10^4^**
F2	mean	4.3132 × 10^65^	**1.0000 × 10^30^**	5.4116 × 10^53^	2.4859 × 10^67^	4.9430 × 10^47^	1.2559 × 10^77^	8.0987 × 10^54^	2.7349 × 10^62^	1.3161 × 10^83^	3.7071 × 10^39^
	std	1.4622 × 10^66^	**1.4314 × 10^14^**	2.0785 × 10^54^	1.3600 × 10^68^	2.0930 × 10^48^	4.6640 × 10^77^	3.8897 × 10^55^	1.4980 × 10^63^	4.1915 × 10^83^	1.8199 × 10^40^
F3	mean	1.7078 × 10^5^	1.7506 × 10^5^	1.6484 × 10^5^	2.6913 × 10^5^	**1.3648 × 10^5^**	2.6093 × 10^5^	1.8969 × 10^5^	1.6823 × 10^5^	1.9843 × 10^5^	1.8536 × 10^5^
	std	**1.8550 × 10^4^**	8.8643 × 10^4^	2.2778 × 10^4^	8.1583 × 10^4^	2.4171 × 10^4^	8.8211 × 10^4^	3.9654 × 10^4^	2.6071 × 10^4^	2.8870 × 10^4^	3.2360 × 10^4^
F4	mean	1.9888 × 10^3^	6.6469 × 10^2^	1.0098 × 10^3^	1.4629 × 10^3^	1.2863 × 10^3^	5.0318 × 10^3^	1.2268 × 10^3^	1.6721 × 10^3^	3.6483 × 10^4^	**5.9120 × 10^2^**
	std	4.5606 × 10^2^	6.1843 × 10^1^	1.1026 × 10^2^	1.2690 × 10^3^	2.1753 × 10^2^	1.4348 × 10^3^	5.7083 × 10^2^	6.6605 × 10^2^	6.9315 × 10^3^	**5.6339 × 10^1^**
F5	mean	9.3303 × 10^2^	7.2541 × 10^2^	8.0597 × 10^2^	1.0027 × 10^3^	9.2838 × 10^2^	1.1100 × 10^3^	9.7267 × 10^2^	7.6811 × 10^2^	1.2028 × 10^3^	**6.9446 × 10^2^**
	std	**2.0267 × 10^1^**	3.0875 × 10^1^	3.6893 × 10^1^	1.1241 × 10^2^	4.1957 × 10^1^	8.0249 × 10^1^	4.4943 × 10^1^	6.1258 × 10^1^	3.1830 × 10^1^	7.1812 × 10^1^
F6	mean	6.8195 × 10^2^	6.1235 × 10^2^	6.2084 × 10^2^	6.6567 × 10^2^	6.6729 × 10^2^	6.9895 × 10^2^	6.3895 × 10^2^	6.2555 × 10^2^	7.0123 × 10^2^	**6.0562 × 10^2^**
	std	3.9053 × 10^0^	5.6072 × 10^0^	**3.6217 × 10^0^**	1.1998 × 10^1^	3.9612 × 10^0^	1.0233 × 10^1^	1.0817 × 10^1^	6.0654 × 10^0^	4.6698 × 10^0^	5.8574 × 10^0^
F7	mean	1.9164 × 10^3^	1.2341 × 10^3^	1.1008 × 10^3^	1.4311 × 10^3^	1.6413 × 10^3^	1.8950 × 10^3^	1.3490 × 10^3^	1.1801 × 10^3^	2.0383 × 10^3^	**1.0801 × 10^3^**
	std	7.8876 × 10^1^	1.0093 × 10^2^	**3.9732 × 10^1^**	1.4170 × 10^2^	2.3089 × 10^2^	9.6685 × 10^1^	4.6078 × 10^1^	1.1114 × 10^2^	7.2422 × 10^1^	2.1445 × 10^2^
F8	mean	1.2221 × 10^3^	1.0364 × 10^3^	1.0905 × 10^3^	1.2907 × 10^3^	1.2771 × 10^3^	1.4048 × 10^3^	1.2588 × 10^3^	1.0727 × 10^3^	1.4986 × 10^3^	**1.0314 × 10^3^**
	std	3.1994 × 10^1^	3.9365 × 10^1^	2.7816 × 10^1^	1.1376 × 10^2^	4.1288 × 10^1^	1.0383 × 10^2^	3.8484 × 10^1^	4.5336 × 10^1^	**2.3463 × 10^1^**	7.6378 × 10^1^
F9	mean	3.1401 × 10^4^	6.8519 × 10^3^	5.0537 × 10^3^	2.7782 × 10^4^	7.9430 × 10^3^	3.9481 × 10^4^	1.0073 × 10^4^	1.1896 × 10^4^	3.8158 × 10^4^	**1.9810 × 10^3^**
	std	2.6585 × 10^3^	2.1216 × 10^3^	1.5785 × 10^3^	7.1389 × 10^3^	1.9584 × 10^3^	1.0466 × 10^4^	5.5805 × 10^3^	4.5395 × 10^3^	2.6625 × 10^3^	**1.1159 × 10^3^**
F10	mean	1.0285 × 10^4^	8.6921 × 10^3^	1.0920 × 10^4^	1.1398 × 10^4^	**8.2248 × 10^3^**	1.3315 × 10^4^	1.3075 × 10^4^	8.4839 × 10^3^	1.5359 × 10^4^	1.2942 × 10^4^
	std	8.4606 × 10^2^	**4.5260 × 10^2^**	6.9223 × 10^2^	2.1790 × 10^3^	1.0465 × 10^3^	8.4120 × 10^2^	9.0843 × 10^2^	1.9989 × 10^3^	4.5876 × 10^2^	2.2758 × 10^3^
F11	mean	3.0738 × 10^3^	2.2073 × 10^3^	3.3910 × 10^3^	4.6514 × 10^3^	2.5835 × 10^3^	8.7510 × 10^3^	2.6476 × 10^3^	8.1711 × 10^3^	2.6956 × 10^4^	**1.4335 × 10^3^**
	std	9.1130 × 10^2^	2.2823 × 10^3^	9.4345 × 10^2^	1.9803 × 10^3^	3.3100 × 10^2^	2.7011 × 10^3^	4.0402 × 10^2^	3.3043 × 10^3^	1.8200 × 10^3^	**2.2445 × 10^2^**
F12	mean	9.4715 × 10^8^	1.4584 × 10^7^	1.3611 × 10^8^	7.8021 × 10^8^	6.7708 × 10^6^	4.1470 × 10^9^	2.8227 × 10^9^	2.1783 × 10^9^	8.6643 × 10^10^	**6.4281 × 10^6^**
	std	5.1821 × 10^8^	1.0570 × 10^7^	6.5411 × 10^7^	5.9931 × 10^8^	5.5294 × 10^6^	2.0643 × 10^9^	2.7947 × 10^9^	3.0386 × 10^9^	1.5839 × 10^10^	**4.4134 × 10^6^**
F13	mean	3.0242 × 10^7^	2.2900 × 10^4^	8.4599 × 10^5^	1.1186 × 10^8^	2.7526 × 10^4^	4.9172 × 10^8^	5.1265 × 10^8^	2.6768 × 10^8^	5.0561 × 10^10^	**7.7910 × 10^3^**
	std	2.9034 × 10^7^	1.2378 × 10^4^	1.1201 × 10^6^	1.7038 × 10^8^	1.7274 × 10^4^	3.1696 × 10^8^	8.8354 × 10^8^	2.4616 × 10^8^	1.5226 × 10^10^	**6.9980 × 10^3^**
F14	mean	6.0940 × 10^6^	**3.6508 × 10^4^**	3.7364 × 10^5^	2.9611 × 10^6^	7.6855 × 10^4^	6.7368 × 10^6^	9.4919 × 10^5^	1.8728 × 10^6^	8.7118 × 10^7^	6.2743 × 10^5^
	std	5.1373 × 10^6^	**4.0535 × 10^4^**	2.7082 × 10^5^	2.8786 × 10^6^	6.9697 × 10^4^	3.5199 × 10^6^	7.1701 × 10^5^	2.3118 × 10^6^	6.6283 × 10^7^	6.2620 × 10^5^
F15	mean	1.7014 × 10^6^	**8.1910 × 10^3^**	7.9025 × 10^4^	5.4275 × 10^7^	1.4101 × 10^4^	6.9414 × 10^7^	7.9300 × 10^6^	4.0723 × 10^7^	9.0674 × 10^9^	8.4934 × 10^3^
	std	1.9635 × 10^6^	**5.5806 × 10^3^**	1.2354 × 10^5^	1.5780 × 10^8^	5.6589 × 10^3^	1.1872 × 10^8^	6.0419 × 10^6^	7.9230 × 10^7^	3.4838 × 10^9^	5.7570 × 10^3^
F16	mean	4.9801 × 10^3^	3.5798 × 10^3^	3.4695 × 10^3^	4.8166 × 10^3^	3.6405 × 10^3^	6.5143 × 10^3^	4.4875 × 10^3^	**3.3981 × 10^3^**	1.0206 × 10^4^	3.5586 × 10^3^
	std	7.9662 × 10^2^	4.0721 × 10^2^	**3.5515 × 10^2^**	7.3828 × 10^2^	4.5550 × 10^2^	9.4506 × 10^2^	5.1954 × 10^2^	4.6782 × 10^2^	1.2064 × 10^3^	5.8769 × 10^2^
F17	mean	3.7664 × 10^3^	3.2546 × 10^3^	**2.9898 × 10^3^**	4.2541 × 10^3^	3.5523 × 10^3^	4.5371 × 10^3^	3.8485 × 10^3^	3.3370 × 10^3^	1.3011 × 10^4^	3.1295 × 10^3^
	std	3.7792 × 10^2^	**2.2740 × 10^2^**	2.3366 × 10^2^	4.9645 × 10^2^	2.6609 × 10^2^	5.1555 × 10^2^	3.7298 × 10^2^	5.7612 × 10^2^	8.9962 × 10^3^	4.6873 × 10^2^
F18	mean	1.1617 × 10^7^	**7.5696 × 10^5^**	4.1222 × 10^6^	1.4782 × 10^7^	1.1165 × 10^6^	6.5029 × 10^7^	7.9975 × 10^6^	1.0520 × 10^7^	1.7106 × 10^8^	3.4760 × 10^6^
	std	6.8261 × 10^6^	1.9250 × 10^6^	2.7732 × 10^6^	1.5731 × 10^7^	**1.0862 × 10^6^**	3.9097 × 10^7^	6.7805 × 10^6^	1.3932 × 10^7^	9.0701 × 10^7^	2.1292 × 10^6^
F19	mean	1.7317 × 10^6^	1.4982 × 10^4^	9.2581 × 10^4^	9.9115 × 10^6^	2.5985 × 10^4^	2.3461 × 10^7^	7.1907 × 10^6^	8.2127 × 10^6^	3.7551 × 10^9^	**1.3988 × 10^4^**
	std	1.1422 × 10^6^	**9.1014 × 10^3^**	2.1545 × 10^5^	1.0752 × 10^7^	2.1385 × 10^4^	2.8891 × 10^7^	5.3672 × 10^6^	1.2276 × 10^7^	1.7262 × 10^9^	1.2031 × 10^4^
F20	mean	3.5942 × 10^3^	3.4239 × 10^3^	**3.0063 × 10^3^**	3.6532 × 10^3^	3.0673 × 10^3^	3.9386 × 10^3^	3.6336 × 10^3^	3.2335 × 10^3^	4.2819 × 10^3^	3.3079 × 10^3^
	std	3.7340 × 10^2^	2.2836 × 10^2^	2.2848 × 10^2^	3.6243 × 10^2^	2.9160 × 10^2^	4.1794 × 10^2^	2.5685 × 10^2^	4.6445 × 10^2^	**1.8370 × 10^2^**	4.2664 × 10^2^
F21	mean	2.9464 × 10^3^	2.5170 × 10^3^	2.5715 × 10^3^	2.9119 × 10^3^	2.8687 × 10^3^	3.1022 × 10^3^	2.7787 × 10^3^	2.5804 × 10^3^	3.2759 × 10^3^	**2.4549 × 10^3^**
	std	8.7288 × 10^1^	4.0302 × 10^1^	3.5224 × 10^1^	7.4281 × 10^1^	**3.3916 × 10^1^**	1.2843 × 10^2^	4.3494 × 10^1^	8.2233 × 10^1^	9.7682 × 10^1^	7.0230 × 10^1^
F22	mean	1.2502 × 10^4^	1.0455 × 10^4^	**9.4722 × 10^3^**	1.1949 × 10^4^	9.8028 × 10^3^	1.4869 × 10^4^	1.3357 × 10^4^	1.0407 × 10^4^	1.6906 × 10^4^	1.1443 × 10^4^
	std	8.9190 × 10^2^	8.6257 × 10^2^	4.2045 × 10^3^	1.9901 × 10^3^	1.1162 × 10^3^	1.0439 × 10^3^	3.8655 × 10^3^	2.1141 × 10^3^	**5.4705 × 10^2^**	5.8185 × 10^3^
F23	mean	4.0450 × 10^3^	3.0010 × 10^3^	3.0366 × 10^3^	3.5214 × 10^3^	3.2832 × 10^3^	3.8144 × 10^3^	3.4247 × 10^3^	3.0619 × 10^3^	4.5956 × 10^3^	**2.9074 × 10^3^**
	std	2.5014 × 10^2^	5.4636 × 10^1^	3.1744 × 10^1^	1.3704 × 10^2^	**3.0180 × 10^1^**	1.8678 × 10^2^	1.3420 × 10^2^	9.3251 × 10^1^	1.7703 × 10^2^	5.9313 × 10^1^
F24	mean	4.3283 × 10^3^	3.1554 × 10^3^	3.2006 × 10^3^	3.7239 × 10^3^	3.3444 × 10^3^	3.9398 × 10^3^	3.5933 × 10^3^	3.2485 × 10^3^	4.8470 × 10^3^	**3.1116 × 10^3^**
	std	2.5913 × 10^2^	4.9311 × 10^1^	3.1883 × 10^1^	1.5509 × 10^2^	**1.7953 × 10^1^**	1.5273 × 10^2^	1.8624 × 10^2^	1.4830 × 10^2^	2.2433 × 10^2^	1.0775 × 10^2^
F25	mean	3.7779 × 10^3^	3.1591 × 10^3^	3.5461 × 10^3^	3.5848 × 10^3^	3.6828 × 10^3^	5.4046 × 10^3^	3.4402 × 10^3^	3.7204 × 10^3^	1.5812 × 10^4^	**3.1017 × 10^3^**
	std	1.8774 × 10^2^	5.2419 × 10^1^	1.3576 × 10^2^	1.1904 × 10^3^	2.3048 × 10^2^	7.3704 × 10^2^	3.1726 × 10^2^	2.8291 × 10^2^	1.3992 × 10^3^	**3.3099 × 10^1^**
F26	mean	1.2114 × 10^4^	6.6845 × 10^3^	6.7625 × 10^3^	1.1570 × 10^4^	8.2150 × 10^3^	1.4694 × 10^4^	7.3765 × 10^3^	7.2725 × 10^3^	1.7639 × 10^4^	**5.9019 × 10^3^**
	std	1.3101 × 10^3^	7.5030 × 10^2^	8.2691 × 10^2^	1.8257 × 10^3^	7.2507 × 10^2^	1.6923 × 10^3^	2.2415 × 10^3^	6.6936 × 10^2^	**6.4504 × 10^2^**	8.5417 × 10^2^
F27	mean	5.0551 × 10^3^	3.5206 × 10^3^	3.7872 × 10^3^	4.1305 × 10^3^	3.7101 × 10^3^	4.9531 × 10^3^	3.6234 × 10^3^	3.6636 × 10^3^	7.0554 × 10^3^	**3.4593 × 10^3^**
	std	5.2887 × 10^2^	1.0735 × 10^2^	9.6665 × 10^1^	2.6467 × 10^2^	1.4073 × 10^2^	7.2703 × 10^2^	1.6235 × 10^2^	9.0939 × 10^1^	8.0479 × 10^2^	**8.4314 × 10^1^**
F28	mean	4.9085 × 10^3^	3.5189 × 10^3^	4.0101 × 10^3^	6.3259 × 10^3^	5.2162 × 10^3^	6.2591 × 10^3^	4.1551 × 10^3^	4.6298 × 10^3^	1.3825 × 10^4^	**3.3766 × 10^3^**
	std	4.1988 × 10^2^	1.5575 × 10^2^	2.2614 × 10^2^	2.3474 × 10^3^	1.3029 × 10^3^	6.2880 × 10^2^	7.3827 × 10^2^	4.6716 × 10^2^	1.4379 × 10^3^	**3.9247 × 10^1^**
F29	mean	7.3728 × 10^3^	4.6400 × 10^3^	4.6102 × 10^3^	6.1678 × 10^3^	5.4541 × 10^3^	9.2553 × 10^3^	5.6736 × 10^3^	4.9276 × 10^3^	1.4366 × 10^5^	**4.4187 × 10^3^**
	std	7.4823 × 10^2^	**3.2113 × 10^2^**	3.2281 × 10^2^	1.0027 × 10^3^	4.2731 × 10^2^	1.8806 × 10^3^	4.7353 × 10^2^	4.0527 × 10^2^	1.1491 × 10^5^	3.9739 × 10^2^
F30	mean	1.4551 × 10^8^	3.2684 × 10^6^	2.4910 × 10^7^	4.7270 × 10^7^	7.0330 × 10^6^	3.5475 × 10^8^	1.0102 × 10^8^	1.5965 × 10^8^	8.5971 × 10^9^	**1.0628 × 10^6^**
	std	4.5237 × 10^7^	1.9997 × 10^6^	1.2072 × 10^7^	4.7052 × 10^7^	4.6356 × 10^6^	2.0793 × 10^8^	4.4565 × 10^7^	4.6354 × 10^7^	2.5061 × 10^9^	**2.5886 × 10^5^**

**Table 4:** Results of various algorithms tested on the CEC 2017 benchmark. (dim = 100).

ID	Metric	HHO	LSHADE	GRO	DBO	HSO	WOA	PSO	GWO	COA	MECOA
F1	mean	5.0850 × 10^10^	1.6435 × 10^10^	5.2779 × 10^10^	9.8544 × 10^10^	3.5068 × 10^9^	1.1043 × 10^11^	3.2798 × 10^10^	5.8304 × 10^10^	2.7212 × 10^11^	**2.1586 × 10^8^**
	std	8.3834 × 10^9^	5.1586 × 10^9^	9.4344 × 10^9^	7.3152 × 10^10^	3.0089 × 10^9^	1.3204 × 10^10^	7.9350 × 10^9^	9.2761 × 10^9^	1.1305 × 10^10^	**1.7219 × 10^8^**
F2	mean	7.5868 × 10^152^	**1.0000 × 10^30^**	1.7586 × 10^133^	1.1859 × 10^153^	4.4402 × 10^199^	1.2524 × 10^177^	2.1655 × 10^137^	1.2343 × 10^142^	7.1299 × 10^179^	1.3295 × 10^115^
	std	**6.5535 × 10^4^**	1.4314 × 10^14^	9.2327 × 10^133^	**6.5535 × 10^4^**	**6.5535 × 10^4^**	**6.5535 × 10^4^**	1.1861 × 10^138^	6.7605 × 10^142^	**6.5535 × 10^4^**	7.1622 × 10^115^
F3	mean	3.9241 × 10^5^	4.1216 × 10^5^	4.4569 × 10^5^	6.8364 × 10^5^	3.5691 × 10^5^	9.5747 × 10^5^	6.0785 × 10^5^	5.3827 × 10^5^	3.5450 × 10^5^	**3.2751 × 10^5^**
	std	1.3400 × 10^5^	1.2945 × 10^5^	6.4595 × 10^4^	2.9336 × 10^5^	3.5848 × 10^4^	1.3350 × 10^5^	1.3339 × 10^5^	7.7076 × 10^4^	1.5784 × 10^4^	**1.0279 × 10^4^**
F4	mean	9.5936 × 10^3^	2.2773 × 10^3^	6.0054 × 10^3^	1.7031 × 10^4^	3.0228 × 10^3^	2.1781 × 10^4^	4.2501 × 10^3^	6.0432 × 10^3^	1.0593 × 10^5^	**1.0000 × 10^3^**
	std	1.9007 × 10^3^	5.6810 × 10^2^	1.2622 × 10^3^	1.5737 × 10^4^	7.6859 × 10^2^	4.6744 × 10^3^	1.7074 × 10^3^	1.6353 × 10^3^	1.4308 × 10^4^	**1.0910 × 10^2^**
F5	mean	1.6852 × 10^3^	1.3141 × 10^3^	1.4139 × 10^3^	1.7061 × 10^3^	1.7023 × 10^3^	1.9697 × 10^3^	1.6838 × 10^3^	1.2670 × 10^3^	2.1216 × 10^3^	**1.2014 × 10^3^**
	std	5.8267 × 10^1^	9.7818 × 10^1^	7.0318 × 10^1^	2.0979 × 10^2^	6.8363 × 10^1^	1.4146 × 10^2^	1.0963 × 10^2^	1.5839 × 10^2^	**4.7867 × 10^1^**	2.2003 × 10^2^
F6	mean	6.9102 × 10^2^	**6.3442 × 10^2^**	6.4859 × 10^2^	6.8224 × 10^2^	6.9190 × 10^2^	7.0926 × 10^2^	6.7049 × 10^2^	6.4511 × 10^2^	7.1182 × 10^2^	6.3515 × 10^2^
	std	3.8493 × 10^0^	8.8115 × 10^0^	4.7306 × 10^0^	1.3744 × 10^1^	4.8096 × 10^0^	1.0108 × 10^1^	1.2135 × 10^1^	4.6405 × 10^0^	**2.7306 × 10^0^**	1.5950 × 10^1^
F7	mean	3.8058 × 10^3^	2.8700 × 10^3^	2.2995 × 10^3^	2.9231 × 10^3^	4.8637 × 10^3^	3.8031 × 10^3^	2.4200 × 10^3^	2.1653 × 10^3^	4.0342 × 10^3^	**1.7935 × 10^3^**
	std	9.2442 × 10^1^	3.3361 × 10^2^	1.4627 × 10^2^	3.0800 × 10^2^	6.2268 × 10^2^	1.2695 × 10^2^	1.1043 × 10^2^	1.2776 × 10^2^	**7.6417 × 10^1^**	2.5404 × 10^2^
F8	mean	2.1301 × 10^3^	1.6467 × 10^3^	1.6879 × 10^3^	2.1358 × 10^3^	2.0328 × 10^3^	2.3892 × 10^3^	2.0349 × 10^3^	1.5546 × 10^3^	2.6098 × 10^3^	**1.4382 × 10^3^**
	std	5.3542 × 10^1^	8.4025 × 10^1^	4.9459 × 10^1^	2.4099 × 10^2^	5.6925 × 10^1^	9.9540 × 10^1^	9.9295 × 10^1^	6.8111 × 10^1^	**4.4347 × 10^1^**	2.2488 × 10^2^
F9	mean	6.7254 × 10^4^	3.3841 × 10^4^	**3.0647 × 10^4^**	7.5889 × 10^4^	6.3396 × 10^4^	8.2395 × 10^4^	6.4347 × 10^4^	4.8549 × 10^4^	8.0031 × 10^4^	3.4714 × 10^4^
	std	4.8152 × 10^3^	6.9663 × 10^3^	6.6986 × 10^3^	8.7823 × 10^3^	1.4237 × 10^4^	1.7482 × 10^4^	1.5664 × 10^4^	1.0875 × 10^4^	**3.3729 × 10^3^**	1.6728 × 10^4^
F10	mean	2.4278 × 10^4^	2.1718 × 10^4^	2.6228 × 10^4^	2.9455 × 10^4^	2.3863 × 10^4^	2.9245 × 10^4^	2.9317 × 10^4^	2.0957 × 10^4^	3.2937 × 10^4^	**1.5984 × 10^4^**
	std	1.5748 × 10^3^	1.0770 × 10^3^	1.1263 × 10^3^	3.7516 × 10^3^	1.5601 × 10^3^	1.4456 × 10^3^	1.9591 × 10^3^	5.9230 × 10^3^	**6.8388 × 10^2^**	1.1235 × 10^3^
F11	mean	1.5197 × 10^5^	6.7825 × 10^4^	1.0781 × 10^5^	2.2728 × 10^5^	**5.5497 × 10^4^**	2.9342 × 10^5^	8.9120 × 10^4^	8.6754 × 10^4^	2.5362 × 10^5^	7.7407 × 10^4^
	std	3.4501 × 10^4^	4.7233 × 10^4^	1.5991 × 10^4^	6.8762 × 10^4^	**1.2860 × 10^4^**	1.0518 × 10^5^	2.9977 × 10^4^	1.7924 × 10^4^	5.3151 × 10^4^	2.1368 × 10^4^
F12	mean	1.1392 × 10^10^	6.5499 × 10^8^	5.0465 × 10^9^	8.2037 × 10^9^	1.5984 × 10^8^	3.0914 × 10^10^	1.1910 × 10^10^	1.1477 × 10^10^	2.0737 × 10^11^	**7.7277 × 10^7^**
	std	4.5191 × 10^9^	3.5281 × 10^8^	1.9784 × 10^9^	2.3030 × 10^9^	9.9900 × 10^7^	8.2367 × 10^9^	7.8319 × 10^9^	5.7806 × 10^9^	2.0105 × 10^10^	**2.7771 × 10^7^**
F13	mean	2.6950 × 10^8^	2.1303 × 10^5^	5.6758 × 10^7^	3.3783 × 10^8^	7.0344 × 10^4^	2.6985 × 10^9^	1.8565 × 10^9^	1.9527 × 10^9^	4.9729 × 10^10^	**8.2504 × 10^3^**
	std	1.7504 × 10^8^	2.0690 × 10^5^	2.7850 × 10^7^	1.8676 × 10^8^	2.7637 × 10^4^	1.1945 × 10^9^	2.0145 × 10^9^	1.7043 × 10^9^	5.2413 × 10^9^	**5.9638 × 10^3^**
F14	mean	1.0251 × 10^7^	1.5248 × 10^6^	6.4785 × 10^6^	1.7086 × 10^7^	**9.7551 × 10^5^**	1.9106 × 10^7^	1.1533 × 10^7^	9.0002 × 10^6^	1.1245 × 10^8^	2.4411 × 10^6^
	std	3.8292 × 10^6^	1.2058 × 10^6^	2.4473 × 10^6^	1.0985 × 10^7^	**4.9479 × 10^5^**	9.9207 × 10^6^	5.2883 × 10^6^	5.1168 × 10^6^	4.7743 × 10^7^	1.3030 × 10^6^
F15	mean	1.9120 × 10^7^	1.9242 × 10^4^	1.9285 × 10^6^	8.2431 × 10^7^	3.2171 × 10^4^	4.8168 × 10^8^	3.3454 × 10^8^	3.8948 × 10^8^	2.5908 × 10^10^	**4.6131 × 10^3^**
	std	2.5675 × 10^7^	8.1373 × 10^3^	1.5703 × 10^6^	1.3948 × 10^8^	1.3672 × 10^4^	1.7711 × 10^8^	4.3513 × 10^8^	5.0807 × 10^8^	4.9609 × 10^9^	**4.2903 × 10^3^**
F16	mean	1.0009 × 10^4^	7.4696 × 10^3^	7.7847 × 10^3^	9.4878 × 10^3^	7.3437 × 10^3^	1.7118 × 10^4^	9.9028 × 10^3^	**6.8399 × 10^3^**	2.5178 × 10^4^	7.6405 × 10^3^
	std	1.1714 × 10^3^	7.7777 × 10^2^	**5.8825 × 10^2^**	1.6834 × 10^3^	7.8964 × 10^2^	2.1157 × 10^3^	8.3863 × 10^2^	1.1068 × 10^3^	3.2079 × 10^3^	1.9559 × 10^3^
F17	mean	8.6345 × 10^3^	5.9419 × 10^3^	**5.3155 × 10^3^**	9.5544 × 10^3^	5.6776 × 10^3^	4.0400 × 10^4^	8.6387 × 10^3^	5.5572 × 10^3^	1.2528 × 10^7^	6.0978 × 10^3^
	std	1.7134 × 10^3^	6.8084 × 10^2^	**3.5960 × 10^2^**	1.3831 × 10^3^	5.0662 × 10^2^	9.6818 × 10^4^	1.1636 × 10^3^	5.8648 × 10^2^	1.1007 × 10^7^	1.0947 × 10^3^
F18	mean	8.8262 × 10^6^	**1.6907 × 10^6^**	9.7042 × 10^6^	2.8512 × 10^7^	1.9747 × 10^6^	2.1340 × 10^7^	1.6448 × 10^7^	9.4214 × 10^6^	2.7664 × 10^8^	4.2531 × 10^6^
	std	4.1571 × 10^6^	**9.2075 × 10^5^**	4.0797 × 10^6^	1.5911 × 10^7^	1.2130 × 10^6^	1.1216 × 10^7^	5.7230 × 10^6^	5.0643 × 10^6^	1.1413 × 10^8^	1.8128 × 10^6^
F19	mean	4.7876 × 10^7^	1.0794 × 10^5^	3.8311 × 10^6^	9.8608 × 10^7^	2.2061 × 10^4^	4.6681 × 10^8^	3.9595 × 10^8^	2.8434 × 10^8^	2.5921 × 10^10^	**5.7330 × 10^3^**
	std	2.6442 × 10^7^	2.2108 × 10^5^	3.0234 × 10^6^	1.0617 × 10^8^	2.7757 × 10^4^	3.3125 × 10^8^	2.2797 × 10^8^	2.6011 × 10^8^	4.6169 × 10^9^	**5.3334 × 10^3^**
F20	mean	6.1488 × 10^3^	6.3870 × 10^3^	5.5538 × 10^3^	6.8840 × 10^3^	**5.1464 × 10^3^**	7.3068 × 10^3^	6.9648 × 10^3^	5.9266 × 10^3^	8.0265 × 10^3^	6.0346 × 10^3^
	std	5.0949 × 10^2^	5.1708 × 10^2^	**3.2772 × 10^2^**	7.6237 × 10^2^	6.2550 × 10^2^	6.7620 × 10^2^	5.7093 × 10^2^	1.1543 × 10^3^	3.7455 × 10^2^	5.9908 × 10^2^
F21	mean	4.3740 × 10^3^	3.1534 × 10^3^	3.2081 × 10^3^	4.0195 × 10^3^	3.7161 × 10^3^	4.4563 × 10^3^	3.7606 × 10^3^	3.0936 × 10^3^	5.0783 × 10^3^	**2.8916 × 10^3^**
	std	1.9695 × 10^2^	1.4327 × 10^2^	5.9296 × 10^1^	1.6355 × 10^2^	**5.3238 × 10^1^**	2.0372 × 10^2^	1.3810 × 10^2^	1.4037 × 10^2^	2.2658 × 10^2^	1.3913 × 10^2^
F22	mean	2.7461 × 10^4^	2.4349 × 10^4^	2.8362 × 10^4^	2.8673 × 10^4^	2.5556 × 10^4^	3.2070 × 10^4^	3.2653 × 10^4^	**2.3191 × 10^4^**	3.5177 × 10^4^	2.8804 × 10^4^
	std	1.6569 × 10^3^	1.0176 × 10^3^	3.0779 × 10^3^	4.5582 × 10^3^	1.6409 × 10^3^	1.6899 × 10^3^	1.5858 × 10^3^	5.6784 × 10^3^	**7.9538 × 10^2^**	7.2798 × 10^3^
F23	mean	5.7779 × 10^3^	3.6422 × 10^3^	3.8781 × 10^3^	4.7517 × 10^3^	4.0017 × 10^3^	5.3556 × 10^3^	4.9548 × 10^3^	3.7084 × 10^3^	6.7788 × 10^3^	**3.2544 × 10^3^**
	std	4.1293 × 10^2^	1.0523 × 10^2^	6.9131 × 10^1^	2.9642 × 10^2^	**5.9166 × 10^1^**	3.2347 × 10^2^	2.5340 × 10^2^	8.9972 × 10^1^	3.2194 × 10^2^	7.6494 × 10^1^
F24	mean	8.2297 × 10^3^	4.3505 × 10^3^	4.6047 × 10^3^	5.9477 × 10^3^	4.6149 × 10^3^	6.7944 × 10^3^	5.8639 × 10^3^	4.4785 × 10^3^	1.0369 × 10^4^	**3.8012 × 10^3^**
	std	6.8145 × 10^2^	1.5558 × 10^2^	1.1001 × 10^2^	4.3238 × 10^2^	**8.8937 × 10^1^**	3.5570 × 10^2^	3.8534 × 10^2^	1.9610 × 10^2^	7.9820 × 10^2^	1.1188 × 10^2^
F25	mean	6.7515 × 10^3^	5.0019 × 10^3^	6.9760 × 10^3^	1.1317 × 10^4^	6.5505 × 10^3^	1.0791 × 10^4^	5.8914 × 10^3^	7.1036 × 10^3^	3.0020 × 10^4^	**3.6694 × 10^3^**
	std	5.6997 × 10^2^	5.1719 × 10^2^	7.7302 × 10^2^	6.6673 × 10^3^	8.3952 × 10^2^	1.0933 × 10^3^	1.0490 × 10^3^	8.3000 × 10^2^	1.7025 × 10^3^	**7.0659 × 10^1^**
F26	mean	3.1234 × 10^4^	1.6951 × 10^4^	1.9962 × 10^4^	2.5740 × 10^4^	1.9476 × 10^4^	3.8184 × 10^4^	2.0254 × 10^4^	1.7589 × 10^4^	5.3547 × 10^4^	**1.2418 × 10^4^**
	std	2.2384 × 10^3^	1.5965 × 10^3^	1.8900 × 10^3^	3.7105 × 10^3^	**1.2179 × 10^3^**	3.8575 × 10^3^	3.4570 × 10^3^	1.6380 × 10^3^	2.0847 × 10^3^	2.3066 × 10^3^
F27	mean	7.3081 × 10^3^	3.9532 × 10^3^	4.5824 × 10^3^	4.8279 × 10^3^	4.2539 × 10^3^	6.2389 × 10^3^	3.9877 × 10^3^	4.3722 × 10^3^	1.5259 × 10^4^	**3.5992 × 10^3^**
	std	1.1185 × 10^3^	1.8808 × 10^2^	2.4352 × 10^2^	5.6574 × 10^2^	2.0874 × 10^2^	1.2036 × 10^3^	2.9870 × 10^2^	1.7565 × 10^2^	1.7319 × 10^3^	**8.8412 × 10^1^**
F28	mean	9.3433 × 10^3^	6.8646 × 10^3^	9.0259 × 10^3^	1.9828 × 10^4^	1.5706 × 10^4^	1.4888 × 10^4^	6.8571 × 10^3^	9.5755 × 10^3^	3.0889 × 10^4^	**3.7730 × 10^3^**
	std	1.0648 × 10^3^	1.2605 × 10^3^	9.8975 × 10^2^	6.7889 × 10^3^	5.2411 × 10^3^	1.2098 × 10^3^	1.9675 × 10^3^	1.3731 × 10^3^	1.0995 × 10^3^	**7.9945 × 10^1^**
F29	mean	1.2958 × 10^4^	8.4151 × 10^3^	8.7955 × 10^3^	1.1632 × 10^4^	9.3537 × 10^3^	2.1642 × 10^4^	1.0937 × 10^4^	9.3243 × 10^3^	7.1789 × 10^5^	**6.5334 × 10^3^**
	std	1.5056 × 10^3^	6.4508 × 10^2^	**5.4654 × 10^2^**	1.7337 × 10^3^	5.5826 × 10^2^	4.2213 × 10^3^	9.2909 × 10^2^	8.5982 × 10^2^	3.9245 × 10^5^	7.7130 × 10^2^
F30	mean	7.5026 × 10^8^	5.1994 × 10^6^	4.6741 × 10^7^	2.8336 × 10^8^	1.0240 × 10^6^	2.6442 × 10^9^	1.4417 × 10^9^	1.2662 × 10^9^	4.3489 × 10^10^	**6.6470 × 10^4^**
	std	3.7454 × 10^8^	8.0547 × 10^6^	2.2900 × 10^7^	2.0682 × 10^8^	7.7077 × 10^5^	8.3361 × 10^8^	1.3296 × 10^9^	1.0593 × 10^9^	5.7026 × 10^9^	**4.2205 × 10^4^**

**Table 5:** Results of various algorithms tested on the CEC 2022 benchmark. (dim = 20).

ID	Metric	HHO	LSHADE	GRO	DBO	HSO	WOA	PSO	GWO	COA	MECOA
F1	mean	2.5020 × 10^4^	2.0844 × 10^4^	1.1523 × 10^4^	3.4854 × 10^4^	6.9839 × 10^3^	3.5309 × 10^4^	**6.1707 × 10^3^**	1.4939 × 10^4^	4.8494 × 10^4^	6.3581 × 10^3^
	std	7.9622 × 10^3^	2.0796 × 10^4^	3.3754 × 10^3^	9.4736 × 10^3^	3.5306 × 10^3^	1.4132 × 10^4^	4.1031 × 10^3^	4.5072 × 10^3^	1.4396 × 10^4^	**2.6046 × 10^3^**
F2	mean	5.6473 × 10^2^	4.5225 × 10^2^	4.6239 × 10^2^	5.1391 × 10^2^	5.6096 × 10^2^	6.4079 × 10^2^	4.8639 × 10^2^	4.9492 × 10^2^	3.1412 × 10^3^	**4.5168 × 10^2^**
	std	6.2359 × 10^1^	1.6089 × 10^1^	**1.1233 × 10^1^**	5.4477 × 10^1^	6.8850 × 10^1^	8.1424 × 10^1^	3.8022 × 10^1^	4.2415 × 10^1^	8.0479 × 10^2^	2.0567 × 10^1^
F3	mean	6.6414 × 10^2^	6.0102 × 10^2^	6.0171 × 10^2^	6.3396 × 10^2^	6.3699 × 10^2^	6.6710 × 10^2^	6.1091 × 10^2^	6.0795 × 10^2^	6.8067 × 10^2^	**6.0019 × 10^2^**
	std	7.1620 × 10^0^	1.4216 × 10^0^	5.7498 × 10^−1^	9.8236 × 10^0^	5.5161 × 10^0^	1.5568 × 10^1^	4.3013 × 10^0^	4.3004 × 10^0^	9.1194 × 10^0^	**3.2379 × 10^−1^**
F4	mean	8.8456 × 10^2^	**8.4077 × 10^2^**	8.4551 × 10^2^	9.0809 × 10^2^	9.1655 × 10^2^	9.3691 × 10^2^	9.0685 × 10^2^	8.6097 × 10^2^	9.7816 × 10^2^	8.4865 × 10^2^
	std	1.3611 × 10^1^	1.2088 × 10^1^	**1.0662 × 10^1^**	2.9836 × 10^1^	1.1827 × 10^1^	4.2020 × 10^1^	2.1665 × 10^1^	2.8044 × 10^1^	1.7881 × 10^1^	3.0290 × 10^1^
F5	mean	3.0452 × 10^3^	1.2054 × 10^3^	**9.2297 × 10^2^**	2.2795 × 10^3^	1.1005 × 10^3^	4.6551 × 10^3^	9.9209 × 10^2^	1.2627 × 10^3^	3.5843 × 10^3^	1.1355 × 10^3^
	std	2.6241 × 10^2^	2.3868 × 10^2^	**1.1269 × 10^1^**	6.5896 × 10^2^	1.7129 × 10^2^	2.0886 × 10^3^	3.8085 × 10^1^	3.1269 × 10^2^	3.2798 × 10^2^	5.7056 × 10^2^
F6	mean	1.9306 × 10^5^	5.7172 × 10^3^	8.8106 × 10^4^	1.1521 × 10^6^	**4.5929 × 10^3^**	7.0905 × 10^6^	2.0186 × 10^6^	2.8656 × 10^6^	2.5864 × 10^9^	6.6726 × 10^3^
	std	1.4743 × 10^5^	4.9782 × 10^3^	1.4887 × 10^5^	2.0816 × 10^6^	**3.7156 × 10^3^**	1.1621 × 10^7^	1.3833 × 10^6^	7.2868 × 10^6^	8.8284 × 10^8^	5.6779 × 10^3^
F7	mean	2.2192 × 10^3^	**2.0541 × 10^3^**	2.0550 × 10^3^	2.1615 × 10^3^	2.1305 × 10^3^	2.2503 × 10^3^	2.1028 × 10^3^	2.1043 × 10^3^	2.2229 × 10^3^	2.0574 × 10^3^
	std	6.7863 × 10^1^	1.5759 × 10^1^	**9.2004 × 10^0^**	7.2788 × 10^1^	3.1905 × 10^1^	8.8439 × 10^1^	4.3621 × 10^1^	4.0593 × 10^1^	3.8353 × 10^1^	2.2978 × 10^1^
F8	mean	2.3253 × 10^3^	2.2400 × 10^3^	2.2300 × 10^3^	2.3217 × 10^3^	2.4858 × 10^3^	2.3009 × 10^3^	2.2830 × 10^3^	2.2578 × 10^3^	2.4597 × 10^3^	**2.2262 × 10^3^**
	std	1.1230 × 10^2^	3.7019 × 10^1^	**2.3831 × 10^0^**	7.0321 × 10^1^	1.2476 × 10^2^	6.4976 × 10^1^	7.3121 × 10^1^	4.8767 × 10^1^	1.3446 × 10^2^	5.2111 × 10^0^
F9	mean	2.5549 × 10^3^	2.4808 × 10^3^	2.4837 × 10^3^	2.5079 × 10^3^	2.7196 × 10^3^	2.6059 × 10^3^	2.5079 × 10^3^	2.5350 × 10^3^	3.4909 × 10^3^	**2.4808 × 10^3^**
	std	5.0915 × 10^1^	7.1710 × 10^−2^	1.7872 × 10^0^	2.7544 × 10^1^	1.0935 × 10^2^	6.1629 × 10^1^	3.6250 × 10^1^	2.9324 × 10^1^	3.1304 × 10^2^	**1.0706 × 10** **^−^** **^3^**
F10	mean	4.2017 × 10^3^	**2.5044 × 10^3^**	2.5756 × 10^3^	3.1158 × 10^3^	3.8643 × 10^3^	5.0736 × 10^3^	3.9381 × 10^3^	3.4296 × 10^3^	6.1242 × 10^3^	2.5096 × 10^3^
	std	7.5501 × 10^2^	7.3701 × 10^1^	1.7652 × 10^2^	1.0763 × 10^3^	5.5343 × 10^2^	1.3868 × 10^3^	1.0598 × 10^3^	6.5754 × 10^2^	1.5881 × 10^3^	**3.3854 × 10^1^**
F11	mean	3.5679 × 10^3^	2.9253 × 10^3^	3.0197 × 10^3^	3.1244 × 10^3^	3.4762 × 10^3^	4.0140 × 10^3^	3.5482 × 10^3^	3.6394 × 10^3^	8.5693 × 10^3^	**2.9200 × 10^3^**
	std	8.7589 × 10^2^	1.2019 × 10^2^	1.2078 × 10^2^	1.8337 × 10^2^	2.0040 × 10^2^	5.4091 × 10^2^	3.9256 × 10^2^	4.1996 × 10^2^	5.1443 × 10^2^	**4.0684 × 10^1^**
F12	mean	3.2846 × 10^3^	2.9665 × 10^3^	2.9666 × 10^3^	3.0417 × 10^3^	3.0027 × 10^3^	3.1615 × 10^3^	2.9858 × 10^3^	2.9784 × 10^3^	3.5887 × 10^3^	**2.9582 × 10^3^**
	std	1.8799 × 10^2^	1.8551 × 10^1^	**1.0933 × 10^1^**	7.5363 × 10^1^	2.9013 × 10^1^	1.4165 × 10^2^	3.4969 × 10^1^	2.6680 × 10^1^	2.7026 × 10^2^	1.2357 × 10^1^

**Table 6:** Results for various algorithms on the CEC 2017 and CEC2022.

Statistical Results	HHO	LSHADE	GRO	DBO	HSO	WOA	PSO	GWO	COA
CEC2017 d = 30 (+/=/−)	(30/0/0)	(16/0/14)	(25/0/5)	(29/0/1)	(27/0/3)	(29/0/1)	(29/0/1)	(27/0/3)	(30/0/0)
CEC2017 d = 50 (+/=/−)	(30/0/0)	(23/0/7)	(26/0/4)	(29/0/1)	(27/1/2)	(28/0/2)	(27/0/3)	(27/0/3)	(29/0/1)
CEC2017 d = 100 (+/=/−)	(29/0/1)	(26/0/4)	(28/0/2)	(29/0/1)	(28/0/2)	(29/0/1)	(28/0/2)	(27/0/3)	(30/0/0)
CEC2022 d = 20 (+/=/−)	(12/0/0)	(8/0/4)	(9/0/3)	(12/0/0)	(10/0/2)	(12/0/0)	(11/0/1)	(12/0/0)	(12/0/0)

**Table 7:** Friedman mean rank test result.

Suites	CEC2017						CEC2022	
Dimensions	30		50		100		20	
Algorithms	M.R	T.R	M.R	T.R	M.R	T.R	M.R	T.R
HHO	7.47	8	6.90	8	6.50	7	5.75	7
LSHADE	2.03	2	2.43	2	2.63	2	8.83	9
GRO	3.13	3	3.47	3	4.30	4	5.50	6
DBO	6.20	7	6.60	7	6.87	8	2.50	2
HSO	4.97	4	4.00	4	4.07	3	5.08	4
WOA	8.83	9	8.87	9	8.77	9	5.42	5
PSO	5.53	6	5.97	6	6.10	6	8.50	8
GWO	5.03	5	4.77	5	4.30	4	2.92	3
COA	9.93	10	9.93	10	9.63	10	8.92	10
MECOA	**1.87**	**1**	**2.07**	**1**	**1.83**	**1**	**1.58**	**1**

**Table 8:** The range of unknown parameters for different PV models.

Parameters	Single Diode PV Models		Double Diode Models	
	Lower Bound	Upper Bound	Lower Bound	Upper Bound
$I_{ph}\;(A)$	0	1	0	2
$I_{d}\;(\mu A)$	0	1	0	50
$R_{s}\;(\Omega )$	0	0.5	0	2
$R_{sh\;}(\Omega )$	0	100	0	2000
n	1	2	1	50
$I_{d1\;}(\mu A)$	0	1	0	50
$I_{d2}\;(\mu A)$	0	1	0	50
$n_{1}$	1	2	1	50
$n_{1}$	1	2	1	50

**Table 9:** Comparison among different algorithms on SDM.

Algorithm	$I_{ph}\;\left(A\right)$	$I_{d}\;(\mu A)$	$R_{s}\;(\Omega )$	$R_{sh}\;(\Omega )$	n	RSME	sig
MECOA	0.760776	3.23 × 10^−7^	0.036377	53.71853	1.481184	**9.8602 × 10^−4^**	/
COA	0.736804	6.45 × 10^−7^	0.026129	48.3867	1.560194	2.0133 × 10^−2^	+
GWO	0.761306	3.36 × 10^−7^	0.036002	47.50041	1.485585	1.1562 × 10^−3^	+
PSO	0.760938	2.92 × 10^−7^	0.0368	50.47772	1.471148	1.0081 × 10^−3^	+
WOA	0.760176	6.47 × 10^−7^	0.033886	75.6056	1.554826	2.0407 × 10^−3^	+
HSO	0.763224	0.000001	0.029551	51.0347	1.602817	8.1496 × 10^−3^	+
DBO	0.760811	3.03 × 10^−7^	0.036638	51.80582	1.4747	9.9402 × 10^−4^	+
GRO	0.761088	3.33 × 10^−7^	0.036353	56.44247	1.484082	1.0534 × 10^−3^	+
LSHADE	0.760763	3.32 × 10^−7^	0.036262	54.51104	1.484096	9.8756 × 10^−4^	+
HHO	0.760011	5.56 × 10^−7^	0.03354	88.23509	1.537796	1.9496 × 10^−3^	+

**Table 10:** IAE of MECOA on SDM.

Algorithm	V (V)	I (A)	$I_{sim}\;(A)$	${IAE}_{I}\;(A)$	$P_{sim}\;(W)$	${IAE}_{p}\;(A)$
1	−0.2057	0.764	0.764088	8.77 × 10^−5^	−0.15717	1.8 × 10^−5^
2	−0.1291	0.762	0.762663	0.000663	−0.09846	8.56 × 10^−5^
3	−0.0588	0.7605	0.761355	0.000855	−0.04477	5.03 × 10^−5^
4	0.0057	0.7605	0.760154	0.000346	0.004333	1.97 × 10^−6^
5	0.0646	0.76	0.759055	0.000945	0.049035	6.1 × 10^−5^
6	0.1185	0.759	0.758042	0.000958	0.089828	0.000113
7	0.1678	0.757	0.757092	9.17 × 10^−5^	0.12704	1.54 × 10^−5^
8	0.2132	0.757	0.756141	0.000859	0.161209	0.000183
9	0.2545	0.7555	0.755087	0.000413	0.19217	0.000105
10	0.2924	0.754	0.753664	0.000336	0.220371	9.83 × 10^−5^
11	0.3269	0.7505	0.751391	0.000891	0.24563	0.000291
12	0.3585	0.7465	0.747354	0.000854	0.267926	0.000306
13	0.3873	0.7385	0.740117	0.001617	0.286647	0.000626
14	0.4137	0.728	0.727382	0.000618	0.300918	0.000256
15	0.4373	0.7065	0.706973	0.000473	0.309159	0.000207
16	0.459	0.6755	0.67528	0.00022	0.309954	0.000101
17	0.4784	0.632	0.630758	0.001242	0.301755	0.000594
18	0.496	0.573	0.571928	0.001072	0.283676	0.000532
19	0.5119	0.499	0.499607	0.000607	0.255749	0.000311
20	0.5265	0.413	0.413649	0.000649	0.217786	0.000342
21	0.5398	0.3165	0.31751	0.00101	0.171392	0.000545
22	0.5521	0.212	0.212155	0.000155	0.117131	8.55 × 10^−5^
23	0.5633	0.1035	0.102251	0.001249	0.057598	0.000703
24	0.5736	−0.01	−0.00872	0.001282	−0.005	0.000736
25	0.5833	−0.123	−0.12551	0.002507	−0.07321	0.001463
26	0.59	−0.21	−0.20847	0.001528	−0.123	0.000901
Sum of IAE	N/A	N/A	N/A	0.021526866	N/A	0.008730779

**Table 11:** Comparison among different algorithms on DDM.

Algorithm	$I_{ph}\;(A)$	$R_{s}\;(\Omega )$	$R_{sh}\;(\Omega )$	$I_{d1}\;(\mu A)$	$n_{1}$	$I_{d2}\;(\mu A)$	$n_{2}$	RSME	sig
MECOA	0.760781	0.036737	55.46508	7.42 × 10^−7^	2	2.26768 × 10^−7^	1.451308	**9.8249 × 10^−4^**	/
COA	0.760854	0.034999	22.04328	2.57 × 10^−7^	1.46486	1.19441 × 10^−7^	1.746827	6.0117 × 10^−3^	+
GWO	0.758741	0.036344	83.81247	2.85 × 10^−7^	1.473204	2.29143 × 10^−7^	1.817755	1.4900 × 10^−3^	+
PSO	0.760893	0.035505	57.09248	0	1.978735	3.96844 × 10^−7^	1.502243	1.0704 × 10^−3^	+
WOA	0.760363	0.034279	86.84143	5.01 × 10^−7^	2	5.63352 × 10^−7^	1.543155	2.3850 × 10^−3^	+
HSO	0.763077	0.032916	58.03336	9.61 × 10^−7^	1.608205	9.13978 × 10^−7^	2	6.3207 × 10^−3^	+
DBO	0.760747	0.036135	55.52361	3.36 × 10^−7^	1.485573	4.70286 × 10^−8^	1.976181	9.9314 × 10^−4^	+
GRO	0.760182	0.036359	73.42288	1.73 × 10^−7^	1.436726	7.27111 × 10^−7^	1.809215	1.1164 × 10^−3^	+
LSHADE	0.760625	0.035299	64.71443	2.79 × 10^−7^	1.780844	3.28378 × 10^−7^	1.490441	1.1489 × 10^−3^	+
HHO	0.759459	0.038787	68.62729	1.59 × 10^−7^	1.424276	8.08597 × 10^−8^	1.562643	1.8914 × 10^−3^	+

**Table 12:** IAE of MECOA on DDM.

Algorithm	V (V)	I (A)	$I_{sim}\;(A)$	${IAE}_{I}\;(A)$	$P_{sim}\;(W)$	${IAE}_{p}\;(A)$
1	−0.2057	0.764	0.763984622	1.53782 × 10^−5^	−0.157151637	3.1633 × 10^−6^
2	−0.1291	0.762	0.7626048	0.0006048	−0.09845228	7.80796 × 10^−5^
3	−0.0588	0.7605	0.761337939	0.000837939	−0.044766671	4.92708 × 10^−5^
4	0.0057	0.7605	0.76017361	0.00032639	0.00433299	1.86042 × 10^−6^
5	0.0646	0.76	0.759107134	0.000892866	0.049038321	5.76791 × 10^−5^
6	0.1185	0.759	0.75812057	0.00087943	0.089837288	0.000104212
7	0.1678	0.757	0.757187549	0.000187549	0.127056071	3.14707 × 10^−5^
8	0.2132	0.757	0.756242458	0.000757542	0.161230892	0.000161508
9	0.2545	0.7555	0.755176245	0.000323755	0.192192354	8.23956 × 10^−5^
10	0.2924	0.754	0.753721605	0.000278395	0.220388197	8.14028 × 10^−5^
11	0.3269	0.7505	0.751398897	0.000898897	0.2456323	0.00029385
12	0.3585	0.7465	0.747301852	0.000801852	0.267907714	0.000287464
13	0.3873	0.7385	0.740011687	0.001511687	0.286606526	0.000585476
14	0.4137	0.728	0.727248374	0.000751626	0.300862652	0.000310948
15	0.4373	0.7065	0.706851707	0.000351707	0.309106251	0.000153801
16	0.459	0.6755	0.675211521	0.000288479	0.309922088	0.000132412
17	0.4784	0.632	0.630761056	0.001238944	0.301756089	0.000592711
18	0.496	0.573	0.571994359	0.001005641	0.283709202	0.000498798
19	0.5119	0.499	0.499705332	0.000705332	0.25579916	0.00036106
20	0.5265	0.413	0.41373286	0.00073286	0.217830351	0.000385851
21	0.5398	0.3165	0.317545722	0.001045722	0.171411181	0.000564481
22	0.5521	0.212	0.212123062	0.000123062	0.117113142	6.79424 × 10^−5^
23	0.5633	0.1035	0.10216384	0.00133616	0.057548891	0.000752659
24	0.5736	−0.01	−0.008791274	0.001208726	−0.005042674	0.000693326
25	0.5833	−0.123	−0.125543202	0.002543202	−0.07322935	0.00148345
26	0.59	−0.21	−0.208372508	0.001627492	−0.12293978	0.00096022
Sum of IAE	N/A	N/A	N/A	0.021275434	N/A	0.00877549

## Data Availability

Data are contained within the article.

## Appendix A

**Table A1:** Statistical *p*-values for 9 algorithms in the CEC2017 (D = 30) evaluation.

ID	HHO	LSHADE	GRO	DBO	HSO	WOA	PSO	GWO	COA
F1	3.0199 × 10^−11^	3.3384 × 10^−11^	3.0199 × 10^−11^	3.0199 × 10^−11^	5.3221 × 10^−3^	3.0199 × 10^−11^	3.0199 × 10^−11^	3.0199 × 10^−11^	3.0199 × 10^−11^
F2	3.0199 × 10^−11^	5.4617 × 10^−9^	3.0199 × 10^−11^	3.0199 × 10^−11^	1.6955 × 10^−2^	3.0199 × 10^−11^	3.0199 × 10^−11^	3.0199 × 10^−11^	3.0199 × 10^−11^
F3	1.3594 × 10^−7^	2.0523 × 10^−3^	5.5329 × 10^−8^	3.0199 × 10^−11^	4.9178 × 10^−1^	3.0199 × 10^−11^	8.8829 × 10^−6^	5.5999 × 10^−7^	3.0199 × 10^−11^
F4	3.0199 × 10^−11^	8.2357 × 10^−2^	7.3803 × 10^−10^	8.9934 × 10^−11^	4.5043 × 10^−11^	3.0199 × 10^−11^	2.2273 × 10^−9^	3.6897 × 10^−11^	3.0199 × 10^−11^
F5	3.0199 × 10^−11^	1.4945 × 10^−1^	4.6856 × 10^−8^	3.3384 × 10^−11^	1.3289 × 10^−10^	3.0199 × 10^−11^	3.3384 × 10^−11^	8.8829 × 10^−6^	3.0199 × 10^−11^
F6	3.0199 × 10^−11^	1.0315 × 10^−2^	8.1014 × 10^−10^	3.0199 × 10^−11^	3.0199 × 10^−11^	3.0199 × 10^−11^	3.0199 × 10^−11^	5.4941 × 10^−11^	3.0199 × 10^−11^
F7	3.0199 × 10^−11^	6.9125 × 10^−4^	2.3885 × 10^−4^	1.6132 × 10^−10^	3.0199 × 10^−11^	3.0199 × 10^−11^	3.6897 × 10^−11^	2.5721 × 10^−7^	3.0199 × 10^−11^
F8	4.9752 × 10^−11^	6.6273 × 10^−1^	5.1857 × 10^−7^	3.6897 × 10^−11^	3.0199 × 10^−11^	3.0199 × 10^−11^	3.0199 × 10^−11^	2.9590 × 10^−5^	3.0199 × 10^−11^
F9	3.0199 × 10^−11^	1.0105 × 10^−8^	8.1465 × 10^−5^	3.0199 × 10^−11^	3.1589 × 10^−10^	3.0199 × 10^−11^	3.1967 × 10^−9^	2.3715 × 10^−10^	3.0199 × 10^−11^
F10	1.8368 × 10^−2^	1.5581 × 10^−8^	1.1143 × 10^−3^	7.9782 × 10^−2^	2.4386 × 10^−9^	9.8231 × 10^−1^	3.4783 × 10^−1^	2.1540 × 10^−6^	2.0338 × 10^−9^
F11	3.0199 × 10^−11^	1.8608 × 10^−6^	8.1014 × 10^−10^	3.0199 × 10^−11^	3.0199 × 10^−11^	3.0199 × 10^−11^	3.0199 × 10^−11^	3.3384 × 10^−11^	3.0199 × 10^−11^
F12	3.0199 × 10^−11^	5.2650 × 10^−5^	2.0023 × 10^−6^	3.3384 × 10^−11^	5.2640 × 10^−4^	3.0199 × 10^−11^	3.0199 × 10^−11^	6.0658 × 10^−11^	3.0199 × 10^−11^
F13	3.0199 × 10^−11^	5.7929 × 10^−1^	4.1127 × 10^−7^	6.7220 × 10^−10^	4.1191 × 10^−1^	3.0199 × 10^−11^	3.0199 × 10^−11^	1.0937 × 10^−10^	3.0199 × 10^−11^
F14	8.1014 × 10^−10^	8.9934 × 10^−11^	5.5699 × 10^−3^	9.7917 × 10^−5^	1.3853 × 10^−6^	7.3891 × 10^−11^	1.9883 × 10^−2^	1.8682 × 10^−5^	6.6955 × 10^−11^
F15	4.0772 × 10^−11^	1.0315 × 10^−2^	1.7479 × 10^−5^	2.0283 × 10^−7^	2.9205 × 10^−2^	3.0199 × 10^−11^	3.0199 × 10^−11^	9.9186 × 10^−11^	3.0199 × 10^−11^
F16	3.4971 × 10^−9^	8.3026 × 10^−1^	5.7929 × 10^−1^	5.0922 × 10^−8^	1.3272 × 10^−2^	3.3384 × 10^−11^	2.5974 × 10^−5^	4.5530 × 10^−1^	3.0199 × 10^−11^
F17	9.9186 × 10^−11^	8.8830 × 10^−1^	4.2259 × 10^−3^	4.1997 × 10^−10^	2.6099 × 10^−10^	7.3891 × 10^−11^	3.5638 × 10^−4^	5.4933 × 10^−1^	3.0199 × 10^−11^
F18	8.8411 × 10^−7^	6.6955 × 10^−11^	5.2978 × 10^−1^	1.9112 × 10^−2^	5.5999 × 10^−7^	3.6459 × 10^−8^	1.0035 × 10^−3^	1.4412 × 10^−2^	1.7769 × 10^−10^
F19	3.0199 × 10^−11^	8.3146 × 10^−3^	6.0971 × 10^−3^	4.5726 × 10^−9^	7.4827 × 10^−2^	3.0199 × 10^−11^	3.6897 × 10^−11^	3.0199 × 10^−11^	3.0199 × 10^−11^
F20	3.1589 × 10^−10^	3.3285 × 10^−1^	5.4933 × 10^−1^	4.8011 × 10^−7^	9.0688 × 10^−3^	2.4386 × 10^−9^	2.5101 × 10^−2^	1.9579 × 10^−1^	6.0658 × 10^−11^
F21	3.0199 × 10^−11^	1.0869 × 10^−1^	3.5201 × 10^−7^	7.3891 × 10^−11^	3.0199 × 10^−11^	3.0199 × 10^−11^	3.0199 × 10^−11^	1.8608 × 10^−6^	3.0199 × 10^−11^
F22	3.0199 × 10^−11^	1.7769 × 10^−10^	3.0199 × 10^−11^	3.0199 × 10^−11^	3.0199 × 10^−11^	3.0199 × 10^−11^	3.0199 × 10^−11^	3.0199 × 10^−11^	3.0199 × 10^−11^
F23	3.0199 × 10^−11^	6.2040 × 10^−1^	3.0059 × 10^−4^	3.0199 × 10^−11^	3.0199 × 10^−11^	3.0199 × 10^−11^	3.0199 × 10^−11^	1.8682 × 10^−5^	3.0199 × 10^−11^
F24	3.0199 × 10^−11^	5.0120 × 10^−2^	6.9125 × 10^−4^	3.0199 × 10^−11^	3.3384 × 10^−11^	3.0199 × 10^−11^	3.0199 × 10^−11^	2.8790 × 10^−6^	3.0199 × 10^−11^
F25	3.0199 × 10^−11^	7.7272 × 10^−2^	2.2273 × 10^−9^	2.6099 × 10^−10^	3.0199 × 10^−11^	3.0199 × 10^−11^	3.4742 × 10^−10^	3.0199 × 10^−11^	3.0199 × 10^−11^
F26	3.0199 × 10^−11^	1.4128 × 10^−1^	4.8252 × 10^−1^	3.0199 × 10^−11^	3.2555 × 10^−7^	3.0199 × 10^−11^	1.0315 × 10^−2^	2.7726 × 10^−5^	3.0199 × 10^−11^
F27	3.0199 × 10^−11^	3.1119 × 10^−1^	2.6099 × 10^−10^	6.1210 × 10^−10^	5.4941 × 10^−11^	3.0199 × 10^−11^	2.6015 × 10^−8^	1.8731 × 10^−7^	3.0199 × 10^−11^
F28	3.0199 × 10^−11^	9.0307 × 10^−4^	9.9186 × 10^−11^	3.6897 × 10^−11^	3.0199 × 10^−11^	3.0199 × 10^−11^	3.0199 × 10^−11^	3.0199 × 10^−11^	3.0199 × 10^−11^
F29	3.0199 × 10^−11^	2.4581 × 10^−1^	5.5923 × 10^−1^	3.4971 × 10^−9^	1.2057 × 10^−10^	3.0199 × 10^−11^	9.8329 × 10^−8^	3.0317 × 10^−2^	3.0199 × 10^−11^
F30	3.0199 × 10^−11^	5.2650 × 10^−5^	3.0199 × 10^−11^	3.0199 × 10^−11^	3.4971 × 10^−9^	3.0199 × 10^−11^	3.0199 × 10^−11^	3.0199 × 10^−11^	3.0199 × 10^−11^

**Table A2:** Statistical *p*-values for 9 algorithms in the CEC2017 (D = 50) evaluation.

ID	HHO	LSHADE	GRO	DBO	HSO	WOA	PSO	GWO	COA
F1	3 × 10^−11^	3.0199 × 10^−11^	3.0199 × 10^−11^	3.01986 × 10^−11^	5.18568 × 10^−7^	3.01986 × 10^−11^	3.01986 × 10^−11^	3.0199 × 10^−11^	3.01986 × 10^−11^
F2	3 × 10^−11^	3.3593 × 10^−11^	3.0199 × 10^−11^	3.01986 × 10^−11^	4.1825 × 10^−9^	3.01986 × 10^−11^	3.01986 × 10^−11^	3.0199 × 10^−11^	3.01986 × 10^−11^
F3	0.04841	0.38709978	0.01031467	2.67842 × 10^−6^	1.8731 × 10^−7^	0.000104066	0.830255284	0.03916707	0.111986872
F4	3 × 10^−11^	3.8307 × 10^−5^	3.0199 × 10^−11^	3.01986 × 10^−11^	3.01986 × 10^−11^	3.01986 × 10^−11^	3.01986 × 10^−11^	3.0199 × 10^−11^	3.01986 × 10^−11^
F5	4.5 × 10^−11^	3.8307 × 10^−5^	8.352 × 10^−8^	1.77691 × 10^−10^	1.46431 × 10^−10^	3.01986 × 10^−11^	3.68973 × 10^−11^	1.7294 × 10^−7^	3.01986 × 10^−11^
F6	3 × 10^−11^	3.3242 × 10^−6^	8.891 × 10^−10^	3.01986 × 10^−11^	3.01986 × 10^−11^	3.01986 × 10^−11^	4.97517 × 10^−11^	2.8716 × 10^−10^	3.01986 × 10^−11^
F7	5 × 10^−11^	1.1077 × 10^−6^	0.00761706	1.84999 × 10^−8^	2.43863 × 10^−9^	7.38908 × 10^−11^	9.26029 × 10^−9^	9.2113 × 10^−5^	3.01986 × 10^−11^
F8	3.8 × 10^−10^	0.37903631	3.5923 × 10^−5^	2.87158 × 10^−10^	8.15274 × 10^−11^	3.01986 × 10^−11^	1.46431 × 10^−10^	0.00422592	3.01986 × 10^−11^
F9	3 × 10^−11^	1.0937 × 10^−10^	4.1825 × 10^−9^	3.01986 × 10^−11^	4.97517 × 10^−11^	3.01986 × 10^−11^	8.15274 × 10^−11^	4.0772 × 10^−11^	3.01986 × 10^−11^
F10	1 × 10^−5^	1.85 × 10^−8^	5.9706 × 10^−5^	0.012732115	9.26029 × 10^−9^	0.464272911	0.153667235	2.0283 × 10^−7^	2.22727 × 10^−9^
F11	5.5 × 10^−11^	6.5261 × 10^−7^	4.5043 × 10^−11^	4.97517 × 10^−11^	9.91863 × 10^−11^	3.01986 × 10^−11^	8.15274 × 10^−11^	3.0199 × 10^−11^	3.01986 × 10^−11^
F12	3 × 10^−11^	7.6588 × 10^−5^	3.0199 × 10^−11^	3.01986 × 10^−11^	1	3.01986 × 10^−11^	3.01986 × 10^−11^	3.0199 × 10^−11^	3.01986 × 10^−11^
F13	3 × 10^−11^	2.5721 × 10^−7^	3.3384 × 10^−11^	3.01986 × 10^−11^	1.10234 × 10^−8^	3.01986 × 10^−11^	3.01986 × 10^−11^	3.0199 × 10^−11^	3.01986 × 10^−11^
F14	8.1 × 10^−10^	1.4643 × 10^−10^	0.12967022	6.04595 × 10^−7^	5.46175 × 10^−9^	6.06576 × 10^−11^	0.00728836	0.00076973	3.01986 × 10^−11^
F15	3 × 10^−11^	0.92344213	3.9648 × 10^−8^	3.01986 × 10^−11^	0.000952074	3.01986 × 10^−11^	3.01986 × 10^−11^	3.0199 × 10^−11^	3.01986 × 10^−11^
F16	1.1 × 10^−8^	0.42038633	0.83025528	5.53286 × 10^−8^	0.347827783	3.01986 × 10^−11^	4.80107 × 10^−7^	0.45529691	3.01986 × 10^−11^
F17	5.1 × 10^−6^	0.05942792	0.17612755	2.0338 × 10^−9^	0.000283887	1.46431 × 10^−10^	4.80107 × 10^−7^	0.28377805	3.01986 × 10^−11^
F18	6 × 10^−7^	3.8249 × 10^−9^	0.52014461	9.79171 × 10^−5^	4.11271 × 10^−7^	3.01986 × 10^−11^	0.00022539	0.01563812	3.01986 × 10^−11^
F19	3 × 10^−11^	0.44641944	0.00055611	2.37147 × 10^−10^	0.010314672	3.01986 × 10^−11^	3.01986 × 10^−11^	3.0199 × 10^−11^	3.01986 × 10^−11^
F20	0.01221	0.36322231	0.00289133	0.002499392	0.016954881	4.11776 × 10^−6^	0.003033948	0.2580515	4.07716 × 10^−11^
F21	3 × 10^−11^	3.3242 × 10^−6^	1.1023 × 10^−8^	3.01986 × 10^−11^	3.01986 × 10^−11^	3.01986 × 10^−11^	3.33839 × 10^−11^	1.2023 × 10^−8^	3.01986 × 10^−11^
F22	0.15798	0.00556994	0.03643886	0.145319127	0.004032978	0.304176818	0.864993706	0.02068075	8.10136 × 10^−10^
F23	3 × 10^−11^	9.8329 × 10^−8^	8.1014 × 10^−10^	3.01986 × 10^−11^	3.01986 × 10^−11^	3.01986 × 10^−11^	3.01986 × 10^−11^	2.2273 × 10^−9^	3.01986 × 10^−11^
F24	3 × 10^−11^	0.01628481	0.00090307	3.01986 × 10^−11^	4.97517 × 10^−11^	3.01986 × 10^−11^	3.01986 × 10^−11^	0.00015846	3.01986 × 10^−11^
F25	3 × 10^−11^	2.7726 × 10^−5^	3.0199 × 10^−11^	3.01986 × 10^−11^	3.01986 × 10^−11^	3.01986 × 10^−11^	3.01986 × 10^−11^	3.0199 × 10^−11^	3.01986 × 10^−11^
F26	3.3 × 10^−11^	8.6634 × 10^−5^	5.8587 × 10^−6^	4.50432 × 10^−11^	3.82016 × 10^−10^	3.01986 × 10^−11^	0.025101283	8.4848 × 10^−9^	3.01986 × 10^−11^
F27	3 × 10^−11^	0.02708632	6.0658 × 10^−11^	3.01986 × 10^−11^	8.89099 × 10^−10^	3.01986 × 10^−11^	5.26501 × 10^−5^	2.0338 × 10^−9^	3.01986 × 10^−11^
F28	3 × 10^−11^	5.5329 × 10^−8^	3.0199 × 10^−11^	3.01986 × 10^−11^	3.01986 × 10^−11^	3.01986 × 10^−11^	3.33839 × 10^−11^	3.0199 × 10^−11^	3.01986 × 10^−11^
F29	3 × 10^−11^	0.01501413	0.03778202	5.07231 × 10^−10^	2.66947 × 10^−9^	3.01986 × 10^−11^	2.1544 × 10^−10^	2.4327 × 10^−5^	3.01986 × 10^−11^
F30	3 × 10^−11^	3.1589 × 10^−10^	3.0199 × 10^−11^	3.01986 × 10^−11^	4.19968 × 10^−10^	3.01986 × 10^−11^	3.01986 × 10^−11^	3.0199 × 10^−11^	3.01986 × 10^−11^

**Table A3:** Statistical *p*-values for 9 algorithms in the CEC2017 (D = 100) evaluation.

ID	HHO	LSHADE	GRO	DBO	HSO	WOA	PSO	GWO	COA
F1	3.0199 × 10^−11^	3.0199 × 10^−11^	3.0199 × 10^−11^	3.0199 × 10^−11^	3.3384 × 10^−11^	3.0199 × 10^−11^	3.0199 × 10^−11^	3.0199 × 10^−11^	3.0199 × 10^−11^
F2	3.0199 × 10^−11^	1.2118 × 10^−12^	3.0199 × 10^−11^	3.0199 × 10^−11^	3.0199 × 10^−11^	3.0199 × 10^−11^	3.3384 × 10^−11^	5.4941 × 10^−11^	3.0199 × 10^−11^
F3	2.7829 × 10^−7^	6.0971 × 10^−3^	4.5043 × 10^−11^	3.0199 × 10^−11^	1.4423 × 10^−3^	3.0199 × 10^−11^	3.0199 × 10^−11^	3.0199 × 10^−11^	8.4848 × 10^−9^
F4	3.0199 × 10^−11^	3.0199 × 10^−11^	3.0199 × 10^−11^	3.0199 × 10^−11^	3.0199 × 10^−11^	3.0199 × 10^−11^	3.0199 × 10^−11^	3.0199 × 10^−11^	3.0199 × 10^−11^
F5	4.9752 × 10^−11^	2.9205 × 10^−2^	3.5923 × 10^−5^	1.6947 × 10^−9^	6.0658 × 10^−11^	3.0199 × 10^−11^	1.6132 × 10^−10^	5.1060 × 10^−1^	3.0199 × 10^−11^
F6	3.0199 × 10^−11^	9.7052 × 10^−1^	2.4994 × 10^−3^	3.0199 × 10^−11^	3.0199 × 10^−11^	3.0199 × 10^−11^	3.8202 × 10^−10^	1.5638 × 10^−2^	3.0199 × 10^−11^
F7	3.0199 × 10^−11^	3.0199 × 10^−11^	2.6099 × 10^−10^	3.0199 × 10^−11^	3.0199 × 10^−11^	3.0199 × 10^−11^	3.3384 × 10^−11^	2.3897 × 10^−8^	3.0199 × 10^−11^
F8	3.0199 × 10^−11^	1.3250 × 10^−4^	3.5923 × 10^−5^	3.1589 × 10^−10^	3.0199 × 10^−11^	3.0199 × 10^−11^	4.0772 × 10^−11^	1.5969 × 10^−3^	3.0199 × 10^−11^
F9	1.4110 × 10^−9^	5.4933 × 10^−1^	7.5059 × 10^−1^	1.7769 × 10^−10^	2.3768 × 10^−7^	1.7769 × 10^−10^	1.4733 × 10^−7^	7.6973 × 10^−4^	3.6897 × 10^−11^
F10	3.0199 × 10^−11^	3.3384 × 10^−11^	3.0199 × 10^−11^	3.0199 × 10^−11^	3.0199 × 10^−11^	3.0199 × 10^−11^	3.0199 × 10^−11^	3.5201 × 10^−7^	3.0199 × 10^−11^
F11	8.9934 × 10^−11^	4.2067 × 10^−2^	4.4440 × 10^−7^	3.0199 × 10^−11^	1.5292 × 10^−5^	3.0199 × 10^−11^	2.9047 × 10^−1^	1.1882 × 10^−1^	3.0199 × 10^−11^
F12	3.0199 × 10^−11^	4.0772 × 10^−11^	3.0199 × 10^−11^	3.0199 × 10^−11^	4.9426 × 10^−5^	3.0199 × 10^−11^	3.0199 × 10^−11^	3.0199 × 10^−11^	3.0199 × 10^−11^
F13	3.0199 × 10^−11^	3.0199 × 10^−11^	3.0199 × 10^−11^	3.0199 × 10^−11^	3.0199 × 10^−11^	3.0199 × 10^−11^	3.0199 × 10^−11^	3.0199 × 10^−11^	3.0199 × 10^−11^
F14	4.5043 × 10^−11^	9.5207 × 10^−4^	5.4617 × 10^−9^	8.1527 × 10^−11^	2.0283 × 10^−7^	4.0772 × 10^−11^	1.6132 × 10^−10^	1.4294 × 10^−8^	3.0199 × 10^−11^
F15	3.0199 × 10^−11^	1.8567 × 10^−9^	3.0199 × 10^−11^	3.0199 × 10^−11^	5.4941 × 10^−11^	3.0199 × 10^−11^	3.0199 × 10^−11^	3.0199 × 10^−11^	3.0199 × 10^−11^
F16	2.7726 × 10^−5^	7.1719 × 10^−1^	3.5547 × 10^−1^	1.3703 × 10^−3^	8.8830 × 10^−1^	3.0199 × 10^−11^	7.6588 × 10^−5^	3.7108 × 10^−1^	3.0199 × 10^−11^
F17	3.3520 × 10^−8^	6.3088 × 10^−1^	2.2658 × 10^−3^	9.9186 × 10^−11^	1.0547 × 10^−1^	3.0199 × 10^−11^	2.0338 × 10^−9^	3.7782 × 10^−2^	3.0199 × 10^−11^
F18	6.2828 × 10^−6^	1.4733 × 10^−7^	3.3520 × 10^−8^	5.4941 × 10^−11^	1.6062 × 10^−6^	3.0199 × 10^−11^	5.4941 × 10^−11^	2.0023 × 10^−6^	3.0199 × 10^−11^
F19	3.0199 × 10^−11^	2.4386 × 10^−9^	3.0199 × 10^−11^	3.0199 × 10^−11^	1.8608 × 10^−6^	3.0199 × 10^−11^	3.0199 × 10^−11^	3.0199 × 10^−11^	3.0199 × 10^−11^
F20	8.7663 × 10^−1^	1.1711 × 10^−2^	5.9706 × 10^−5^	9.5139 × 10^−6^	1.1937 × 10^−6^	9.2603 × 10^−9^	1.5964 × 10^−7^	4.8413 × 10^−2^	3.3384 × 10^−11^
F21	3.0199 × 10^−11^	7.0881 × 10^−8^	5.5727 × 10^−10^	3.0199 × 10^−11^	3.0199 × 10^−11^	3.0199 × 10^−11^	3.0199 × 10^−11^	1.8500 × 10^−8^	3.0199 × 10^−11^
F22	3.5137 × 10^−2^	2.2360 × 10^−2^	2.7086 × 10^−2^	8.0727 × 10^−1^	1.8368 × 10^−2^	8.7663 × 10^−1^	2.9047 × 10^−1^	1.3703 × 10^−3^	1.2870 × 10^−9^
F23	3.0199 × 10^−11^	3.3384 × 10^−11^	3.0199 × 10^−11^	3.0199 × 10^−11^	3.0199 × 10^−11^	3.0199 × 10^−11^	3.0199 × 10^−11^	3.0199 × 10^−11^	3.0199 × 10^−11^
F24	3.0199 × 10^−11^	4.5043 × 10^−11^	3.0199 × 10^−11^	3.0199 × 10^−11^	3.0199 × 10^−11^	3.0199 × 10^−11^	3.0199 × 10^−11^	3.0199 × 10^−11^	3.0199 × 10^−11^
F25	3.0199 × 10^−11^	3.0199 × 10^−11^	3.0199 × 10^−11^	3.0199 × 10^−11^	3.0199 × 10^−11^	3.0199 × 10^−11^	3.0199 × 10^−11^	3.0199 × 10^−11^	3.0199 × 10^−11^
F26	3.0199 × 10^−11^	1.4294 × 10^−8^	3.8202 × 10^−10^	3.0199 × 10^−11^	4.6159 × 10^−10^	3.0199 × 10^−11^	2.9215 × 10^−9^	8.4848 × 10^−9^	3.0199 × 10^−11^
F27	3.0199 × 10^−11^	4.1997 × 10^−10^	3.0199 × 10^−11^	3.0199 × 10^−11^	3.0199 × 10^−11^	3.0199 × 10^−11^	2.6099 × 10^−10^	3.0199 × 10^−11^	3.0199 × 10^−11^
F28	3.0199 × 10^−11^	3.0199 × 10^−11^	3.0199 × 10^−11^	3.0199 × 10^−11^	3.0199 × 10^−11^	3.0199 × 10^−11^	3.0199 × 10^−11^	3.0199 × 10^−11^	3.0199 × 10^−11^
F29	3.0199 × 10^−11^	3.1589 × 10^−10^	4.9752 × 10^−11^	3.0199 × 10^−11^	3.0199 × 10^−11^	3.0199 × 10^−11^	3.0199 × 10^−11^	3.0199 × 10^−11^	3.0199 × 10^−11^
F30	3.0199 × 10^−11^	3.0199 × 10^−11^	3.0199 × 10^−11^	3.0199 × 10^−11^	3.3384 × 10^−11^	3.0199 × 10^−11^	3.0199 × 10^−11^	3.0199 × 10^−11^	3.0199 × 10^−11^

**Table A4:** Statistical *p*-values for 9 algorithms in the CEC2022 (D = 20) evaluation.

ID	HHO	LSHADE	GRO	DBO	HSO	WOA	PSO	GWO	COA
F1	2.5020 × 10^4^	2.0844 × 10^4^	1.1523 × 10^4^	3.4854 × 10^4^	6.9839 × 10^3^	3.5309 × 10^4^	6.1707 × 10^3^	1.4939 × 10^4^	4.8494 × 10^4^
F2	7.9622 × 10^3^	2.0796 × 10^4^	3.3754 × 10^3^	9.4736 × 10^3^	3.5306 × 10^3^	1.4132 × 10^4^	4.1031 × 10^3^	4.5072 × 10^3^	1.4396 × 10^4^
F3	5.6473 × 10^2^	4.5225 × 10^2^	4.6239 × 10^2^	5.1391 × 10^2^	5.6096 × 10^2^	6.4079 × 10^2^	4.8639 × 10^2^	4.9492 × 10^2^	3.1412 × 10^3^
F4	6.2359 × 10^1^	1.6089 × 10^1^	1.1233 × 10^1^	5.4477 × 10^1^	6.8850 × 10^1^	8.1424 × 10^1^	3.8022 × 10^1^	4.2415 × 10^1^	8.0479 × 10^2^
F5	6.6414 × 10^2^	6.0102 × 10^2^	6.0171 × 10^2^	6.3396 × 10^2^	6.3699 × 10^2^	6.6710 × 10^2^	6.1091 × 10^2^	6.0795 × 10^2^	6.8067 × 10^2^
F6	7.1620 × 10^0^	1.4216 × 10^0^	5.7498 × 10^−1^	9.8236 × 10^0^	5.5161 × 10^0^	1.5568 × 10^1^	4.3013 × 10^0^	4.3004 × 10^0^	9.1194 × 10^0^
F7	8.8456 × 10^2^	8.4077 × 10^2^	8.4551 × 10^2^	9.0809 × 10^2^	9.1655 × 10^2^	9.3691 × 10^2^	9.0685 × 10^2^	8.6097 × 10^2^	9.7816 × 10^2^
F8	1.3611 × 10^1^	1.2088 × 10^1^	1.0662 × 10^1^	2.9836 × 10^1^	1.1827 × 10^1^	4.2020 × 10^1^	2.1665 × 10^1^	2.8044 × 10^1^	1.7881 × 10^1^
F9	3.0452 × 10^3^	1.2054 × 10^3^	9.2297 × 10^2^	2.2795 × 10^3^	1.1005 × 10^3^	4.6551 × 10^3^	9.9209 × 10^2^	1.2627 × 10^3^	3.5843 × 10^3^
F10	2.6241 × 10^2^	2.3868 × 10^2^	1.1269 × 10^1^	6.5896 × 10^2^	1.7129 × 10^2^	2.0886 × 10^3^	3.8085 × 10^1^	3.1269 × 10^2^	3.2798 × 10^2^
F11	1.9306 × 10^5^	5.7172 × 10^3^	8.8106 × 10^4^	1.1521 × 10^6^	4.5929 × 10^3^	7.0905 × 10^6^	2.0186 × 10^6^	2.8656 × 10^6^	2.5864 × 10^9^
F12	1.4743 × 10^5^	4.9782 × 10^3^	1.4887 × 10^5^	2.0816 × 10^6^	3.7156 × 10^3^	1.1621 × 10^7^	1.3833 × 10^6^	7.2868 × 10^6^	8.8284 × 10^8^
